# Nano-wrinkle waveguides for programmable polariton channeling

**DOI:** 10.1038/s41467-026-77593-y

**Published:** 2026-09-17

**Authors:** Liyan Wang, Bei Yang, Tian Sun, Cheng Luo, Yuchuan Xiao, Qizhi Yan, Weikang Lu, Ziyi Xu, Zhenghua An, Wei Lu, Xingxing Jiang, Debo Hu, Andrea Alù, Peining Li, Qing Dai

**Affiliations:** 1 CAS Key Laboratory of Standardization and Measurement for Nanotechnology, National Center for Nanoscience and Technology, Beijing, China; 2 School of Materials Science and Engineering, Shanghai Jiao Tong University, Shanghai, China; 3 School of Optical and Electronic Information, Huazhong University of Science and Technology, Wuhan, China; 4 Department of Physics, Fudan University, Shanghai, China; 5 School of Physical Science and Technology, ShanghaiTech University, Shanghai, China; 6 Technical Institute of Physics and Chemistry, Chinese Academy of Sciences, Beijing, China; 7 Advanced Science Research Center, City University of New York, New York, NY USA; 8 Graduate Center, City University of New York, New York, NY USA

**Keywords:** Nanoscience and technology, Nanophotonics and plasmonics

## Abstract

The intrinsic planarity of two-dimensional (2D) van der Waals (vdW) polaritonic media inherently limits the lateral confinement of vdW polaritons. Here, we transcend this geometric limit by introducing a deterministic strategy to engineer non-planar nano-wrinkle waveguides in hexagonal boron nitride (hBN) via controlled thermal buckling. By exploiting the thermal expansion features of a calcite substrate, we generate uniaxial stress during cooling, which naturally forms high-curvature wrinkles in the overlying hBN superstrate. These wrinkles function as reconfigurable channel waveguides, guiding polaritons along predefined paths, and nano-hole patterning of such heterostructures offers programmable routing. Cryogenic near-field imaging and simulations reveal a wrinkle-guided polariton mode with a wavevector and dispersion governed by the wrinkle geometry and distinct from substrate-supported branches. Their hybridization allows precise tuning of modal confinement via hBN thickness variation. Concurrently, these structures guide polariton-mediated heat flow, establishing a medium-free mechanism for nanoscale thermal management. More broadly, our approach establishes a universal route for the geometric control of light and heat in two-dimensional materials.

## Abstract (web summary)

Polaritons (hybrid light-matter excitations) in van der Waals materials hold promise for nanophotonics, but their application is normally hampered by their limited lateral confinement. Here, the authors report a strategy to fabricate deterministic high- curvature wrinkles in hBN films and apply them as controllable polaritonic and thermal waveguides.

## Introduction

Polaritons—quasiparticles formed by coupling light with collective excitations in solids—span a broad class of electromagnetic modes, including plasmon, phonon, and exciton polaritons^1^. These modes enable subwavelength confinement of electromagnetic energy and serve as efficient carriers of information and heat at the nanoscale^2,3^, positioning them as key building blocks for next-generation photonic circuits and thermal transport technologies^4,5^. The emergence of van der Waals (vdW) materials enables low-loss, tunable polaritons that surpass conventional systems based on metals or polar crystals in both propagation length and spectral flexibility^6,7^. In-plane control of vdW polaritons has been demonstrated through momentum-space modulation strategies such as electrostatic gating^8–10^, chemical doping^11–13^, and twist-angle engineering^14–17^. However, the ultrathin, inherently planar geometry of vdW materials is both a blessing and a curse for polaritons. While it greatly facilitates in-plane dispersion control and interfacial coupling of polaritons^18,19^, its in-plane homogeneity prevents lateral confinement, and thus the polaritonic modes cannot form true channel waveguide modes—unlike metal-based plasmonics, where the nanoscale radius of curvature offers tight spatial control^20–22^. Consequently, directional routing of vdW polaritons remains a persistent challenge, constrained by the intrinsic planarity of vdW materials and the absence of controllable methods to fabricate highly curved vdW surfaces with nanoscale radii of curvature commensurate with polariton wavelengths^23,24^.

Here, we overcome this limitation by introducing deterministic thermal buckling to realize geometrically defined polariton waveguides in vdW heterostructures. By exploiting the hyperbolic thermal expansion mismatch between a hexagonal boron nitride (hBN) thin film and a calcite substrate, we induce uniaxial interfacial stress during cooling, triggering the formation of suspended nano-wrinkles with controlled orientation and curvature. These wrinkles act as nanoscale channels that guide hBN phonon polaritons along predefined paths. Near-field imaging and simulations reveal that these structures support a guided polariton mode. The wrinkle-on-substrate platform introduces hybridization between wrinkle-guided and substrate-supported modes as an additional degree of freedom, offering opportunities for tuning modal confinement. In addition to photonic control, the same architecture can enable directional, polariton-mediated heat transport, offering a medium-free, low-loss mechanism for nanoscale thermal routing. This wrinkle-guided approach establishes a generalized strategy for incorporating geometric curvature into vdW materials, with potentially broad implications for reconfigurable nanophotonics and cryogenic thermal management in quantum technologies.

## Results

### Deterministic thermal buckling of hBN to form nano-wrinkles

VdW heterostructures (vdWHs) are generally susceptible to thermal expansion misfit^25–27^. Therefore, the wrinkling instability of vdW nanosheets driven by interfacial thermal stress can be used to prepare high-curvature nano-wrinkles for channeling vdW polaritons. Since vdW materials usually possess negative in-plane thermal expansion coefficients (TECs)^28–30^, a substrate with both hyperbolic in-plane thermal expansion and high optical quality is ideally suited for generating uniaxial compressive stress at the vdW/substrate interface upon cooling. Calcite crystals are known for their large positive TEC in the direction parallel to the optic axis (*α*_∥_) and a small negative one in the plane normal to the optic axis (*α*_⊥_)^31–35^, thus, calcite with a properly cut crystal surface constitutes a suitable substrate for our purpose.

**Fig. 1:**
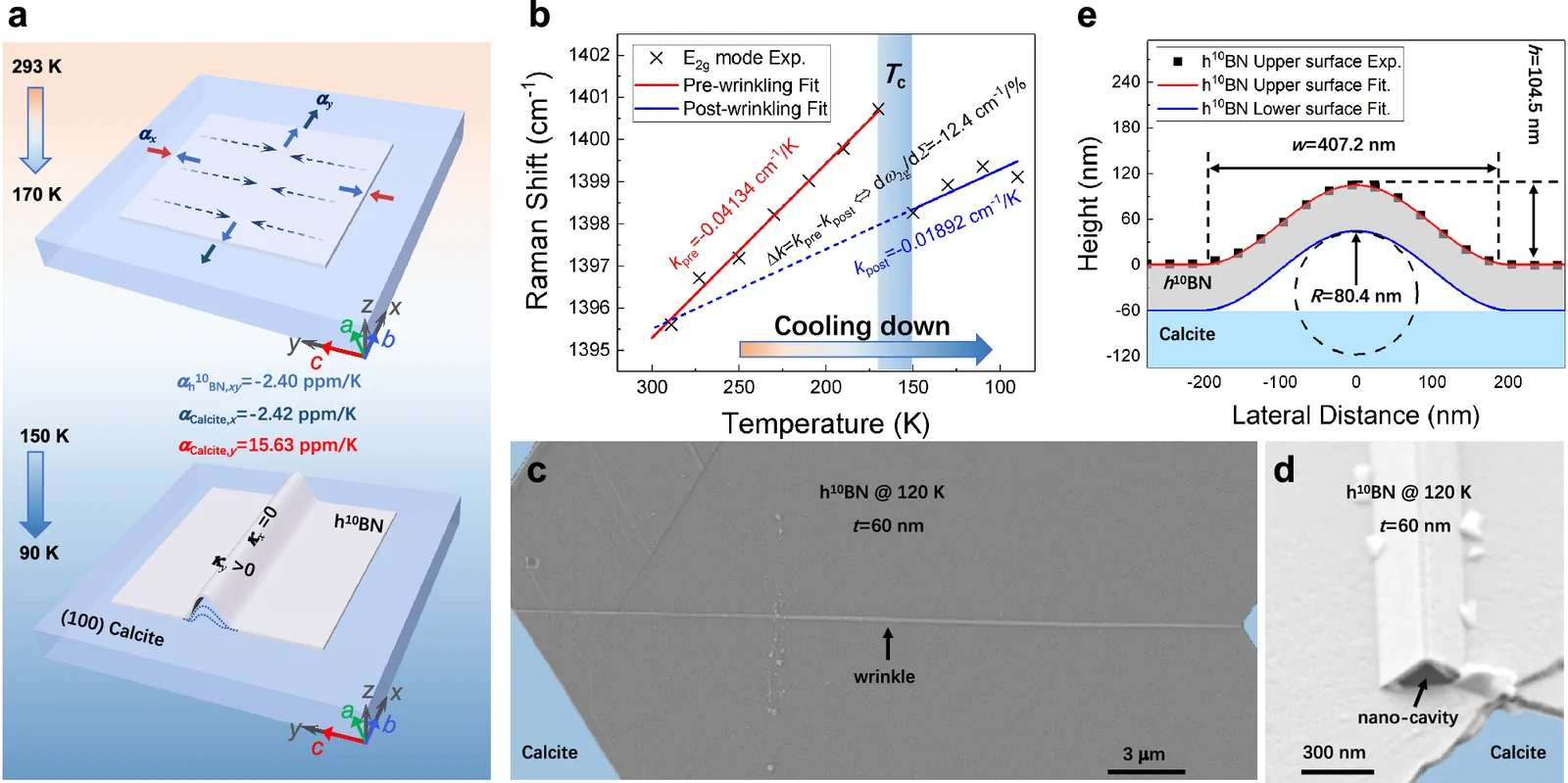
Thermal buckling induced nano-wrinkles with pre-defined in-plane orientation. **a** Diagram of the proposed scheme for preparing buckling van der Waals (vdW) surfaces using the uniaxial thermal stress at the interface between an isotopically purified hexagonal boron nitride (h^10^BN) nanosheet and a (100)-cut calcite substrate upon cooling. *α*_h_^10^_BN,xy_, *α*_calcite,x_, and *α*_calcite,y_ denote the in-plane thermal expansion coefficients of h^10^BN and those of calcite along the *x*- and *y*-axes, respectively. **b** Monitoring the wrinkling process of a h^10^BN nanosheet using variable-temperature Raman spectroscopy. Data points represent the measured E_2g_ phonon frequencies of h^10^BN as a function of temperature. Solid lines are linear fits to the data above and below the critical buckling temperature *T*_c_ (the temperature at which the h^10^BN flake begins to buckle out-of-plane). The blue shaded region indicates the temperature range over which the buckling transition occurs. **c** Scanning electron microscopy (SEM) image of a long and straight wrinkle running through the h^10^BN nanosheet from end to end, the calcite parts in image have been colorized. The thickness of the h^10^BN flake is denoted as *t*. **d** Tilted-view SEM image of a wrinkle that reveals the nano-cavity enclosed between the curved nanosheet and the calcite substrate which has also been colorized. **e** Cross-sectional height profile (data points) of the wrinkle in (**d**) obtained by atomic force microscopy (AFM) at 120 K and fitted curves for the upper surface (red line) and lower surface (blue line) of h^10^BN. *h, w*, and *R* are the height, width, and radius of curvature of the wrinkle, respectively.

The measured temperature-dependent cell parameters of calcite using powder X-ray diffraction (XRD) are shown in Supplementary Fig. 1. By fitting the data with linear models, *α*_⊥_ and *α*_∥_ of calcite are found to be −2.42 ppm/K and 15.63 ppm/K, respectively, within the temperature range from 100 K to 400 K. Noting that the average in-plane TEC of hBN in the same temperature interval is nearly identical to *α*_⊥_ of calcite (for natural hBN *α*_hBN_ = −2.48 ppm/K and for isotopically purified h^10^BN *α*_h_^10^_BN_ = −2.40 ppm/K)^36–38^, and the most readily available anisotropic crystal surface of calcite is (100), we choose h^10^BN and (100)-cut calcite to assemble vdWHs with a large in-plane uniaxial thermal expansion mismatch (the in-plane TECs of calcite substrate are −2.42 and 15.63 ppm/K along the *x* and *y* axes respectively) as illustrated in Fig. 1a. The as-prepared h^10^BN/calcite heterostructures are planar at room temperature; when cooled down, the h^10^BN nanosheet tends to stretch isotropically in the *x*-*y* plane while the calcite substrate extends almost synchronously with h^10^BN along the *x* axis and contracts along the *y* axis. The net result is a highly directional uniaxial compressive stress acting on h^10^BN indicated schematically in Fig. 1a, which induces a compression strain *Σ* within the nanosheet along the *y* axis, forcing the h^10^BN to buckle solely perpendicular to the stress field. The temperature-dependent strain energy competes with the interfacial vdW cohesion energy and surpasses it below a critical temperature, triggering an elastic instability and forming nano-wrinkles of h^10^BN nanosheet.

This envisaged wrinkling process can be monitored and confirmed using variable-temperature Raman spectroscopy, because the E_2g_ phonon frequency of h^10^BN *ω*_2g_ depends on both temperature and strain. As presented in Fig. 1b, the data points corresponding to *ω*_2g_ at different temperatures are distinctly divided into two groups by the 170–150 K interval. By fitting these two groups of data linearly, we obtain the pre- and post-buckling Raman shift rates *k*_pre_ = −0.04134 cm^−1^/K and *k*_post_ = −0.01892 cm^−1^/K, respectively. Notably, *k*_pre_ departs dramatically from the Raman shift rate caused purely by temperature, *k* = −0.019 cm^−1^/K extracted from previously reported data^39^, while *k*_post_ matches *k* very well. These results indicate several interesting features: the Raman shifts above 170 K are contributed by both temperature and strain; the h^10^BN nanosheet wrinkles at a critical temperature within the 170-150 K interval and releases the accumulated strain completely; the Raman shifts below 150 K are purely attributed to temperature thus further contraction of the substrate and expansion of the nanosheet at lower temperatures than the critical one must have been accommodated by the enlargement of existing wrinkles as indicated in the lower panel of Fig. 1a. Assuming that there is no relative slip between nanosheet and substrate in the pre-buckling stage, the calculated strain-induced Raman shift rate (d*ω*_2g_/d*Σ*=−12.4 cm^−1^/%) is in close agreement with the former reported one (−11.9 cm^−1^/%) for natural hBN^40^, providing rigorous validation for our wrinkle formation model.

A variable-temperature scanning electron microscope (SEM) is used to image the h^10^BN/calcite assembly at cryogenic temperatures. Top-view (Fig. 1c) and tilted-view (Fig. 1d) SEM images show that nano-wrinkles do appear at low temperatures, and the h^10^BN nanosheets delaminate from the substrate at wrinkle crests. As shown in Fig. 1e, the cross-section profile of the wrinkle in (d), obtained by atomic force microscope (AFM) at 120 K, fits well with the sinusoidal model^41^ *z*(*y*) = *h*/2{1+cos[2π(*y*-*y*_c_)/*w*]}, where *h* is the height, *w* is the width of the wrinkle, and *y*_c_ is the coordinate of the wrinkle crest. The radius of curvature *R* at the wrinkle crest^42^ is estimated to be 80.4 nm via *R* = *w*^2^/2π^2^*h*, and one of the principal curvatures of the wrinkle crest is determined to be *κ*_y_ = 1/*R*. Since the wrinkled h^10^BN nanosheet (strain-released) is an isometric transformation of the planar as-prepared one (strain-free), Gauss’s Theorema Egregium requires that the Gaussian curvature *K* = *κ*ₓ*κ*_y_ be zero everywhere on the surface^43^. Therefore, *κ*_x_ = 0, the wrinkled surfaces should be developable (generalized cylinders)^44^, and the nano-wrinkles have to be straight along the *x*/**b** axis. Remarkably, this geometric inference matches well the SEM observation in Fig. 1c.

To spatially program wrinkle nucleation, we perforate h^10^BN nanosheets and use the resultant nano-holes (see Methods for fabrication details) to intensify local thermal stress and weaken, at the same time, interfacial cohesion in the vicinity of these holes^45–47^. This increase in strain energy and decrease in cohesion energy make the neighboring regions of nano-holes preferred locations for h^10^BN wrinkling. As displayed in Supplementary Fig. 2, our experiments work as designed: with one hole of a radius *r* = 100 nm drilled through a 40-nm-thick h^10^BN sheet (Supplementary Fig. 2a), an 80-nm-high (at 90 K) wrinkle runs through the whole sheet from end to end via the hole (Supplementary Fig. 2b); with an array of holes in a 55-nm-thick h^10^BN sheet arranged along the *x* axis (Supplementary Fig. 2c), wrinkle segments with an average height of 23.5 nm (at 100 K) connect the adjacent holes (Supplementary Fig. 2d).

We further investigate the temperature dependence of the wrinkle geometrical parameters by taking the wrinkle segments in Supplementary Fig. 2c as an example. As shown in Supplementary Fig. 3, the average height of wrinkles *h* decreases linearly with increasing temperature, and the wrinkles’ average radius of curvature *R* increases nonlinearly in the same temperature range, as a result of the temperature-insensitive wrinkle width *w* and the inverse relationship between *h* and *R*. This linear dependence of wrinkle *h* enables programmable control over wrinkle geometry via thermal tuning. According to the trends of the fitted curves in Supplementary Fig. 3, *h* should be zero while *R* approaches infinity at about 246 K. The AFM imaging result at 250 K in Supplementary Fig. 2c indeed meets these expectations by presenting a completely planar surface. Note that the onset wrinkling temperature in this case (246 K) is higher than the one in Fig. 1b (<170 K); this is probably due to the rise of the strain-cohesion balance temperature caused by the appearance of nano-holes.

To systematically show the reversibility and repeatability of the wrinkle formation process, multiple cooling-heating cycles are performed on a graphite/calcite heterostructure. Variable-temperature AFM imaging (Supplementary Fig. 4a) demonstrates the reversibility: wrinkles vanish as the film returns to a flat state upon warming above the critical temperature and re-form at nearly identical locations upon subsequent re-cooling. The wrinkles deterministically repeat their spatial positions across four thermal cycles (Cycle-1 to Cycle-4), confirming that nucleation is governed by interfacial properties. Furthermore, the wrinkle geometry (height and width) is both tunable and reproducible under controlled thermal conditions. A larger temperature drop (e.g., cooling from 473 K in Cycle-2, Supplementary Fig. 4b) produces a larger wrinkle, while re-applying the same thermal history (Cycle-4) yields dimensions that closely match those of a prior cycle (Cycle-2), with variations within ~5%. These findings establish that the thermal-buckling process is reversible, spatially deterministic, and geometrically controllable, highlighting its potential for creating reconfigurable nanophotonic elements.

### Directional channeling of hBN polaritons by nano-wrinkles

**Fig. 2:**
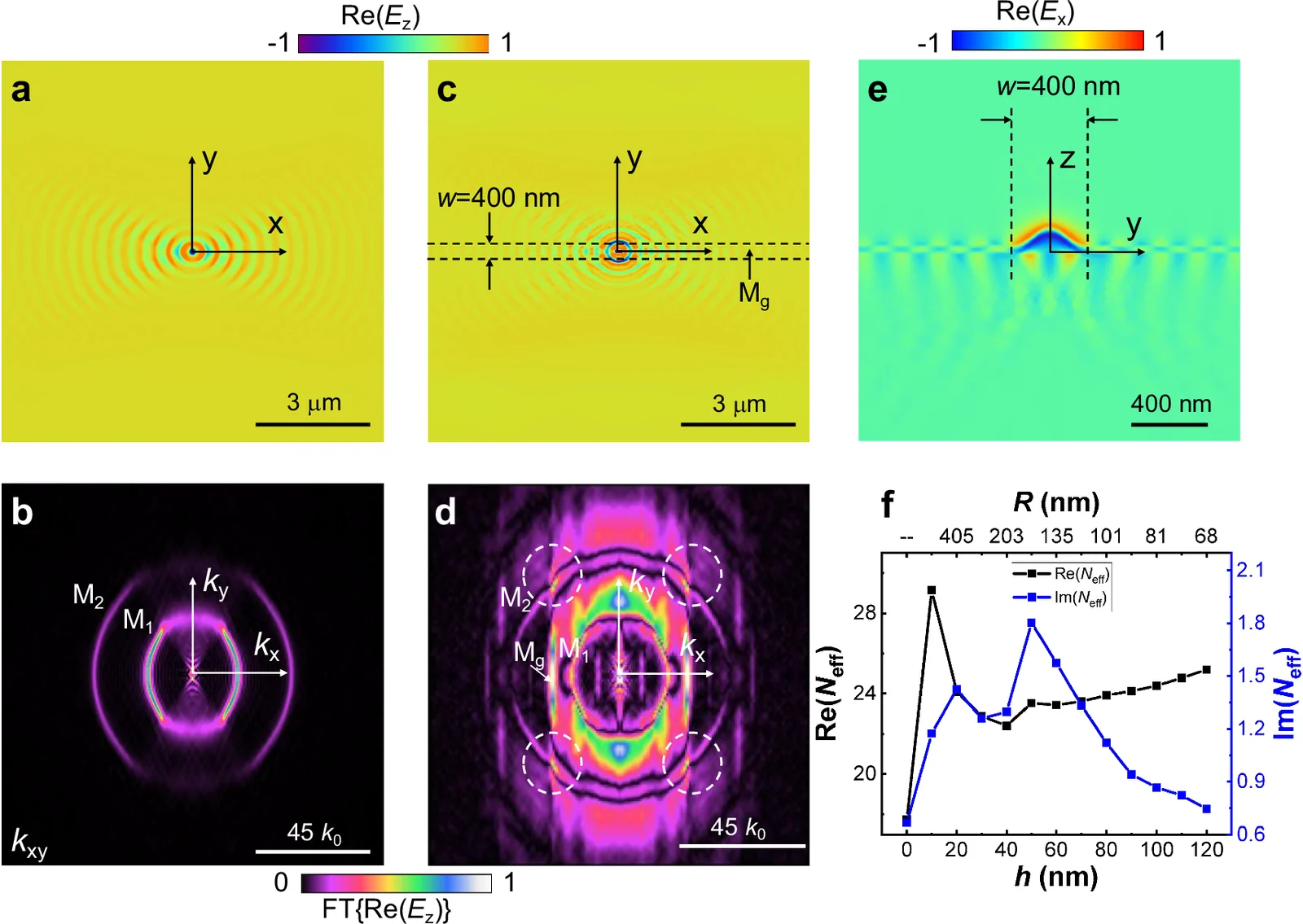
Channeling of a phonon polariton mode (M_g_) by a wrinkled h^10^BN nanosheet at the (100) surface of calcite. **a** A simulated snapshot of the polariton wavefronts launched by a *z*-oriented dipole located 100-nm above the surface of a 60-nm-thick h^10^BN flake, the excitation frequency is 1460 cm^−1^. **b** Fourier transform (FT) of (**a**), indicating at least two modes (M_1_ and M_2_ with increasing in-plane wavevectors *k*_xy_) have been excited by the dipole. **c** Simulated wavefronts at the surface of a wrinkled h^10^BN nanosheet, the width of the wrinkle *w* is 400 nm and the height *h* is 100 nm, the dipole locates 100-nm above the crest of the wrinkle. The wrinkle-guided phonon polariton mode (M_g_) propagates in the ±*x* direction with a much shorter wavelength than that of M_1_. **d** Fourier transform of (**c**), indicating the M_1_ mode in the *x* direction has been transformed into M_g_ mode with a much larger wavevector. The circled region highlights the overlap between the wrinkle-guided M_g_ mode and the substrate-supported M_2_ mode. **e** Cross-sectional mode profile of M_g_ in (**c**), the in-plane component of the optical field *E*_x_ is shown. **f** Dispersion of the M_g_ mode at 1460 cm^−1^ against increasing *h* (decreasing *R*) and a fixed *w* = 400 nm. **a** and **c** share the same false color ruler, (**b**) and (**d**) share the other. *N*_eff_ is the effective index of refraction of the M_g_ mode.

As shown in Supplementary Fig. 5, the directional channeling effect of a nano-wrinkle can be best demonstrated by comparing the polariton propagation in a flat and a wrinkled h^10^BN nanosheet, both assumed to be freestanding to eliminate ambiguities resulting from the substrate. However, a highly curved wrinkle in a freestanding h^10^BN nanosheet would not be stable. Therefore, we evaluate the impact of introducing the calcite substrate. As shown in Fig. 2a, the in-plane hyperbolic optical response of the (100)-cut calcite (*ε*_x_ = -5.02 + 0.79i and *ε*_y_ = 2.34 at the frequency 1460 cm^-1^) forbids the h^10^BN polaritons from propagating in a specific in-plane angle range with respect to the *y* axis. This spatial filtering effect can be seen clearly in the two-dimensional (2D) Fourier transform of (a) as shown in Fig. 2b. Quantitative analyses of Fig. 2b show the wavevectors in the *x* direction (real parts) of polariton modes M_1_ and M_2_ are *k*_M1x_ = 17.73*k*_0_ and *k*_M2x_ = 38.97*k*_0_, respectively. Wrinkling of the h^10^BN nanosheet (400 nm width and 100 nm height) breaks the in-plane symmetry of the heterostructure, leading to a redistribution of polariton wavefronts in real space (Fig. 2c) and a reconfiguration of the isofrequency contours (Fig. 2d). This geometric perturbation gives rise to a distinct M_g_ mode (clearly resolved in filtered real-space images with M_1_ and M_2_ components filtered out, Supplementary Fig. 6), which propagates predominantly along the ±*x* direction with an in-plane wavevector of 24.58*k*_0_, clearly separated from the substrate-supported M_1_ and M_2_ modes. Cross-sectional field profiling confirms that the M_g_ mode is localized within the suspended wrinkle region (Fig. 2e), consistent with a geometry-confined propagation channel. The guided nature of the M_g_ mode is reflected in its systematic dependence on wrinkle geometry, with both the real and imaginary parts of the effective index *N*_eff_ evolving continuously as functions of wrinkle height *h* and width *w* (Fig. 2f and Supplementary Fig. 7). Notably, for *h* > 50 nm, the M_g_ mode exhibits nearly identical dispersion trends in both suspended and substrate-supported configurations. Collectively, these observations—its distinct wavevector, characteristic isofrequency contour, and geometry-dependent dispersion—support the interpretation of M_g_ as a wrinkle-guided channeling mode.

While the existence of the M_g_ mode is determined by the wrinkle geometry, its confinement is influenced by coupling to substrate-supported modes. As shown in Fig. 2d, the isofrequency contours of M_g_ partially overlap with those of the second-order substrate-supported mode M_2_, inducing hybridization between the two branches. This hybridization redistributes polariton energy, thereby providing a route to engineer modal confinement through h^10^BN thickness tuning. As shown in Supplementary Fig. 8, reducing the h^10^BN thickness from 60 nm to 30 nm shifts the M_g_ and M_2_ modes to larger wavevectors and decreases the overlap of their isofrequency contours, thereby weakening mode hybridization and increasing the M_g_ mode confinement ratio *η* to 93.1%.

**Fig. 3:**
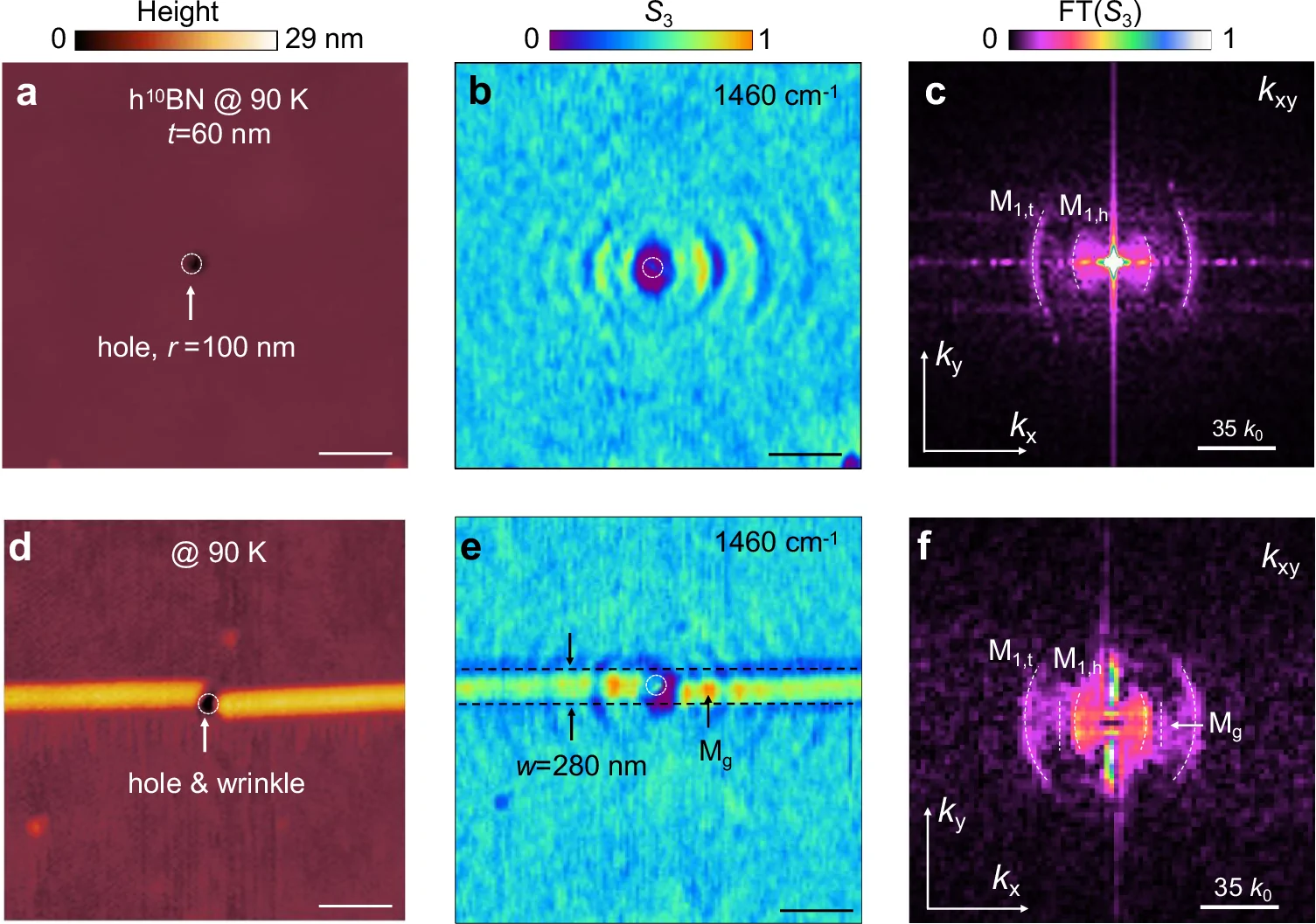
Real-space imaging of the channel vdW polaritons (M_g_) in wrinkled h^10^BN/calcite heterostructures. **a** AFM topography of a 60-nm-thick h^10^BN/calcite heterostructure with a 100-nm-radius hole. **b** Near-field amplitude image (*S*_3_) of polaritons in (**a**), with an excitation frequency of 1460 cm^−1^. **c** Fourier transform of the image in (**b**), which indicates two modes (M_1, t_ and M_1, h_) have been excited. M_1, h_ is excited by the hole edge, and M_1, t_ by the tip. **d** AFM topography of a 60-nm-thick /calcite heterostructure with a nano-wrinkle at 90 K. **e** Near-field image of polaritons on a wrinkled h^10^BN nanosheet of (**d**), the width of the wrinkle *w* is 280 nm and the height *h* is 29 nm. **f** Fourier transform of (**e**), indicating the M_1_ mode in the *x* direction has been transformed into M_g_ mode with a much larger wavevector by the nano-wrinkle. (**a**), (**b**), (**d**) and (**e**) share the same scale bar: 500 nm. The color scales are shared between the following: (**a**) and (**d**); (**b**) and (**e**); and (**c**) and (**f**).

To experimentally validate this channeling effect of phonon polaritons through h^10^BN nano-wrinkles, we performed nano-infrared imaging of h^10^BN/calcite heterostructures using cryogenic scattering-type scanning near-field optical microscopy (cryo-SNOM). Figures 3a, b display the AFM topography and near-field image, respectively, of a 60-nm-thick h^10^BN/calcite heterostructure at 1460 cm^−1^, revealing arc-shaped wavefronts propagating along the *x*-axis and standing-wave patterns from boundary reflections at the nanohole edge. 2D Fourier transform analysis (Fig. 3c) resolves two distinct polariton modes (M_1,h_ and M_1,t_) with real wavevector components along the *x*-direction: *k*_M1,hx_ = 19.17*k*_0_ and *k*_M1,tx_ = 39.72*k*_0_. The *k*_M1,hx_ value matches the simulated first-order mode (*k*_M1x_ = 17.73 *k*_0_), confirming its origin from hole-edge excitation. Notably, *k*_M1,tx_/*k*_M1,hx_ ≈ 2 aligns with the coherent superposition model of tip-launched polaritons and their boundary-reflected counterparts. With the introduction of nano-wrinkles (*h* = 29 nm, *w* = 280 nm, as shown in Fig. 3d), the polariton propagation characteristics are significantly modulated. Figure 3e demonstrates waveguide-like transmission along the wrinkle axis (the ±*x*-direction), while Fourier analysis (Fig. 3f) identifies the wrinkle-induced M_g_ mode with *k*_Mgx_ = 23.82*k*_0_ (closely matches simulated *k*_Mgx_ = 23.49*k*_0_, as shown in Supplementary Fig. 9), positioned between M_1,h_ and M_1,t_ in momentum space. By applying 2D FFT filtering to Fig. 3e, the resulting real-space near-field image (Supplementary Fig. 10b) clearly identifies the M_g_ mode. The line profile across the wrinkle shows a single periodic fringe corresponding to this mode, with a fitted quality factor of *Q* = Re(*k*_p_)/Im(*k*_p_)=14.9, corresponding to a 1/e amplitude decay length of *L* = 0.68 μm. For comparison, line profiles from the flat region outside the wrinkle resolve two distinct fringe periods corresponding to M_1,h_ (*Q* = 27.4, *L* = 1.56 μm) and M_1,t_ (*Q* = 18.1, *L* = 0.50 μm), confirming that the M_g_ mode carries propagation characteristics distinct from the conventional substrate-supported modes (Supplementary Fig. 10c). The experimentally observed wavevector magnitudes for both planar and wrinkled geometries are systematically lower than the theoretical predictions. This slight discrepancy likely originates from the nonlinear piezo-response in the cryo-SNOM scanner calibration.

To correlate wrinkle morphology with polariton dispersion, we performed a frequency-response analysis on a multi-wrinkle system induced by hole arrays. Supplementary Fig. 11a shows the AFM topography of the system with five holes (No. 1–5), where two horizontally connected wrinkles form between holes No. 1–4. Polariton interference fringes appear at all holes (Supplementary Fig. 11b–f), while polaritons confined at wrinkles exhibit fringe spacings dependent on wrinkle dimensions (i.e., distinct polariton wavevectors). Supplementary Fig. 11g systematically presents the experimental dispersion of wavevectors extracted from wrinkles adjacent to each hole. Quantitative analysis of the real part of the polariton wavevector Re(*k*_Mgx_) of M_g_ across 1430–1470 cm^−1^ demonstrates its strong dependence on wrinkle height, indicating the programmable dispersion of the guided polariton mode—and hence the waveguide’s optical function—through wrinkle geometry. When the wrinkle height increases from 7 nm to 29 nm, Re(*k*_Mgx_) exhibits a non-monotonic variation, consistent with simulated trends in Fig. 2f. This complex dependence arises from the interplay between wrinkle geometry and substrate dielectric perturbations. For *h* < 50 nm, residual coupling to the calcite substrate persists, causing the wavevector of the confined M_g_ mode to approach values characteristic of unperturbed substrate-coupled polaritons. As the wrinkle height increases, the M_g_ mode gradually decouples from the substrate, leading to reduced dielectric perturbation and a corresponding decrease in Re(*k*_Mgx_). The transition from substrate-dominated to geometry-dominated confinement manifests as a linear dependence of Re(*k*_Mgx_) on *h*, reflecting the diminishing influence of residual perturbations at larger wrinkle heights.

### Channeling heat flow by nano-wrinkles

**Fig. 4:**
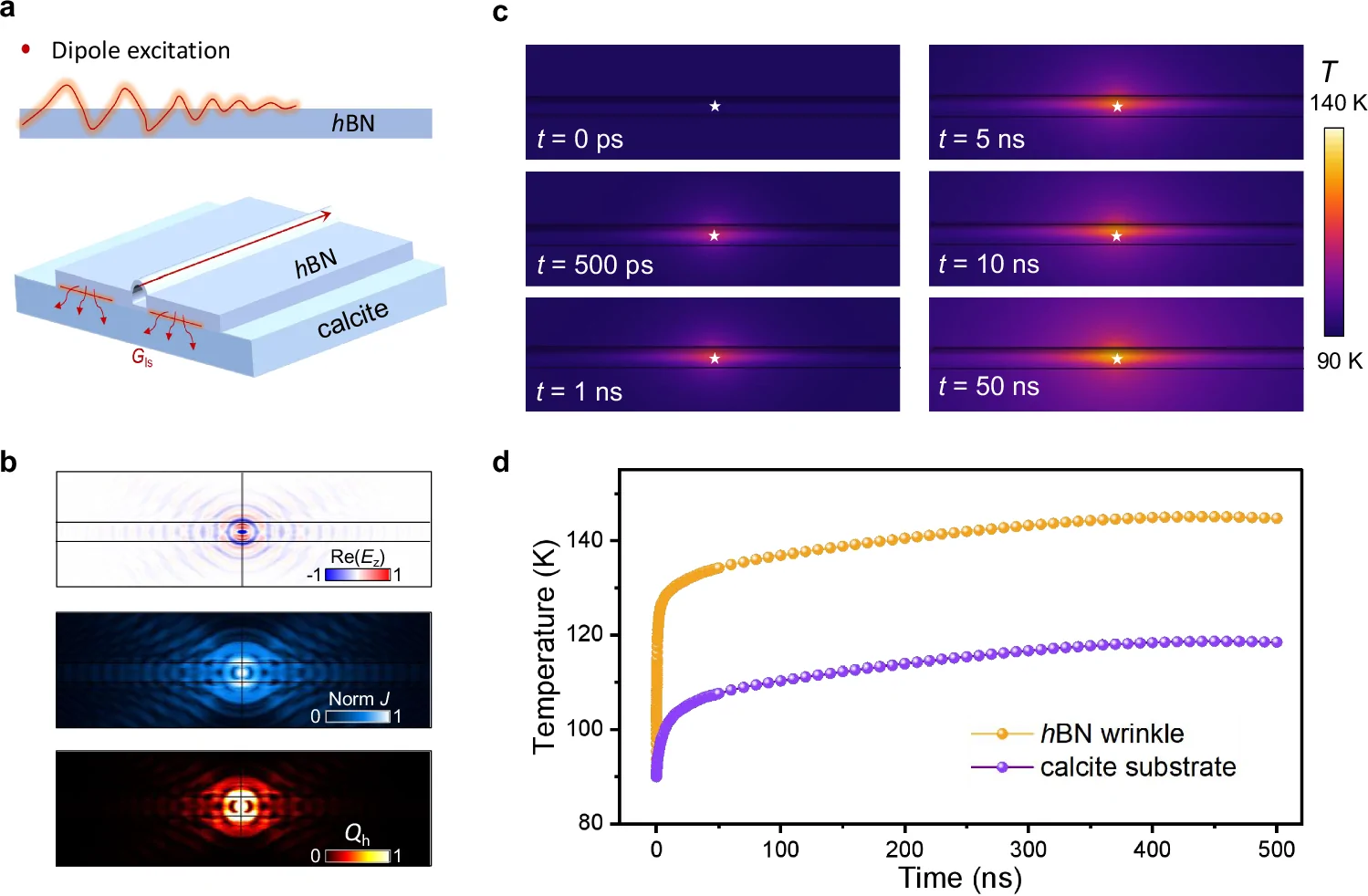
Directional heat transport mediated by channel polaritons of wrinkled h^10^BN. **a** Schematic of channel vdW polariton-mediated heat transport. Polariton dissipation is the sole heat source; heat losses include radiative dissipation to the environment and conductive loss to the substrate. The interfacial thermal conductance *G*_Is_ accounts for heat exchange through conduction at the interface between supported h^10^BN and the calcite substrate. **b** Simulated distributions under continuous point dipole excitation (white star, *ω* = 1460 cm^−1^): electric field component Re(*E*_z_) (top), total current density *J* (middle), and electromagnetic thermal power density *Q* (bottom) in the *x-y* plane. **c** Simulated transient temperature evolution in the h^10^BN layer, showing heat transport along the wrinkle via channel polaritons. **d** Simulated temporal evolution of peak temperature in the wrinkled h^10^BN region and calcite substrate.

To validate the directional heat transport capabilities of the channel vdW polaritons, we investigated the coupled optical and thermal responses within wrinkled h^10^BN/calcite heterostructures under mid-IR dipole excitation (1460 cm^−1^), as depicted in Fig. 4a. The simulated electric field distribution (Fig. 4b, top) reveals pronounced field enhancement concentrated within the wrinkle structure, confirming the efficient excitation and spatial confinement of the channel polariton mode (M_g_) propagating along the strain-defined pathway. This confinement is further corroborated by the spatial overlap between the distributions of current density and electromagnetic power loss density, both showing maxima within the wrinkle region (Fig. 4b, middle and bottom). The alignment of these quantities provides direct evidence that dipolar polariton oscillations are responsible for localized heat generation, highlighting the wrinkle as an effective conduit for polariton-mediated directional energy transport.

Transient thermal simulations (Fig. 4c) capture the anisotropic evolution of temperature following continuous excitation by an electric point dipole. Within 500 ps, heat generation initiates at the dipole location and subsequently propagates directionally along the wrinkle axis, consistent with the guided energy transport via channel polaritons. The temporal evolution of the peak temperature within the suspended wrinkle region (Fig. 4d) reveals rapid heating from an initial 90 K to a steady-state maximum of approximately 140 K (a Δ*T* of ≈50 K) within 400 ns. Crucially, when the wrinkle height exceeds the critical decoupling threshold (~50 nm), the dominant vertical heat-sinking pathway—direct interfacial conduction to the substrate—is effectively removed. The remaining heat dissipation occurs primarily via lateral conduction through the h^10^BN film toward the supported regions and then into the substrate, a pathway inherently limited by the series of thermal resistances (lateral spreading, finite interfacial conductance, and substrate diffusion). This results in suppressed substrate heat sinking for the suspended segments, as is evident from the distinct steady-state temperature distributions in Supplementary Fig. 12: suspended h^10^BN regions show significantly elevated temperatures (Supplementary Fig. 12a), while the calcite substrate beneath these wrinkles exhibits negligible heating (Supplementary Fig. 12b). Correspondingly, the maximum substrate temperature rise (~25 K, shown in Fig. 4d) is confined to regions interfacing supported h^10^BN areas. This stark contrast highlights the spatially modulated heat dissipation governed directly by the wrinkle geometry and the degree of substrate decoupling.

Importantly, the suspended wrinkle architecture simultaneously minimizes substrate-mediated interfacial thermal dissipation and promotes efficient in-plane heat conduction pathways along the buckling axis. The nano-wrinkle significantly enhances the electromagnetic field confinement of the M_g_ mode, amplifying the associated electromagnetic field and, consequently, the polariton‑mediated heat generation and the subsequent directional heat flow. This synergy—between wrinkle-induced polariton confinement and suppressed thermal leakage to the substrate—establishes a robust, directional, and low‑thermal‑leakage mechanism for nanoscale heat routing. This approach offers a reconfigurable platform for advanced thermal management in quantum circuits at cryogenic temperatures—potentially resolving thermal bottlenecks in cryo-CMOS and advancing phonon engineering for extreme thermal management^48,49^.

## Discussion

The universality of our thermal buckling strategy can be demonstrated by exploring its applicability to other vdW materials. In graphite nanosheets exhibiting similar negative in-plane thermal expansion with hBN, we experimentally observed analogous wrinkle instability patterns (Supplementary Fig. 13a–c). However, graphene plasmon polaritons exist only in monolayer/few-layer systems, where extreme thinning critically weakens mechanical strength. This mechanical vulnerability would induce mixed strain-curvature responses to uniaxial stress, resulting in non-isometric transformations of the original plane that violate Gauss’s Theorema Egregium. Consequently, graphene wrinkles exhibit poor linearity and discontinuous topography (Supplementary Fig. 13d), rendering them unsuitable for plasmon control. To overcome this limitation, we propose hBN/calcite composite substrates. Here, hBN wrinkles impose synchronized deformation on the overlying graphene, generating uniform, directional wrinkles. Finite-element simulations confirm these engineered structures effectively guide graphene plasmon polaritons (Supplementary Fig. 14). This methodology extends universally: transition metal dichalcogenides and other ultrathin vdW materials comply with substrate deformations due to their low flexural rigidity. By leveraging composite heterostructures, we could achieve deterministic control of polaritons across all vdW systems, establishing thermal buckling as a universal platform for geometrically tailored polaritonics.

Besides, this thermal expansion mismatch approach can likewise be applied to substrates with different symmetries. By altering the symmetry of the stress field, the wrinkle morphology can be actively controlled. For example, on an isotropic substrate like SiO₂, isotropic compression leads to the formation of a two-dimensional triple-symmetry wrinkle network, as observed in our experiments (Supplementary Fig. 15). This transition from 1D channels to 2D networks may enable interconnected polariton circuits or tunable photonic metasurfaces.

While curvature provides a powerful instrument to manipulate light in curved two-dimensional space^50^, the inherently planarized geometry of vdW materials precludes many opportunities in manipulating the underlying polaritons. Our study establishes deterministic thermal buckling as a universal platform for manipulating vdW polaritons through geometrically defined nanoscale waveguides. By leveraging the anisotropic thermal expansion mismatch between h¹⁰BN nanosheets and calcite substrates, we generate controlled interfacial stress that drives wrinkling instabilities upon cooling, producing suspended nano-wrinkles with radii of curvature comparable to polariton wavelengths. The orientation of these strain-engineered nano-wrinkles is dictated by the principal axes of calcite’s hyperbolic thermal expansion, while their spatial localization is deterministically achieved via prefabricated nano-holes acting as stress concentrators. Using cryo-SNOM imaging and full electromagnetic simulations, we demonstrate that these buckled surfaces operate as channel waveguides sustaining wrinkle-guided polariton modes that propagate along predefined directional pathways. The wrinkle-guided mode exhibits dispersion distinct from that of substrate-supported branches, and hybridization between these two modes provides a versatile route to engineer modal confinement via h^10^BN thickness tuning. Beyond photonic control, this platform facilitates polariton-mediated directional heat transport: the suspended-wrinkle geometry minimizes interfacial thermal dissipation to the substrate, while the channel vdW mode enables rapid anisotropic energy flow, thereby establishing a medium-free heat pipe mechanism. The universality of this approach extends to other vdW materials, unifying strain engineering with nanophotonic design for reconfigurable polariton circuits and thermal management at cryogenic temperatures.

## Methods

### X-ray diffraction

The temperature-dependent cell parameters of calcite were investigated using powder X-ray diffraction (XRD). High-purity calcite samples were ground into fine powders to ensure uniformity and placed in a sample holder designed for in-situ measurements. The XRD measurements were performed using a Rigaku SmartLab 9 kW X-ray diffractometer, equipped with a Cu-Kα radiation source (*λ* = 1.5406 Å) and a temperature-controlled chamber. The temperature range for the measurements was set from 100 K to 400 K, with increments of 30 K. The diffraction patterns were collected over a 2θ range of 3° to 80° with a step size of 0.02° and a counting time of 1 s per step. The cell parameters were extracted from the diffraction data using the Rietveld refinement method with the FullProf software package. This approach allowed for the precise determination of the lattice parameters (*a*, *b*, *c*) and the unit cell volume as a function of temperature. The thermal expansion coefficients (*α*_⊥_ and *α*_∥_) were subsequently calculated from the temperature-dependent cell parameters.

### Variable-temperature Raman spectroscopy

The wrinkling process in h^10^BN/calcite heterostructure was monitored and confirmed using variable-temperature Raman spectroscopy. The Raman spectra of h^10^BN were acquired using a confocal Raman microscope (HORIBA, LabRAM HR Evolution). The measurements were performed using a 532 nm laser excitation source. The laser power was carefully controlled with the built-in multi-stage attenuator to avoid sample heating during measurements. The temperature of the sample was varied using a temperature-controlled stage, ranging from 50 K to 200 K. At each temperature setting, the sample was stabilized for 20 min to ensure thermal equilibrium. The Raman spectra were collected in the range of 1000–2000 cm^−1^, with a spectral resolution of 1 cm^−1^. The E_2g_ phonon frequency of h^10^BN was identified and analyzed as a function of temperature. Since the E_2g_ phonon frequency is sensitive to both temperature and strain, the observed frequency shifts were used to infer the extent of wrinkling and the associated strain in the h^10^BN/calcite samples.

### Cryo-scanning electron microscopy (cryo-SEM) imaging

The morphology and structure of the wrinkles were investigated using an ultra-high-resolution cold field emission scanning electron microscope (HITACHI S-3000N&Quorum PP3000T). The samples were mounted on cryo-stage holders and transferred to the SEM chamber via a cryo-transfer system, which maintained a low-temperature environment (down to –160 °C) to prevent thermal damage. The SEM was operated at an accelerating voltage of 5 kV. For top-view imaging, the samples were positioned perpendicular to the electron beam, allowing for a direct view. For tilt-view imaging, the samples were tilted at an angle of 45° relative to the electron beam to provide a three-dimensional perspective of the h^10^BN wrinkles.

### Fabricating nano-hole arrays

First, h^10^BN nanosheets were mechanically exfoliated from natural crystals onto SiO₂ (300 nm)/Si substrates. Subsequently, to define programmable nucleation sites for wrinkles, periodic arrays of nano-holes were patterned into the h^10^BN flakes using 100 kV electron-beam lithography (EBL; Vistec 5000 + ES). Prior to exposure, the flakes were spin-coated with a ~ 350 nm thick layer of polymethyl methacrylate (PMMA) 950 K resist. After development (60 s) and an isopropanol (IPA) rinse, patterned holes were transferred into the h^10^BN layer via reactive ion etching (RIE; Etchlab 200). Residual PMMA was removed by soaking in hot acetone at 80 °C for 30 min, followed by a gentle IPA rinse and N₂ drying.

Following nanopatterning, the *h*¹⁰BN flakes were transferred onto (100)-cut calcite substrates using a PMMA-assisted wet transfer method. A PMMA 950 K layer (~350 nm) was spin-coated onto the SiO₂/Si substrate and baked. The PMMA/h^10^BN stack was released by etching the underlying SiO_2_ in 1 M KOH and then scooped onto the calcite substrate. After transfer, the PMMA support layer was removed by soaking in acetone at 80 °C for >1 h. Residual contaminants were further cleaned in PG remover (60 min) and warm acetone (30 min), followed by a final IPA rinse and N₂ drying.

### Cryo-SNOM imaging

In the low-temperature near-field imaging experiments, we employed a home-built cryo-SNOM system, which is equipped with both AFM functions and s-SNOM imaging capabilities. This setup utilizes a continuous wave quantum cascade laser (MIRcat QCL) as the illumination source. The laser light is focused by a parabolic mirror (PM) to illuminate the probe tip. In all experiments, we used Arrow™ NCPt probes, which feature micro-engineered cantilevers with a tip quality factor of ∼1000. The tip oscillates vertically with an amplitude of about 100 nm at a frequency of Ω ≈ 270 kHz. All cryo-SNOM images presented in this work were obtained by demodulating the total backscattered signal using a lock-in amplifier at the third-order harmonic of the tip-tapping frequency.

### COMSOL simulations

COMSOL Multiphysics version 6.1 was used to simulate the propagation behaviors of polaritons in flat and wrinkled vdW nanosheets. Reciprocal space maps were generated by performing 2D Fourier transformations of the real-space electric field profiles using the software Gwyddion. Detailed simulation parameters and procedures for each Figure can be found in the Supplementary Information, Section 1.

## Supplementary information

Supplementary Information

Supplementary Dataset 1

Transparent Peer Review file

## Data Availability

The Source Data underlying the figures of this study are available with the paper. All raw data generated during the current study are available from the corresponding authors upon request.
