# Supplementary material for: Nano-wrinkle waveguides for programmable polariton channeling
Source: Nat Commun. 2026 Sep 17;17:10905. doi: 10.1038/s41467-026-77593-y (PMC13677736; doi:10.1038/s41467-026-77593-y)
Supplement: Supplementary file 1 [file 41467_2026_77593_MOESM1_ESM.pdf]

# Supplementary information

## Nano-wrinkle waveguides for programmable polariton channeling

*Liyan Wang*[1,†], *Bei Yang*[2,†], *Tian Sun*[3,†], *Cheng Luo*[1], *Yuchuan Xiao*[1], *Qizhi Yan*[3], *Weikang Lu*[4], *Ziyi Xu*[4], *Zhenghua An*[4], *Wei Lu*[5], *Xingxing Jiang*[6], *Debo Hu*[1,*], *Andrea Alù*[7,8,*], *Peining Li*[2,3,*], *Qing Dai*[2,*]

[1]CAS Key Laboratory of Standardization and Measurement for Nanotechnology, National Center for Nanoscience and Technology, Beijing, China.
[2]School of Materials Science and Engineering, Shanghai Jiao Tong University, Shanghai, China.
[3]School of Optical and Electronic Information, Huazhong University of Science and Technology, Wuhan, China.
[4]Department of Physics, Fudan University, Shanghai, China.
[5]School of Physical Science and Technology, ShanghaiTech University, Shanghai, China.
[6]Technical Institute of Physics and Chemistry, Chinese Academy of Sciences, Beijing, China.
[7]Advanced Science Research Center, City University of New York, New York, NY, USA.
[8]Graduate Center, City University of New York, New York, NY, USA.
[†]These authors contributed equally: Liyan Wang, Bei Yang, Tian Sun.
*Corresponding authors: Debo Hu, hudb@nanoctr.cn; Andrea Alù, aalu@gc.cuny.edu; Peining Li, lipn@hust.edu.cn; Qing Dai, daiqing@sjtu.edu.cn.

## Section 1 More details of numerical simulations

### 1.1 The dielectric function of calcite and $h^{10}$BN

Calcite belongs to the trigonal crystal system, and its dielectric tensor of (100) crystal plane is a diagonal matrix in the principal axis coordinate system:

$$\varepsilon_{calcite} = \begin{bmatrix} \varepsilon_o & 0 & 0 \\ 0 & \varepsilon_e & 0 \\ 0 & 0 & \varepsilon_0 \end{bmatrix}$$

$\varepsilon_o$is from the component in the direction perpendicular to the optic axis; $\varepsilon_e$ is the dielectric constant along the optical axis direction.

To calculate the uniaxial dielectric tensor of calcite, we used the Lorentz oscillator models. In this model, one Lorentz oscillator is used in the $\varepsilon_o$ direction, while two oscillators are used in the $\varepsilon_e$ direction:

$$\varepsilon_{\mathrm{e}} = \varepsilon_{\infty,1}\left(1 + \frac{\omega_{\mathrm{LO},1}^2 - \omega_{\mathrm{TO},1}^2}{\omega_{\mathrm{TO},1}^2 - \omega^2 - \mathrm{i}\omega\Gamma_1} + \frac{\omega_{\mathrm{LO},2}^2 - \omega_{\mathrm{TO},2}^2}{\omega_{\mathrm{TO},2}^2 - \omega^2 - \mathrm{i}\omega\Gamma_2}\right)$$

$$\varepsilon_{\mathrm{o}} = \varepsilon_{\infty,3}\left(1 + \frac{\omega_{\mathrm{LO},3}^2 - \omega_{\mathrm{TO},3}^2}{\omega_{\mathrm{TO},3}^2 - \omega^2 - \mathrm{i}\omega\Gamma_3}\right)$$

$\varepsilon_\infty$ is the high-frequency permittivity. $\omega_{\mathrm{TO}}$ and $\omega_{\mathrm{LO}}$ are the TO and LO phonon frequencies, respectively. $\Gamma$ is the damping constant. For $\varepsilon_{\mathrm{e}}$, we took $\varepsilon_{\infty,1}$ = 2.4, $\omega_{\mathrm{TO},1}$ = 712 cm$^{-1}$, $\omega_{\mathrm{LO},1}$ = 715 cm$^{-1}$, $\Gamma_1$ = 5 cm$^{-1}$; $\omega_{\mathrm{TO},2}$ = 1,410 cm$^{-1}$, $\omega_{\mathrm{LO},2}$ = 1550 cm$^{-1}$, $\Gamma_2$ = 10 cm$^{-1}$. For $\varepsilon_{\mathrm{o}}$, we took $\varepsilon_{\infty,3}$ = 2.7, $\omega_{\mathrm{TO},3}$ = 871 cm$^{-1}$, $\omega_{\mathrm{LO},3}$ = 890 cm$^{-1}$, $\Gamma_3$ = 3 cm$^{-1}$. The parameters above are from ref. 1 of Supplementary Information.[1]

$h^{10}$BN belongs to the hexagonal crystal system, and the dielectric tensor is divided into in-plane ($\varepsilon_\parallel$) and out of plane ($\varepsilon_\perp$) components:

$$\varepsilon_{h\mathrm{BN}} = \begin{bmatrix} \varepsilon_\parallel & 0 & 0 \\ 0 & \varepsilon_\parallel & 0 \\ 0 & 0 & \varepsilon_\perp \end{bmatrix}$$

The dielectric constant of $h^{10}$BN exhibits strong phonon polariton resonance characteristics, requiring the use of a double oscillator Lorentz model:

$$\varepsilon_{\parallel,\perp} = \varepsilon_{\infty,\parallel,\perp}\left(1 + \frac{\omega_{\mathrm{LO}}^2 - \omega_{\mathrm{TO}}^2}{\omega_{\mathrm{TO}}^2 - \omega^2 - \mathrm{i}\omega\Gamma}\right)$$

For $\varepsilon_\parallel$, we took $\varepsilon_{\infty,\parallel}$ = 5.1, $\omega_{\mathrm{LO},1}$ = 1650 cm$^{-1}$, $\omega_{\mathrm{TO},1}$ = 1394.5 cm$^{-1}$, $\Gamma_1$ = 1.8 cm$^{-1}$; for $\varepsilon_\perp$, we took $\varepsilon_{\infty,\perp}$ = 2.5, $\omega_{\mathrm{LO},2}$ = 845 cm$^{-1}$, $\omega_{\mathrm{TO},2}$ = 785 cm$^{-1}$, $\Gamma_2$ = 1 cm$^{-1}$. The parameters above are from ref. 2 of Supplementary Information.[2]

After the formation of wrinkles in *h*BN, the microstructure of the material changes, resulting in anisotropy of the dielectric function in the *xy* direction:

$$\varepsilon_{(x,y)} = \begin{bmatrix} \varepsilon_{xx} & \varepsilon_{xy} & 0 \\ \varepsilon_{xy} & \varepsilon_{yy} & 0 \\ 0 & 0 & \varepsilon_{zz} \end{bmatrix}$$

As shown in Fig. 1e, the cross-section profile of the wrinkle in Fig. 1e obtained by AFM at 120 K fits well with the sinusoidal model:

$$z(y) = h/2\{1 + \cos[2\pi(y - y_c)/w\}$$

In the wrinkled region (x <$|w/2|$), the angle $\theta$ between the wrinkle at point *x* and the *x*-axis in the *xz* plane can be expressed as:

$$\theta = \arctan(-\frac{h\pi}{w}\sin\left(\frac{2\pi x}{w}\right))$$

Then, both diagonal and coupling terms can be generated by modulation functions:

$$\varepsilon_{xx} = \varepsilon_{\parallel} \times cos^2\theta + \varepsilon_{\perp} \times sin^2\theta,$$
$$\varepsilon_{xy} = \varepsilon_{\parallel} \times cos\theta sin\theta + \varepsilon_{\perp} \times cos\theta sin\theta,$$
$$\varepsilon_{yy} = \varepsilon_{\perp} \times cos^2\theta + \varepsilon_{\parallel} \times sin^2\theta,$$
$$\varepsilon_{zz} = \varepsilon_{\perp}.$$

Based on the variable-temperature Raman data (Fig. 1b), the accumulated strain is fully released after wrinkle formation. Therefore, for the simulations and cryo-SNOM experiments conducted at 90 K (post-buckling regime), only the temperature dependence of the dielectric function is considered. This dependence is incorporated by correcting the frequency of the dominant $E_{2g}$ phonon using the experimentally measured coefficient $k_{post}$=−0.01892 cm$^{-1}$/K, which shifts the $E_{2g}$ frequency to 1399.4 cm$^{-1}$ at 90 K. The $E_{2g}$ mode is chosen because it is the dominant Raman-active phonon in $h^{10}$BN and its frequency shift directly reflects the intrinsic thermal response in the strain-released state. At the operational frequency of 1460 cm$^{-1}$, the dielectric response is governed primarily by the $E_{2g}$ phonon, while contributions from other modes (e.g., the $A_{2u}$ phonon near 785 cm$^{-1}$) are negligible due to the large spectral separation.

**1.2 Simulations Details for Fig. 2, and Supplementary Fig. 5-9**

For the simulations shown in Fig. 2, and Supplementary Fig. 5-9, an electric dipole source was used to excite polaritons in flat and wrinkled $h^{10}$BN nanosheets, with or without a (100) calcite substrate. The uniaxial permittivity of calcite used for the simulation is $\varepsilon_o$=2.3387+1.e$^{-4}$i and $\varepsilon_e$=-5.0243+0.7852i at $\omega$=1460 cm$^{-1}$. The calcite optical axis (OA) is oriented at an angle of $\theta$ =23.3° relative to the (100) surface. The uniaxial permittivity of $h^{10}$BN used for the simulation is $\varepsilon_{\parallel}$=2.3387+1.5545e$^{-4}$i and $\varepsilon_{\perp}$=-17.386+0.34103i at $\omega$=1460 cm$^{-1}$. The dipole source is located 100 nm above the $h^{10}$BN surface. The near-field distribution was captured from a plane

at the $h^{10}$BN surface. The Fourier transforms (FT) shown in Fig. 2, Supplementary Fig. 5, 6, and 9 were obtained using the software Gwyddion.

For the mode analysis of the $M_g$ mode shown in Fig. 2e and Supplementary Fig. 5e, we used the two-dimensional (2D) mode solver in COMSOL to calculate the modal profile of the $M_g$ mode with the wavevector $k$=24.58$k_0$ (with calcite substrate) and $k$=24.65$k_0$ (without calcite substrate), respectively. The obtained modal profile is shown in Fig. 2e and Supplementary Fig. 5e. By combining two COMSOL modules, the boundary mode analysis and the frequency-domain study, we calculated the polariton mode's electric field distribution $E_x$ along the propagation direction (cross section in the $y$-$z$ plane). This approach allows for a detailed understanding of the polariton behavior in the presence and absence of a calcite substrate.

**1.3 Simulations Details for Fig. 4**

To quantify the heat generation and transport mediated by phonon polaritons in *h*BN wrinkles and related films, we developed a three-dimensional numerical model using the commercial software COMSOL Multiphysics. This model couples the electromagnetic (EM) and heat transfer modules to capture the multiphysics interactions within the system. The transient temperature distributions in both the *h*BN nanostructure and the underlying calcite substrate under irradiation are governed by the general heat transfer equations:[3]

$$\rho_{hBN} C_{p,hBN} \frac{\partial T_{hBN}}{\partial t} = \nabla \cdot [\kappa_{hBN} \nabla T_{hBN}] - G(T_{hBN} - T_s) + Q_{sur} + Q_{hBN}$$

$$\rho_s C_{p,s} \frac{\partial T_s}{\partial t} = \nabla \cdot [\kappa_s \nabla T_s] - G(T_s - T_{hBN}) + Q_{sur}$$

where $\rho$, $C_p$, and $\kappa$ represent the density, specific heat capacity, and thermal conductivity of the respective material. The interfacial thermal conductance $G$ accounts for heat exchange through conduction at the interface between *h*BN and the calcite substrate. The subscripts "*h*BN" and "s" denote the *h*BN nanostructure and the substrate, respectively. The term $Q_{sur}$ represents radiative heat exchange between the heterostructure and the surrounding vacuum, while $Q_{hBN}$ captures internal heat generation within the *h*BN, primarily arising from polarized current power dissipation due to phonon polaritons excitation.

According to Poynting's theorem, the volumetric heat generation within the *h*BN can be expressed as:

$$Q_{hBN} = \iiint q \, \mathrm{d}V$$

where $q = \frac{1}{2}\mathrm{Re}(\mathbf{J} \cdot \mathbf{E}^*)$ is the EM power loss density, i.e. heat power density, $\mathbf{E}$ is the electric field, and $\mathbf{J} = \sigma\mathbf{E}$ is the current density determined by the local electric field and electrical

conductivity $\sigma$. Both **E** and **J** are computed numerically using the finite element method under excitation at a specific incident frequency, which induces dipolar phonon polariton modes in the *h*BN structure.[4]

The thermophysical properties of *h*BN used in the simulations are based on established literature values. Notably, *h*BN exhibits strong thermal anisotropy, with significantly higher in-plane thermal conductivity than out-of-plane. For suspended *h*BN wrinkles, we assume in-plane and out-of-plane thermal conductivities of 800 and 4 W/(m·K), respectively. For substrate-supported *h*BN, these values are reduced to 400 and 2 W/(m·K). The interfacial thermal conductance between *h*BN and calcite is set to be 70 MW/($m^2$·K). To excite phonon polaritons in *h*BN, a vertically oriented electric dipole is introduced, with a dipole moment of 1 nA·m along the *z*-axis and an excitation frequency of 1460 $cm^{-1}$.

**1.4 Simulations Details for Supplementary Fig. 14**

The optical response of graphene is governed by its surface conductivity $\sigma(\omega, E_F, \tau, T)$, which comprises both intraband and interband contributions. Under the conditions $k_B\mathrm{T} \ll \hbar\omega$ and $k_B\mathrm{T} \ll |E_F|$, the total conductivity can be expressed as[5]:

$$\sigma = \sigma_{\mathrm{intra}} + \sigma_{\mathrm{inter}},$$

where the intraband and interband components are given by:

$$\sigma_{\mathrm{intra}} = \frac{e^2}{\pi\hbar^2} \cdot \frac{iE_F}{\omega + i\tau^{-1}},\ \sigma_{\mathrm{inter}} = i\frac{e^2}{4\pi\hbar}\ln\left(\frac{2|E_F| - \hbar(\omega + i\tau^{-1})}{2|E_F| + \hbar(\omega + i\tau^{-1})}\right).$$

Here, $e$ is the electron charge, $\hbar$ is the reduced Planck constant, $E_F$ is the Fermi energy, $\tau$ is the carrier relaxation time, and $\omega$ is the angular frequency of the incident light. The relaxation time is given by $\tau=\mu E_F/(e v_F^2)$, where $v_F \approx c/300$ is the Fermi velocity and $\mu$ is the carrier mobility.

In the electromagnetic simulations performed using COMSOL Multiphysics, the graphene layer was modeled as a thin film with a finite thickness $t_g$=0.34 nm and an effective anisotropic dielectric function. The in-plane component is defined as:

$$\varepsilon_{\parallel} = 1 + i\frac{4\pi\sigma}{\omega t_{\mathrm{g}}},$$

while the out-of-plane component is set to $\varepsilon_{\perp}$=1. For the near-field image simulations in Supplementary Fig. 14, the graphene was modeled as a transition interface with the above dielectric properties.

**Section 2: Supplementary Figures**

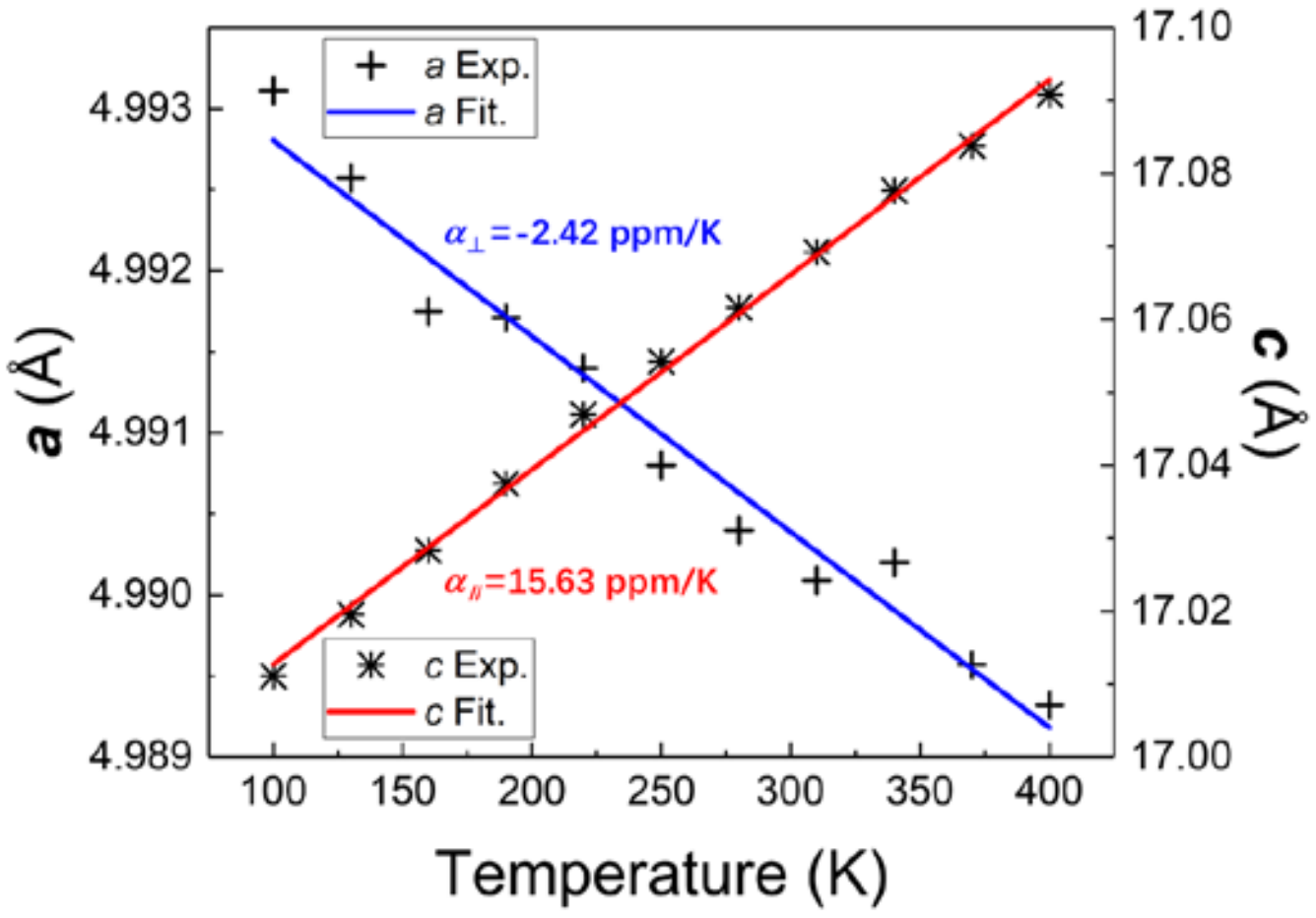


**Supplementary Fig. 1 |** Temperature-dependent cell parameters and fitted linear TECs of calcite.

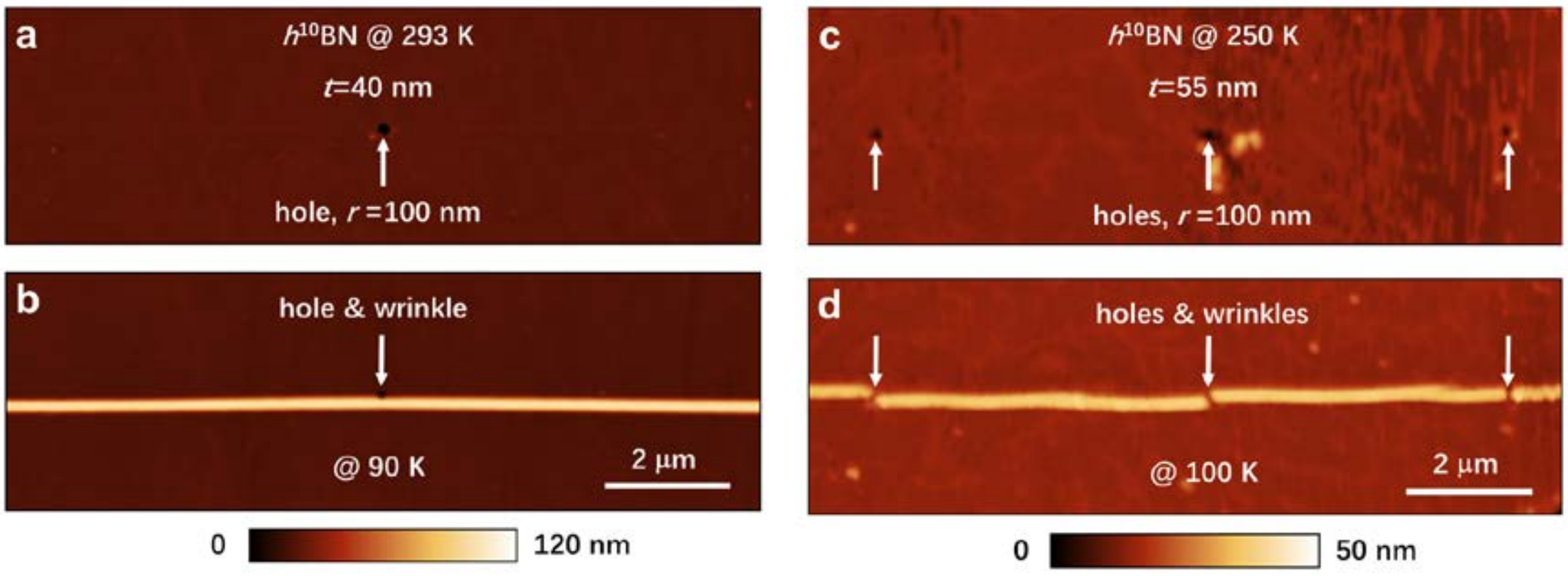


**Supplementary Fig. 2 | Pinning *h*$^{10}$BN nano-wrinkles with nano-holes. a**, A 40-nm-thick *h*$^{10}$BN nanosheet with a 100-nm-radius hole. **b**, A wrinkle pinned down by the single nano-hole in (**a**) at 90 K. **c**, A 55-nm-thick *h*$^{10}$BN nanosheet with multiple holes. **d**, Wrinkle segments pinned down by the hole-array in (**c**) at 100 K.

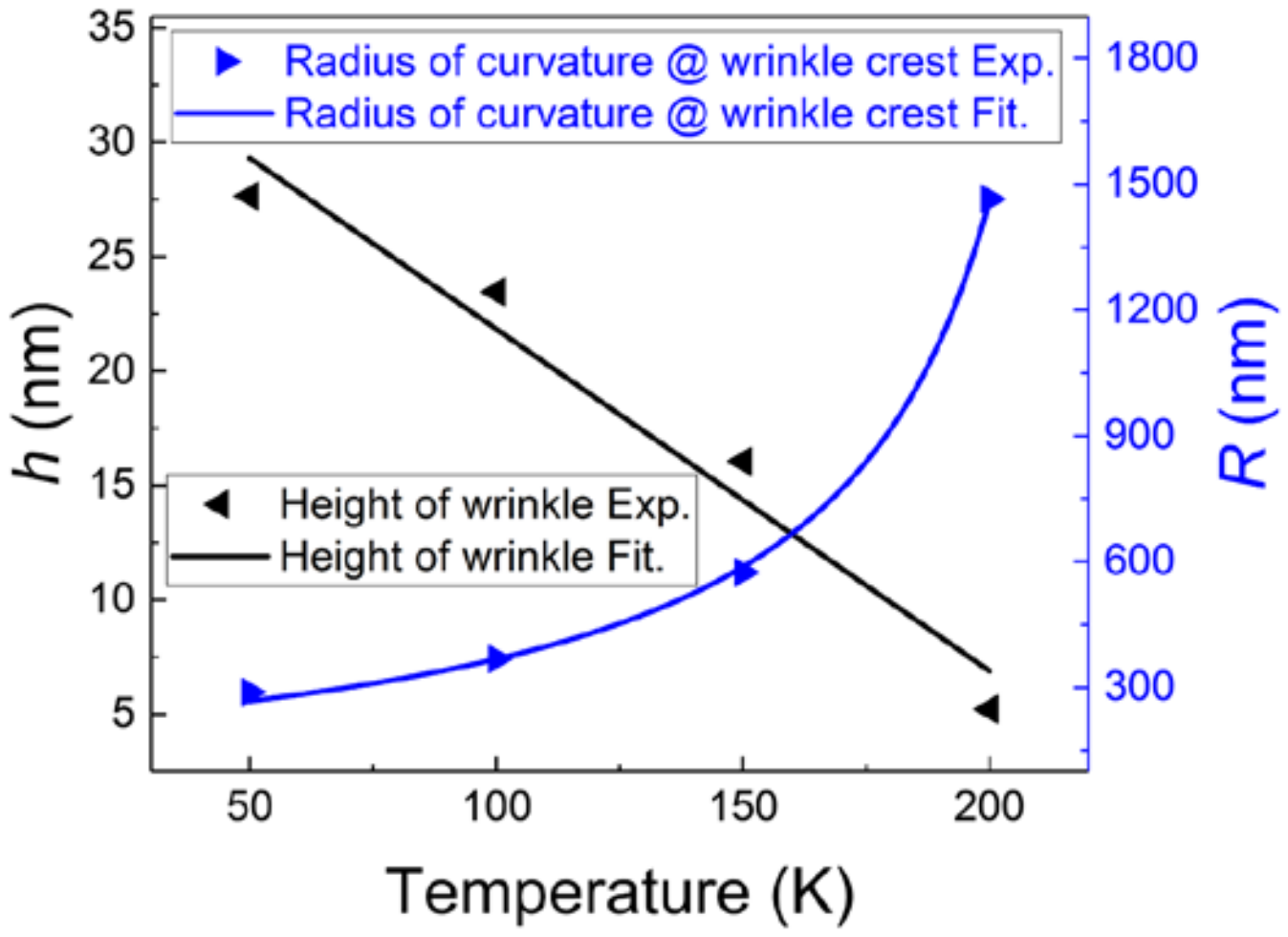


**Supplementary Fig. 3 |** Temperature dependence of heights and curvature radii for the wrinkle segments in Fig. S2c.

**Controllable and Repeatable Geometry**: The wrinkle dimensions (height and width) are tunable and repeatable under controlled cycling conditions. Multiple cooling-heating cycles are conducted on a graphite/calcite heterostructure, and the morphology of the wrinkled region is characterized, as summarized in Supplementary Fig. 4a (AFM topographies) and 4b (statistical analysis of wrinkle dimensions).

Cycle-1: Cooling from room temperature to 88.5 K, warming to 210 K, and re-cooling to 88.8 K.

Cycle-2: Involved a larger thermal swing. The sample was first pre-heated to 473 K, then cooled to 88.5 K, warmed to 210 K, and finally cooled to 87.3 K. The nearly twofold greater temperature drop compared to Cycle-1 resulted in a significantly larger wrinkle, confirming that dimensions can be amplified by increasing the thermal stress.

Cycle-3: Following Cycle-2, the sample was cooled again to 90 K. Due to the partial delamination and reduced interfacial adhesion from the previous large-strain cycle, the newly formed wrinkle was substantially larger than the one in Cycle-1.

Cycle-4: The sample was again heated to 473 K and cooled to 90 K. The resulting wrinkle dimensions were closely matched to those in Cycle-2 (~5% variation), demonstrating that consistent thermal variation leads to reproducible wrinkle morphology.

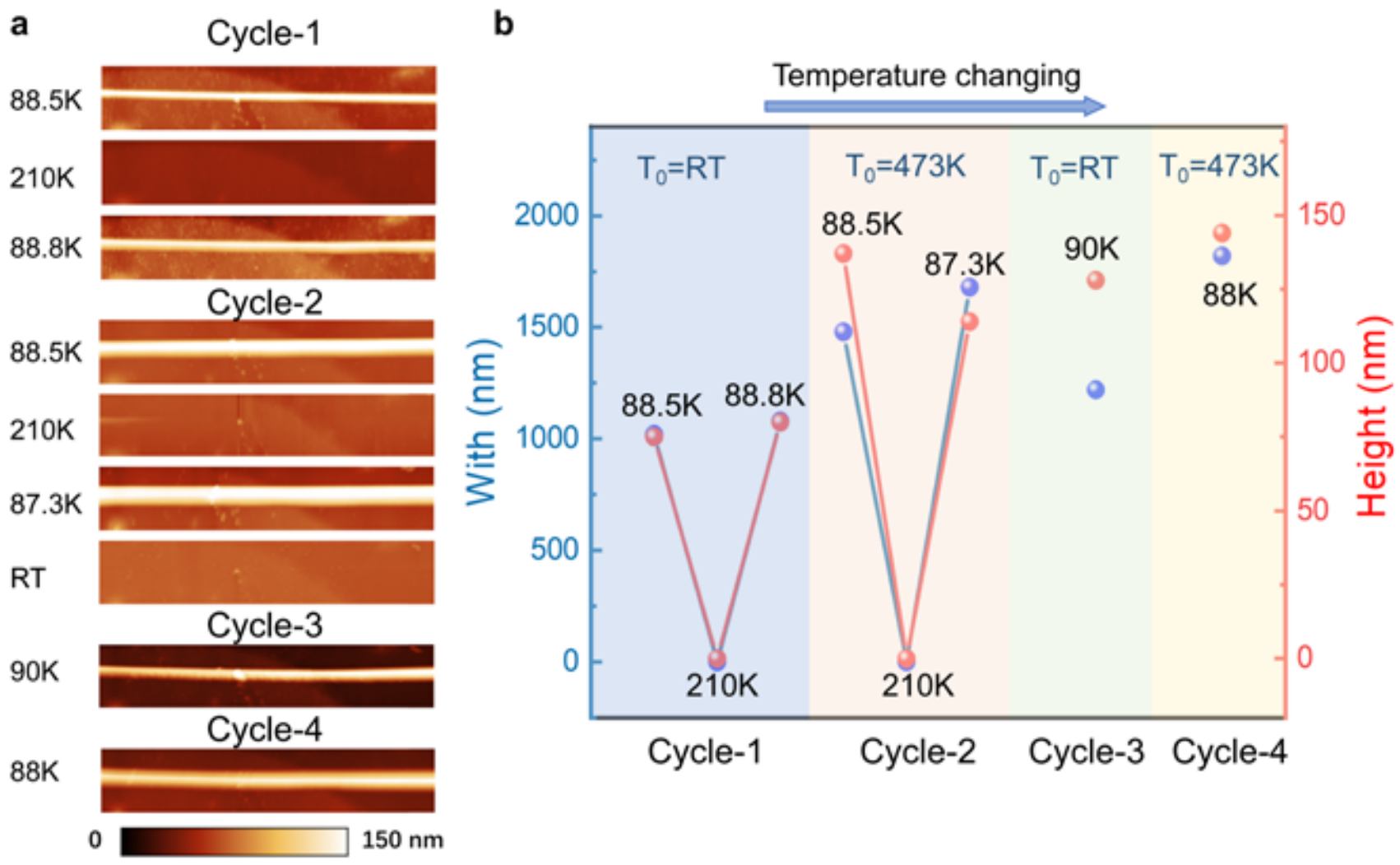


**Supplementary Fig. 4 | Reversibility and tunability of nano-wrinkles under cyclic thermal loading. a**, AFM topography images of a wrinkled graphite/calcite heterostructure across four thermal cycles (Cycle-1 to Cycle-4), demonstrating deterministic wrinkle re-formation at nearly identical locations after repeated cooling below and warming above the critical temperature. **b,** Statistical analysis of wrinkle dimensions (height and width) extracted from the cycles in a. The height and width are tunable and repeatable under controlled thermal conditions: a larger temperature drop (Cycle-2, pre-heated to 473 K before cooling) produces a significantly larger wrinkle compared to a moderate drop (Cycle-1, RT cooling to 88.5K). Following a large-strain cycle, partial delamination can lead to increased dimensions (Cycle-3, RT cooling to 90K). When the same thermal swing is reapplied (Cycle-4), the wrinkle dimensions closely match those of Cycle-2 (within ~5% variation), confirming that reproducible morphology is achieved with a consistent thermal history.

The confinement and guiding effects of a nano-wrinkle for $h^{10}$BN polaritons can be demonstrated by comparing their propagating behaviors in a flat and a wrinkled $h^{10}$BN nanosheets, both assumed to be freestanding to eliminate ambiguities resulting from substrate. As shown in Supplementary Fig. 5a, the dipole launched polaritons ($\omega$=1460 cm$^{-1}$) spread isotropically at the surface of a 60-nm-thick $h^{10}$BN flake. The two-dimensional (2D) Fourier transform of (a) is shown in Supplementary Fig. 5b, which resolves the mixture of waves in (a) into three discrete polariton modes ($M_1$, $M_2$, and $M_3$) with increasing in-plane wavevectors (real parts, $k_{M1}$=2.13$k_0$, $k_{M2}$=23.27$k_0$, $k_{M3}$=44.42$k_0$) in $k$-space. The simulation results of a 400-nm-wide and 100-nm-high wrinkle shown in Supplementary Fig. 5c-e confirm: the presence of wrinkle breaks the in-plane isotropy of $h^{10}$BN nanosheet and a new polariton mode ($M_g$) propagating along the wrinkle with shorter wavelength than $M_2$ emerges (Supplementary Fig.

5c); the 2D Fourier transform of (c) shown in Supplementary Fig. 5d indicates the $M_g$ mode has a larger wavevector ($k_{Mg}$=24.65$k_0$) than $M_2$; the calculated cross-sectional mode profile shown in Supplementary Fig. 5e manifests that $M_g$ mode is localized in the vicinity of the wrinkle. Dispersion of $M_g$ at 1460 cm$^{-1}$ against increasing wrinkle height *h* (decreasing radius of curvature *R*) and a fixed wrinkle width *w*=400 nm has been summarized in Supplementary Fig. 5f with *h* ranging from zero to 120 nm (*R* from infinity to 68 nm). As a general rule, both the mode confinement factor (real part of the effective index of refraction $N_{eff}$) and propagation loss (imaginary part of $N_{eff}$) increase with the height of the wrinkle *h*.

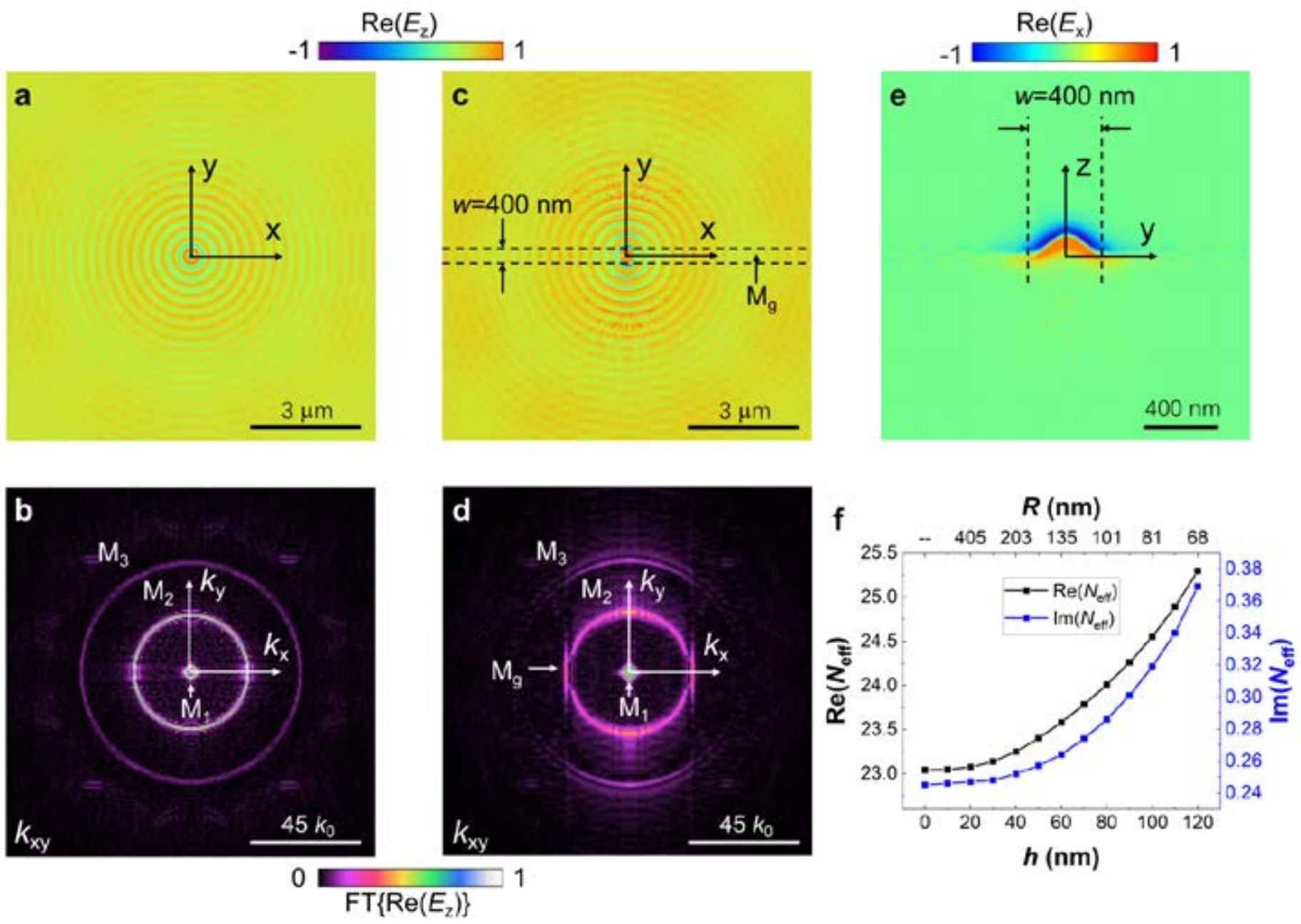


**Supplementary Fig. 5 | Confinement and guiding of a new phonon polariton mode ($M_g$) by the geometric potential well associated with a wrinkled freestanding $h^{10}$BN nanosheet.** **a**, A simulated snapshot of the polariton wavefronts launched by a *z*-oriented dipole located 100-nm above the surface of a 60-nm thick $h^{10}$BN flake, the excitation frequency is 1460 cm$^{-1}$ and the out-of-plane component of the optical field $E_z$ is shown. **b**, Fourier transform of (**a**), indicating at least three modes ($M_1$, $M_2$, and $M_3$ with increasing in-plane wavevectors $k_{xy}$) have been excited by the dipole. **c**, Simulated wavefronts at the surface of a wrinkled $h^{10}$BN nanosheet, the width of the wrinkle *w* is 400 nm and the height *h* is 100 nm, the dipole locates 100-nm above the crest of the wrinkle. A new phonon polariton mode ($M_g$) propagating in the +/-*x* direction with shorter wavelength than $M_2$ can be identified. **d**, Fourier transform of (**c**), indicating the $M_2$ mode in the *x* direction has been transformed into $M_g$ mode with larger wavevector by the wrinkle induced geometric potential well. **e**, Cross-sectional mode profile of $M_g$ in (**c**), the in-plane component of the optical field $E_x$ is shown. **f**, Dispersion of the $M_g$ mode

at 1460 cm$^{-1}$ against increasing wrinkle height *h* (decreasing radius of curvature *R*) and a fixed wrinkle width *w*=400 nm. (**a**), (**c**), and (**e**) share the same false color ruler, (**b**) and (**d**) share another.

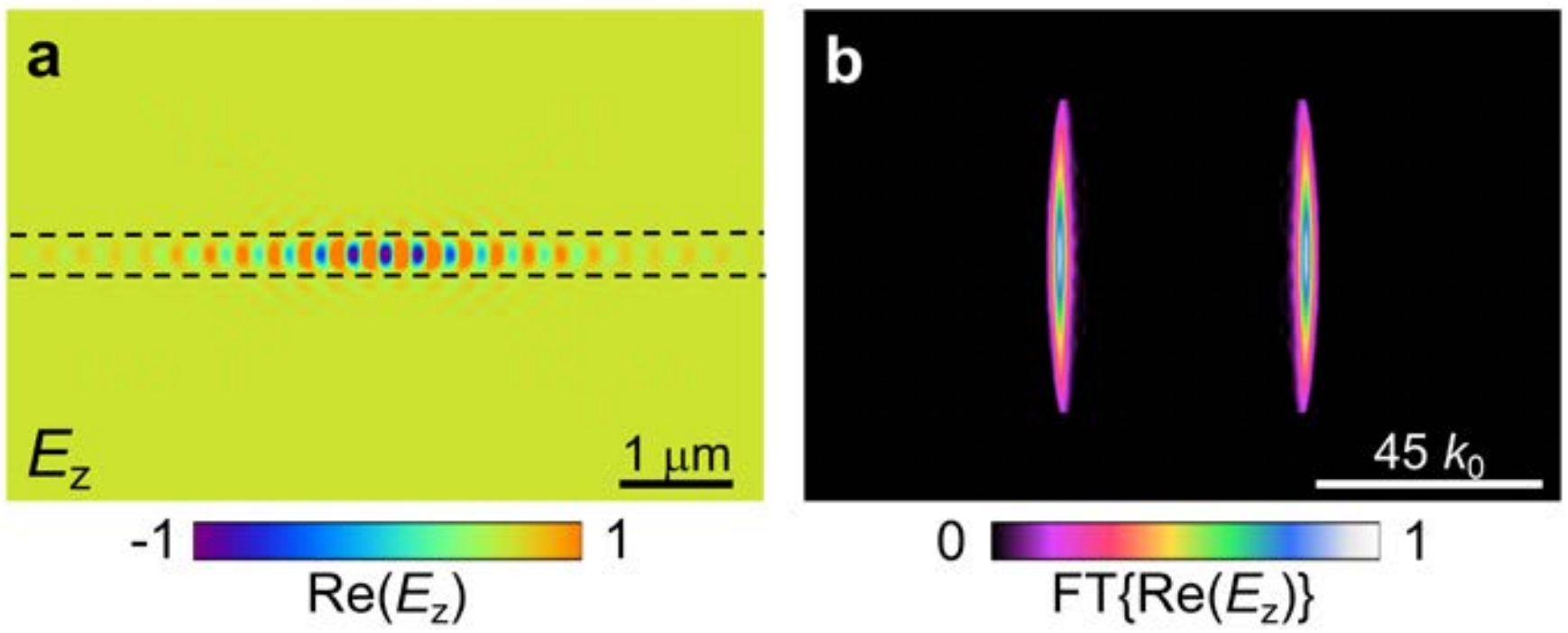


**Supplementary Fig. 6 | Isolation of the wrinkle-guided polariton mode via spatial frequency filtering. a**, Filtered real-space image of the simulated electric field after applying a spatial frequency filter to the Fourier transform in Fig. 2d. **b**, Corresponding filtered momentum-space map of the simulation. The spatial-frequency filtering was performed using the 2D FFT filtering function in the near-field data-analysis software *Gwyddion*.

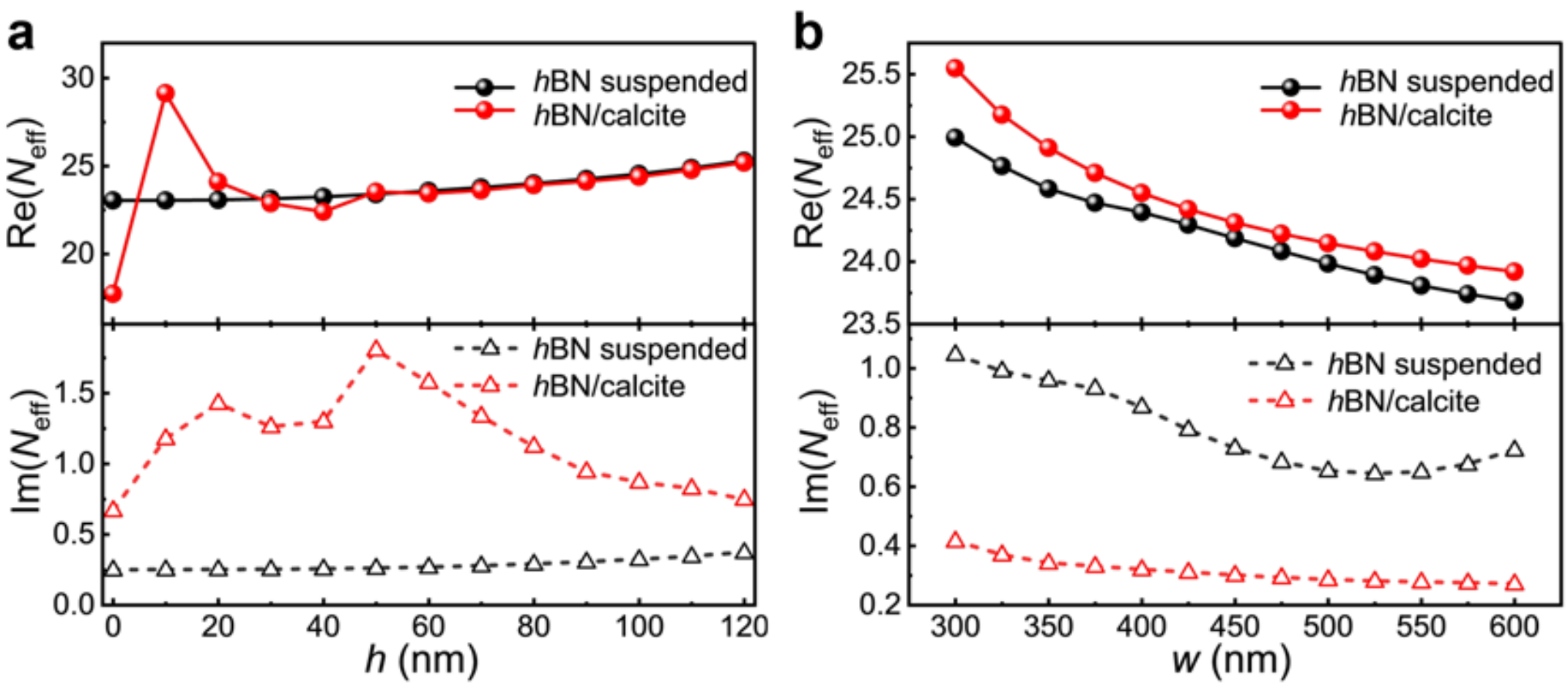


**Supplementary Fig. 7 | Evolution of the wrinkle-guided $M_g$ mode with wrinkle geometry. a**, Real (solid circles) and imaginary (open triangles) parts of the effective refractive index $N_{eff}$ of the $M_g$ mode as a function of wrinkle height *h* for suspended *h*BN (black) and *h*BN/calcite (red) structures. **b**, Real (solid circles) and imaginary (open triangles) parts of $N_{eff}$ of the $M_g$ mode as a function of wrinkle width *w* for suspended *h*BN (black) and *h*BN/calcite (red) structures.

The confinement ratio is defined as:

$$\eta = \frac{P_{\text{wrinkle}}}{P_{\text{total}}} \times 100\% = \frac{\iint_{\text{wrinkle}} S_x(y,z)dydz}{\iint_{\text{A}} S_x(y,z)dydz} \times 100\%$$

where $S_x$ is the time-averaged Poynting vector component along the propagation direction, $P_{\text{wrinkle}}$ is the power flowing within the wrinkle region (as indicated by the rectangular region in Supplementary Fig. 8b), and $P_{\text{total}}$ is the total power crossing the entire transverse section.

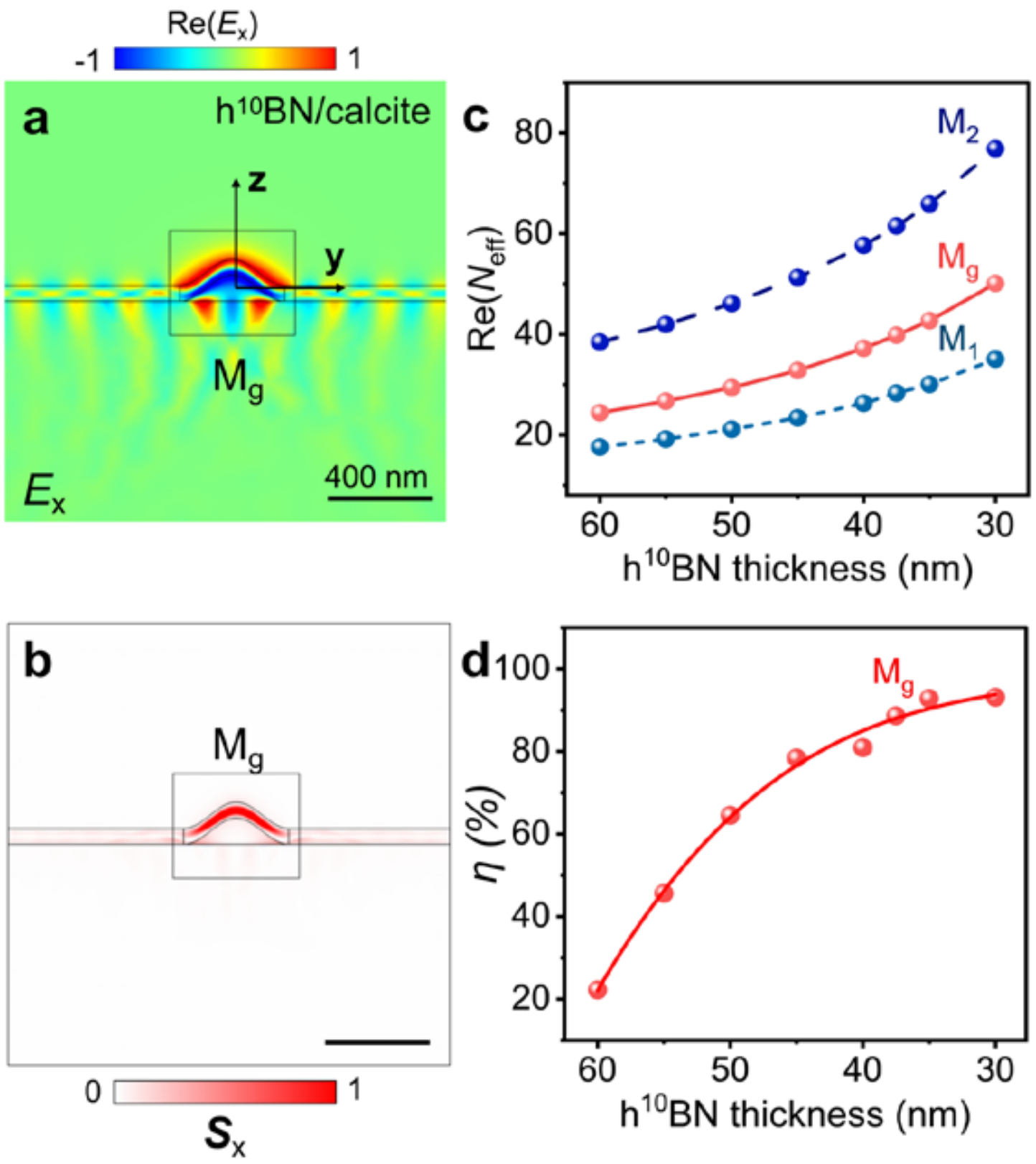


**Supplementary Fig. 8 | Power-flow confinement and thickness-dependent tunability of wrinkle-guided polariton modes in *h*$^{10}$BN. a**, Cross-sectional mode profile of $M_g$ in Fig. 2, the component of the optical field $E_x$ is shown. **b**, Cross-sectional $S_x$ distribution of the $M_g$ mode. **c**, Real part of the effective refractive index Re($N_{eff}$) of the $M_g$ wrinkle-guided mode as a function of *h*$^{10}$BN thickness. **d**, Confinement ratio $\eta_{Mg}$ as a function of *h*$^{10}$BN thickness.

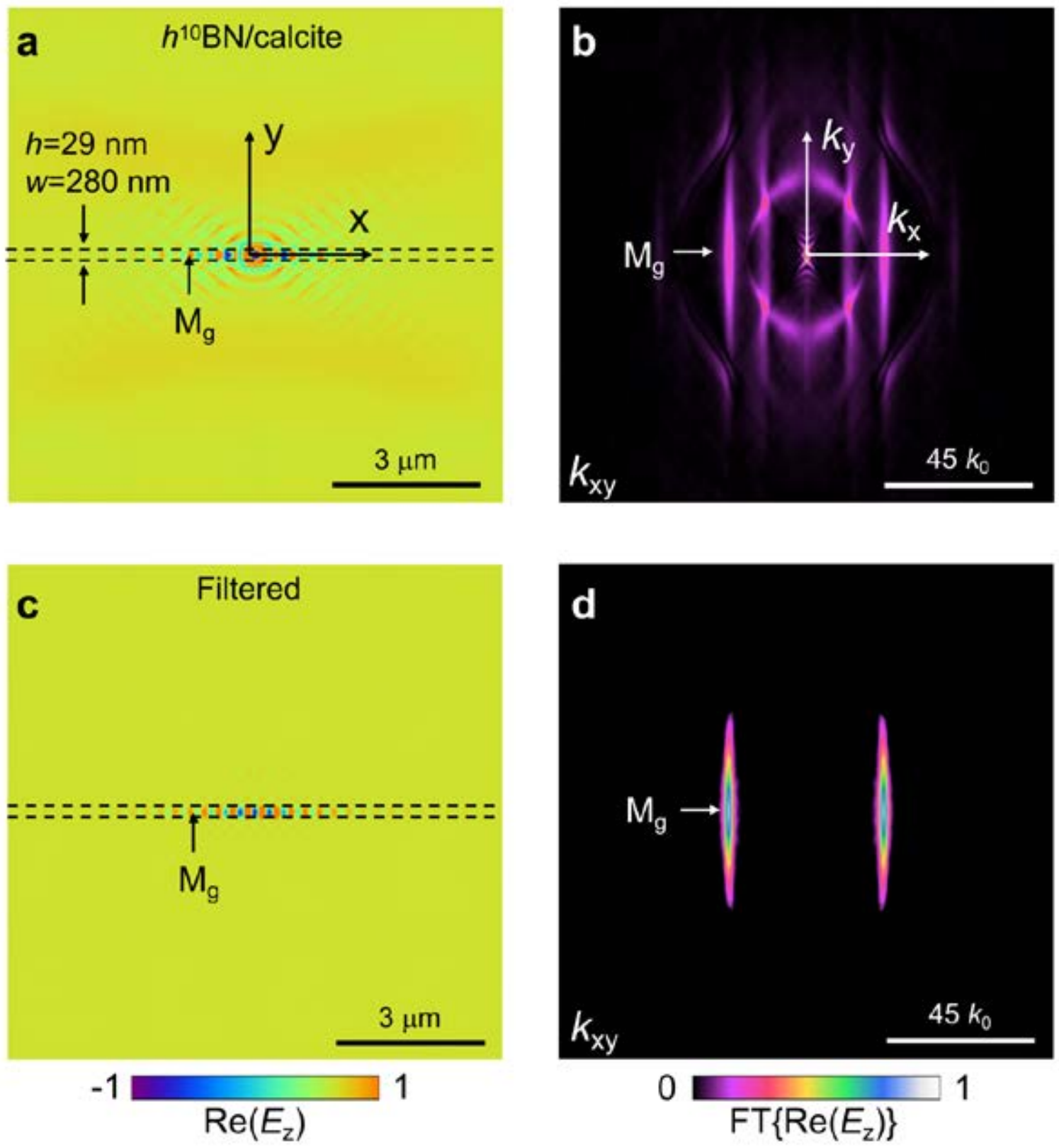


**Supplementary Fig. 9 | Simulated polariton propagation corresponding to the Fig. 3d wrinkled $h^{10}$BN nanosheet. a**, The $h^{10}$BN nanosheet on calcite substrate. Wrinkle dimensions: the width of the wrinkle $w$ is 280 nm and the height $h$ is 29 nm. Simulated $M_g$ mode wavevector $k_{Mgx}$=23.49$k_0$. **b** Fourier transform of (a): $M_g$ mode marked by arrow. **c**, Filtered real-space image of the simulated electric field after applying a spatial frequency filter to the Fourier transform in (b). **d**, Corresponding filtered momentum-space map of (c).

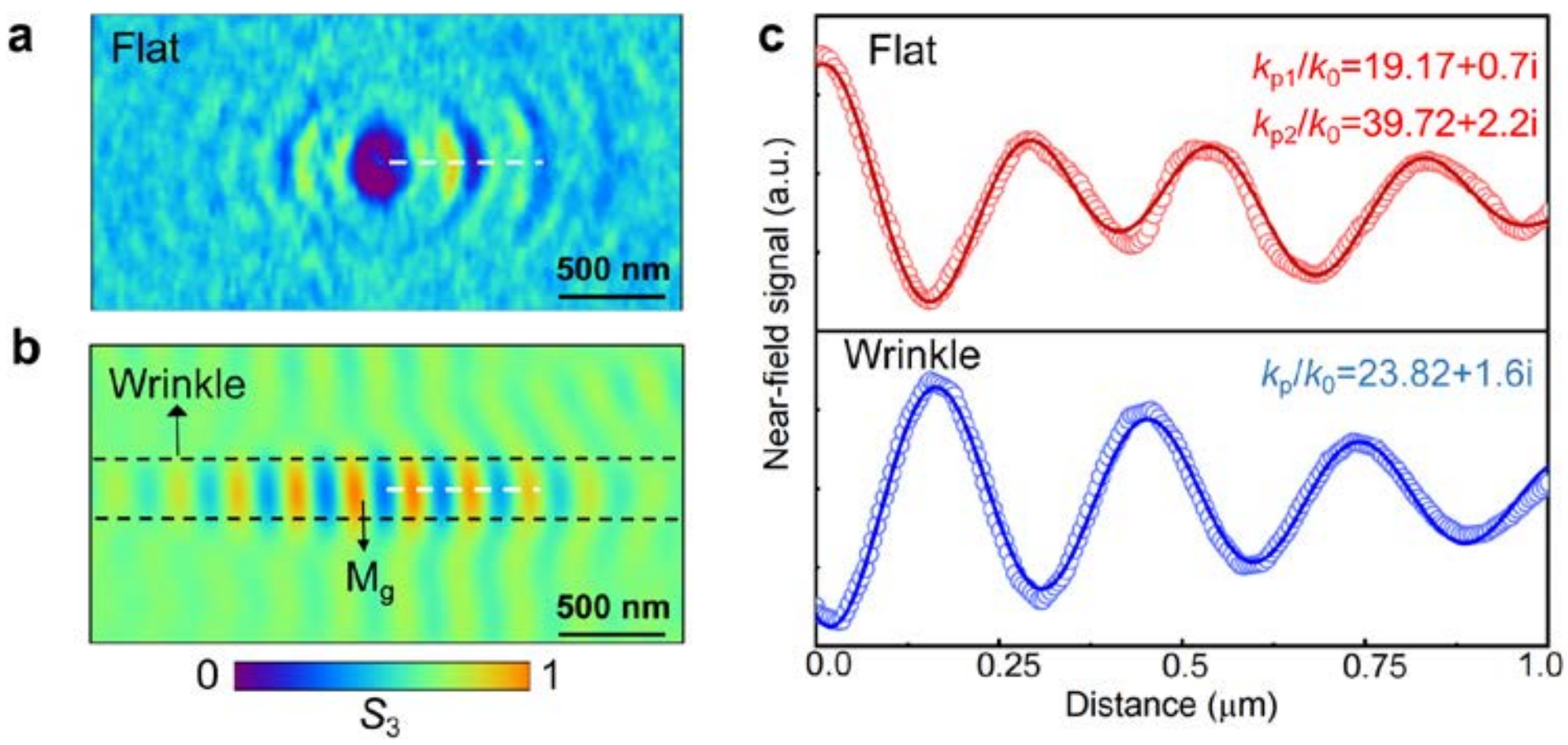


**Supplementary Fig. 10 |** Isolation of the wrinkle-guided polariton mode from experimental near-field data. **a**, Near-field amplitude image of phonon polaritons on a flat $h^{10}$BN/calcite region (from Fig. 3b), recorded at 1460 cm$^{-1}$. **b**, Filtered real-space image obtained by applying

spatial‐frequency filtering to the experimental data in Fig. 3e, in which the wavevector components corresponding to the substrate‐coupled $M_{1,h}$ and $M_{1,t}$ modes have been removed. **c**, Line profiles (symbols) extracted from the flat region in (a) and across the wrinkle in the filtered image (b). Solid lines are fits using a damped‐cosine model.

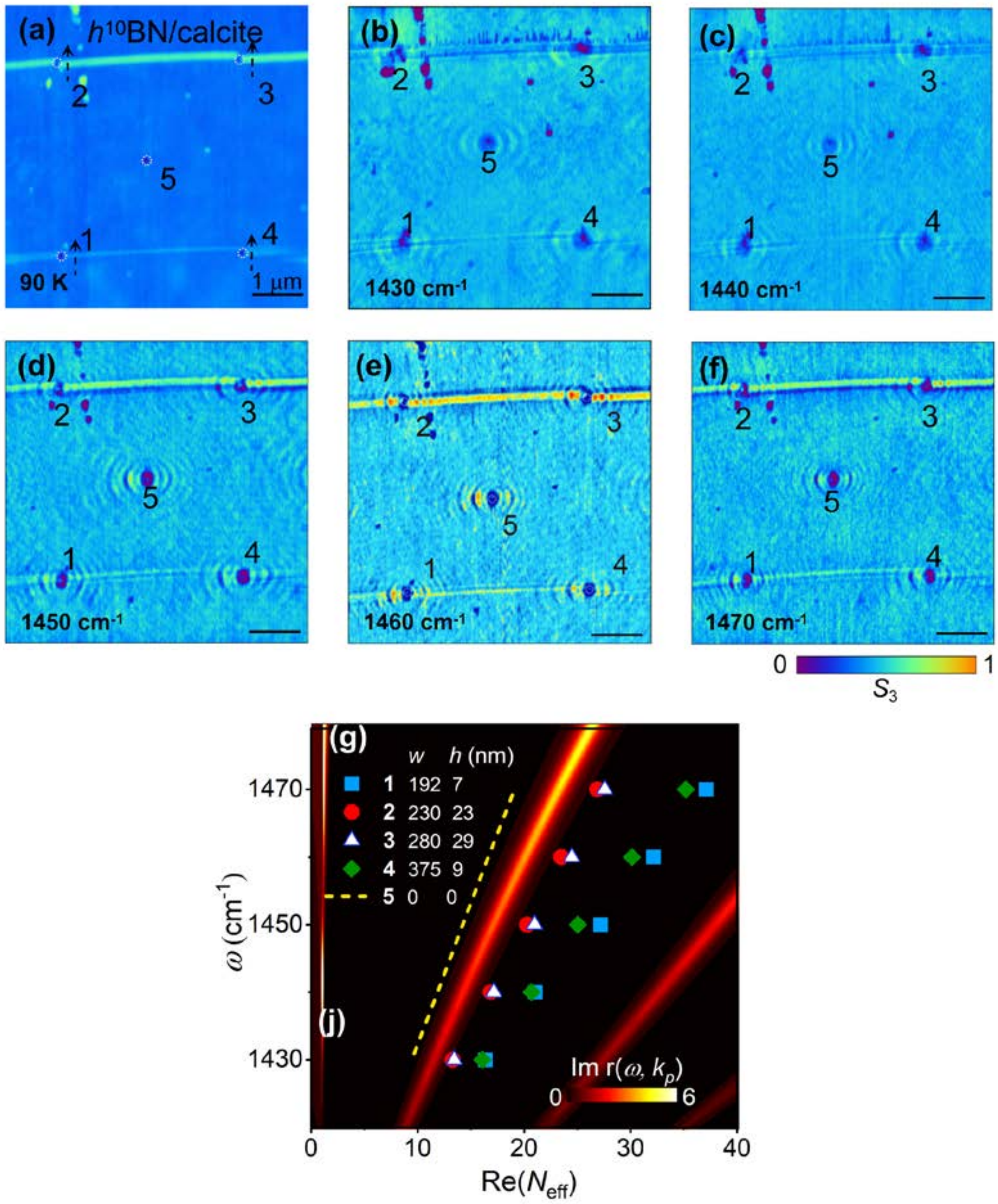


**Supplementary Fig. 11 | Frequency-dependent $M_g$ mode propagation in wrinkled *h*[10]BN/calcite heterostructures. a**, AFM topography of a 60-nm-thick *h*[10]BN/calcite heterostructure with a multi-wrinkle system induced by hole arrays (No. 1 to 5). **b-f**, Near-field image of polaritons on the multi-wrinkle system at different frequencies, with four different wrinkle geometric structures (No. 1-4). **g**, Dispersion extracted via near-field imaging (in **b-f**) along the *x* direction of the wrinkled *h*[10]BN/calcite heterostructure. False color image shows

the imaginary part of the calculated Fresnel reflection coefficient of $h^{10}$BN/calcite heterostructure.

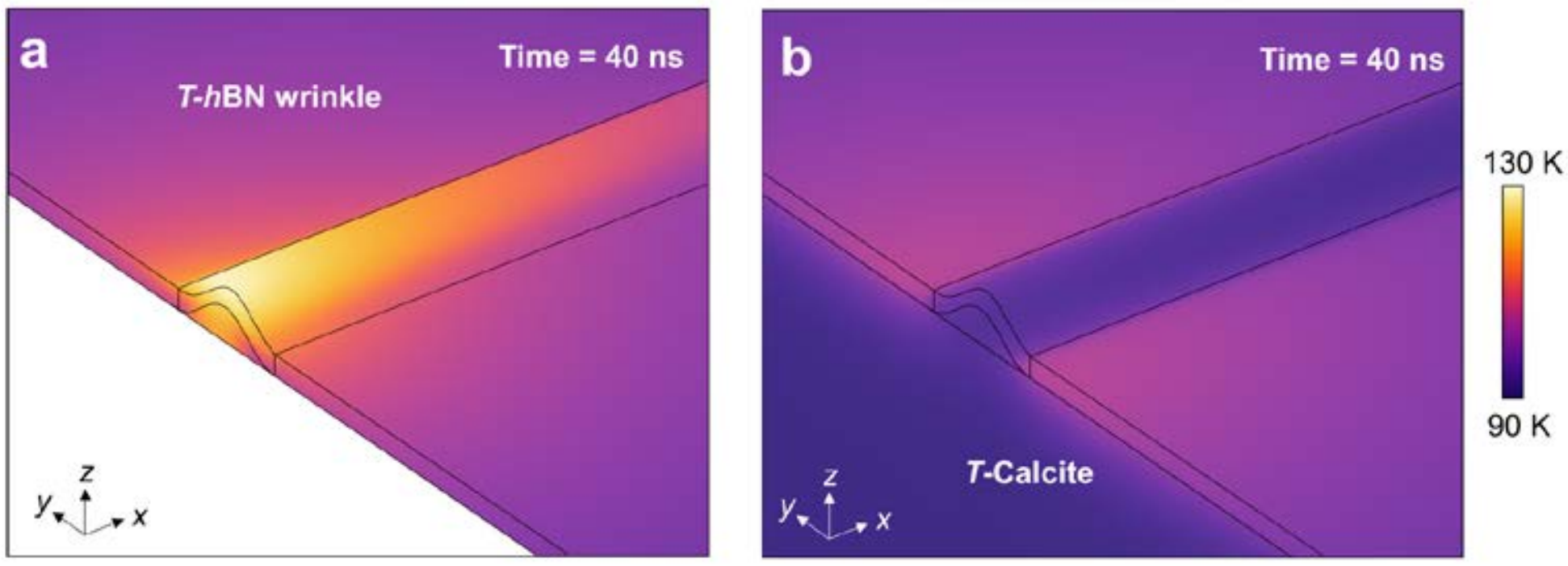


**Supplementary Fig. 12 | Steady-state temperature distributions of wrinkled $h^{10}$BN layer and calcite substrate. a**, Wrinkled $h^{10}$BN layer. **b**, Calcite substrate. Time = 40 ns. (**a**) and (**b**) share the same false color ruler.

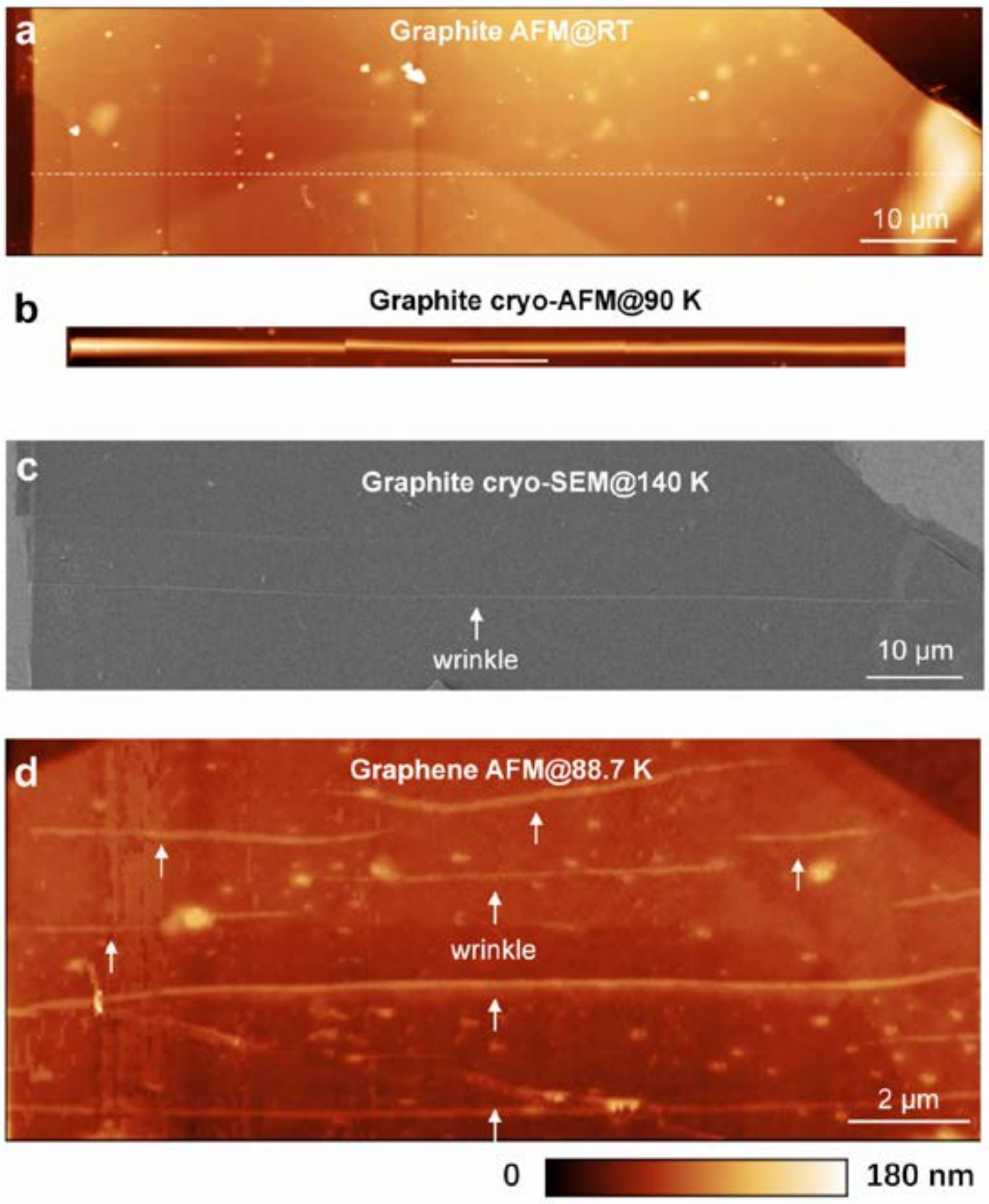


**Supplementary Fig. 13 | Temperature-induced wrinkling evolution in a graphite flake observed by AFM and cryo-SEM. a**, AFM topography image of a graphite flake at room temperature. The white dashed line indicates the positions where wrinkles form at low

temperatures. **b,** AFM topography image showing the wrinkles formed on the same graphite flake as in (**a**) at 90 K. Scale bar: 10 μm. **c**, Cryo-SEM image of the same graphite flake, revealing a horizontal wrinkle that spans the entire graphite. **d**, AFM topography image of a 10-nm graphene flake, exhibiting discontinuous bending wrinkles that readily form during cooling, attributed to the flake's low bending stiffness. White arrows in (**c**) and (**d**) indicate the positions of wrinkles.

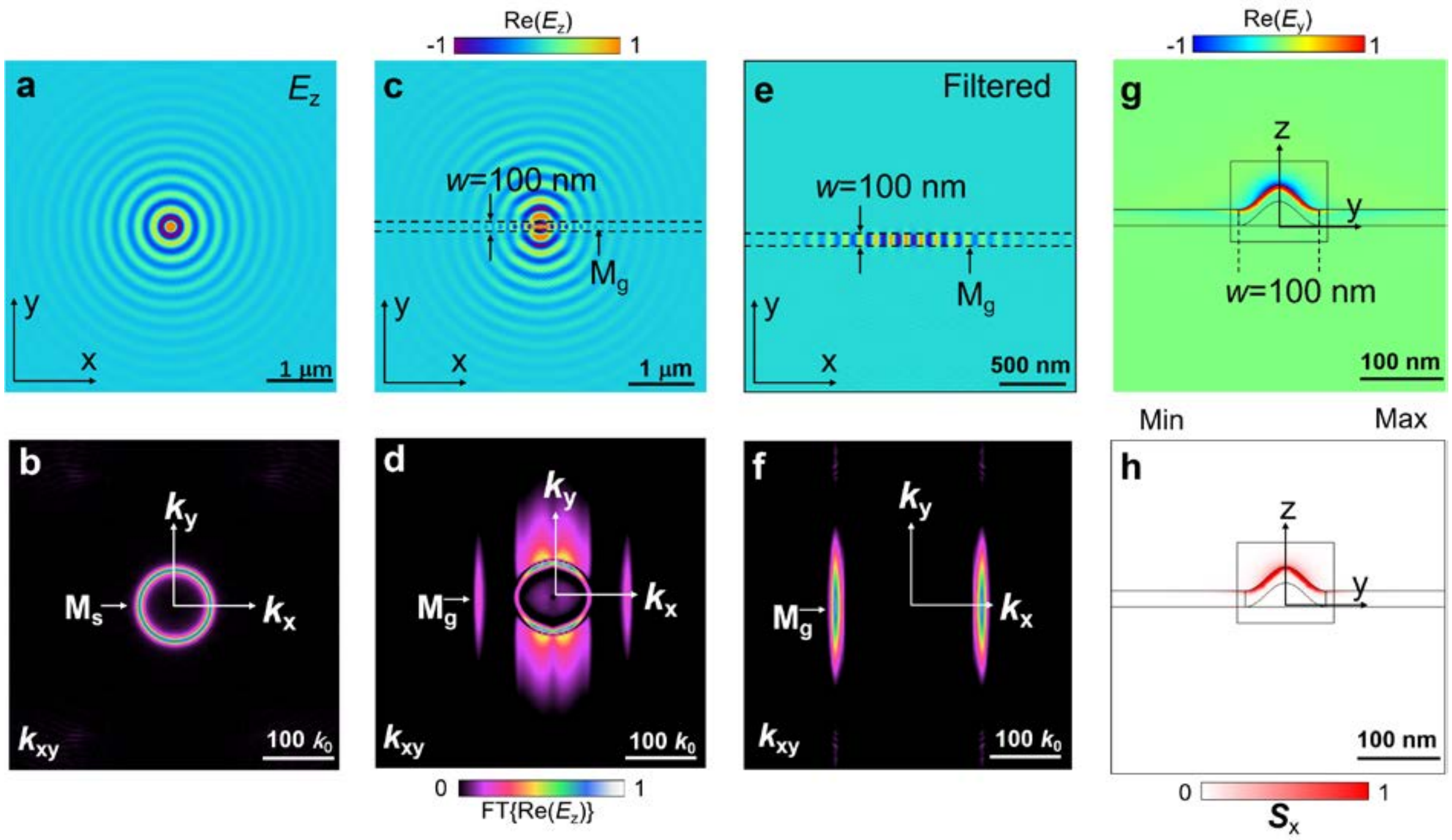


**Supplementary Fig. 14 | Channeling of a plasmon polariton mode ($M_g$) by a wrinkled graphene nanosheet on *h*BN/calcite composite substrates. a**, A simulated snapshot of the plasmon polariton wavefronts launched by a *z*-oriented dipole located 100-nm above the surface of a monolayer graphene, the excitation frequency is 860 $cm^{-1}$. **b**, Fourier transform of (**a**), indicating the plasmon polariton mode ($M_s$, $k_{Ms,x}$=48.75$k_0$) has been excited by the dipole. **c**, Simulated wavefronts at the surface of a wrinkled graphene, which is formed by the underlying *h*BN wrinkle. The width of the wrinkle *w* is 100 nm, and the height *h* is 30 nm. The dipole is located 100 nm above the crest of the wrinkle. A new plasmon polariton mode ($M_g$) can be identified. This mode propagates in the ±*x* direction and has a significantly shorter wavelength compared to $M_s$. **d**, Fourier transform of (**c**). It clearly shows that the $M_g$ mode ($k_{Mg,x}$=104.58$k_0$) in the *x*-direction has a much larger wavevector than the $M_s$ mode. **e**, Filtered real-space image of the simulated electric field after applying a spatial frequency filter to the Fourier transform in (**c**). **f**, Corresponding filtered momentum-space map of (**e**). **g**, Cross-sectional mode profile

of $M_g$ in (**e**), with the in-plane component of the optical field $E_y$ shown. **h**, Corresponding cross-sectional real part of $S_x$ distributions of the $M_g$ mode.

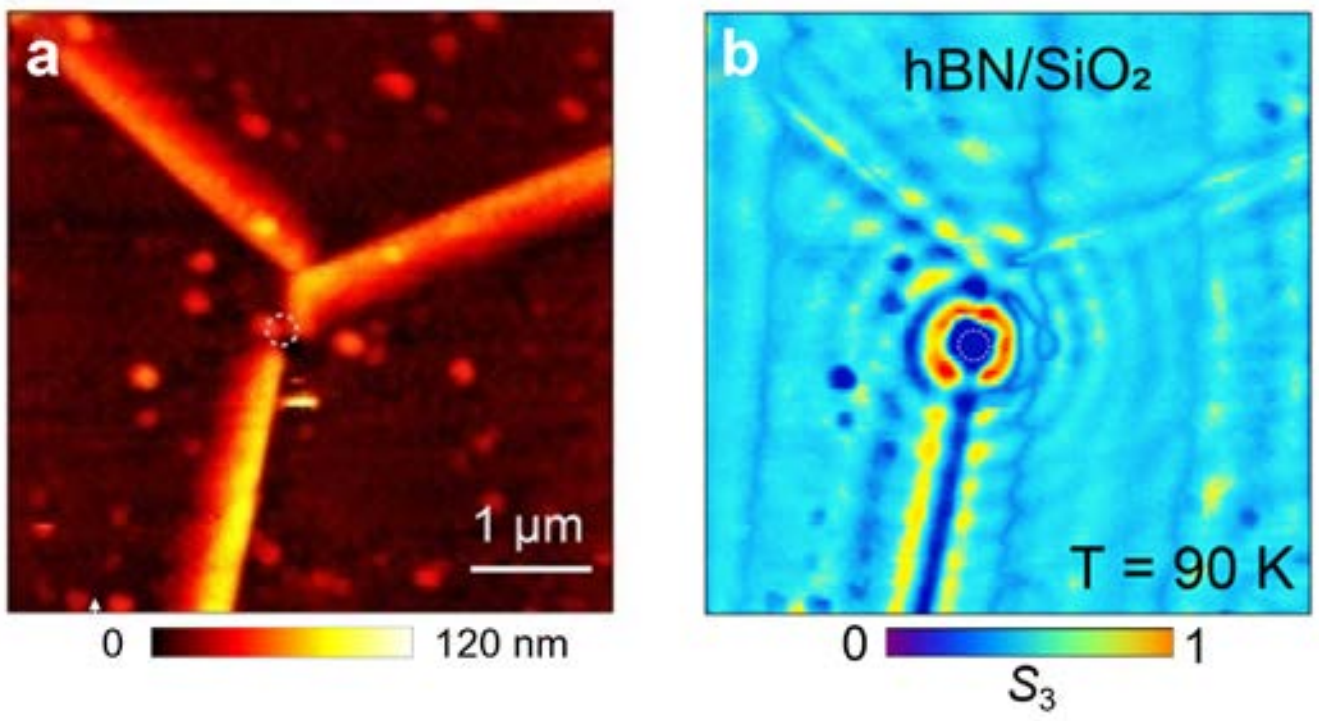


**Supplementary Fig. 15 | Triple-symmetry wrinkle network formed on an isotropic $SiO_2$ substrate. a**, Near-field optical image ($\omega$=1550 cm$^{-1}$) and **b**, corresponding AFM topography of an *h*BN flake on $SiO_2$ substrate at *T*=90 K. The images reveal a two-dimensional wrinkle network with threefold symmetry emanating from a central hole.

## Supplementary References

[1] Hellwege, K.H., Lesch, W., Plihal, M. & Schaack G. Zwei-Phononen-Absorptionsspektren und Dispersion der Schwingungszweige in Kristallen der Kalkspatstruktur. *Z Physik* **232**, 61‑86 (1970).

[2] Giles, A. et al. Ultralow-loss polaritons in isotopically pure boron nitride. *Nature Mater* **17**, 134‑139 (2018).

[3] Chen, X., Chen, Y., Yan, M. & Qiu, M. Nanosecond Photothermal Effects in Plasmonic Nanostructures. *ACS Nano* **6**, 2550‑2557 (2012).

[4] Yang, B., Pan, D. & Dai, Q. Phonon Polaritons: A New Paradigm for Light-Controllable Heat Sources. *Adv Optical Mater* **11**, 2301080, (2023).

[5] Hu, H. et al. Active control of micrometer plasmon propagation in suspended graphene. *Nat Commun* **13**, 1465 (2022).
