# Supplementary material for: Nano-wrinkle waveguides for programmable polariton channeling
Source: Nat Commun. 2026 Sep 17;17:10905. doi: 10.1038/s41467-026-77593-y (PMC13677736; doi:10.1038/s41467-026-77593-y)
Supplement: Supplementary file 2 [file 41467_2026_77593_MOESM3_ESM.pdf]

nature portfolio

Peer Review File

Nano-wrinkle waveguides for programmable polariton channeling

Corresponding Author: Professor Qing Dai

This file contains all reviewer reports in order by version, followed by all author rebuttals in order by version.

Version 0:

Reviewer comments:

Reviewer #1

(Remarks to the Author)
The manuscript addresses a topic of potential interest, that is, the programmable guiding or channeling of phonon polaritons with van der Waals crystals. While this research topic is relevant and potentially meaningful, the study does not demonstrate a clear advancement beyond existing work in the field. The proposed approach and findings appear incremental rather than innovative. Moreover, the amount and quality of experimental data presented are inadequate to substantiate the authors' conclusions. Additional experiments, broader datasets, or more rigorous analyses are needed to convincingly support the claims and strengthen the scientific validity of the work. The main concerns are as follows:

1. Lack of novelty: The work does not provide a sufficiently attractive idea. The direction of thermal expansion mismatch looks strongly dependent on the crystal structure of the calcite substrate, that means there are only one fixed "channeling" direction in this structure. Moreover, the position of the wrinkle looks appear randomly and can not be controlled on demand. Therefore, the method proposed here shows poor controllability.

2. Insufficient data and analysis: The experimental data are limited and do not adequately support the stated hypotheses or conclusions. More comprehensive experiments and analyses are needed. a) Whether the channeling of polaritons actually happens was not confirmed by the current results. According to the data (both from the simulation and experiment) the polaritons are not well confined by the channel. The observation in Fig 3e is likely due to the scattering of PhPs by the sidewall of the wrinkle while the waves spreading to the outside of the wrinkle. b) Another possible origin is the reduction of propagating loss induced by the suppression of near-field interaction between hBN PhPs and the calcite substrate suspending structure. c) Most importantly, the propagating loss the PhPs is substantially high in the channel, which means the claims on guiding the heat energy is not very convincing.

3. Weak scientific justification: The results presented fail to convincingly answer the research questions posed in the introduction. The logical connection between the data and the conclusions is not well established. a) For example, the heat energy is generally distributed across the surface with random polarization. While the result demonstrates the guiding of a point-dipole excited phonon polaritons with a h-BN wrinkle. What is the connection between these two phenomenon.

Reviewer #2

(Remarks to the Author)
In this study, the authors propose an innovative approach for creating reconfigurable waveguide channels by exploiting the hyperbolic thermal expansion mismatch between hexagonal boron nitride (hBN) thin films and calcite substrates. This method successfully demonstrates decoupled polariton propagation, exhibiting promising potential for directional energy transport applications. The claims are substantiated through comprehensive experimental and theoretical analyses, and the manuscript is well-structured. I do, however, have some minor comments that the authors should address to improve the manuscript prior to acceptance as follows:
(1) It is easy to understand that mismatch in the thermal coefficient can lead to wrinkles, which is usually random. What amazed me is that a single well-confined wrinkle of tens of microns such as the one shown in Figure 1c can be achieved. What are the conditions and general principles for such wrinkles? What is the upper limit of wrinkles in size? Any special

requirements for the substrate and the top tin vdW layers? Is it possible to expand then method and design more complex wrinkles?

(2) Some of the wrinkles beside the hole were not fully aligned. How would this affect the dispersion and propagations

(3) In the thermal analysis, the author team reported a temperature rise of a few tens of K, this is a rather large temperature span, how would this affect the properties of these reported modes?

(4)In Page 10, author claimed that" Crucially, in this regime, the wrinkle segments suspended above the substrate (with heights exceeding the critical decoupling threshold 50 nm) exhibit near-adiabatic behavior due to suppressed interfacial thermal losses to the substrate." However, even through the wrinkle are not in contact with substrate, they were still connected to the substrate with the neighboring hBN layer, which usually have high thermal conductivities. Would the near adiabatic still holds? How would this affect the thermal analysis?

Reviewer #3

(Remarks to the Author)
The manuscript presents a thermo-mechanical method to induce straight, high-curvature nano-wrinkles (channel-like ridges) in isotopically enriched $h^{10}BN$ on calcite via anisotropic thermal-expansion mismatch. Those hBN nano-wrinkles act as channel waveguides that confine a guided phonon-polariton mode with propagation predominantly along the wrinkle axis. The authors use cryogenic near-field optical microscopy (cryo-SNOM) and simulations to show that increasing wrinkle height weakens substrate influence and pushes the guided mode toward a freestanding-like regime, while nanoholes can program wrinkle placement. Electromagnetic and thermo-optic simulations further indicate these channels could facilitate polariton-mediated directional heat transport at cryogenic temperatures. The authors also show the approach is generalizable to other vdW polaritons (such as plasmon polaritons), positioning nano-wrinkle waveguides as a platform for on-chip polariton circuits and cryogenic thermal management. In my opinion, this manuscript is of interest to the field and can be published in Nature Communications after addressing the following points:

1. The authors should mention why they chose $h^{10}BN$ at the beginning.
2. What is the advantage of this proposed method over others for creating those nano-wrinkles, such as pre-strained elastomer, patterning?
3. What is the blue solid line in Figure 1a?
4. How do authors justify the permittivity value of $h^{10}BN$? Strain and temperature may shift phonon resonances in the Reststrahlen bands.
5. Since a new guided mode is claimed, it will be useful to plot representative s-SNOM line profiles in Fig. 3 and compare them with profiles from an unwrinkled region.
6. What happens when the sample returns to room temperature? Upon re-cooling below the critical temperature, do the same wrinkles re-form? How about the yield and repeatability?
7. Authors should also mention more details on how they fabricate those nano-holes on the samples. Since the title of the manuscript is programmable, the authors should also highlight this.

Version 1:

Reviewer comments:

Reviewer #1

(Remarks to the Author)
I would like to thank the authors for their efforts in revising the manuscript and addressing my previous concerns. However, after carefully reviewing the revised version and the response letter, I regret to say that my fundamental concerns regarding the novelty and the robustness of the results remain unresolved.

1) The main concern is whether one can call the propagating waves whitin the wrinkle a "channelling" mode. Indeed, a guided waves with distinct wavevector is observed in the nano wrinkle, according to the FFT image. However, there is a large part of polaritons enenrgy leaks out to the substrated-supported region, which means the observed wave is actually a hybrid polaritons mode with rather weak optical confinement.

2) I understand that scanning near-field optical imaging in cryogenic environment is tricky, however, the quality (i.e. the low signal-to-noise) of the experimental s-SNOM images can lead to the loss of key information, especially the wave propagates outside the wrinkle.

3) Along with point 2, the anisotropy maybe enhanced by the detection scheme of s-SNOM. For example, Fig.3b shows an anisotropic propagation of polaritons within a flat h-BN slab, which in fact should exhibit an isotropic wavefronts. This detection artifact indicated that the anisotropy of the claimed channelling polariotns (Fig.3e, the key experimental evidence) may also be strongly overestimated.

In conclusion, despite the revisions, the manuscript still fails to demonstrate a clear and significant contribution to the field.

Therefore, I cannot recommend this paper for publication in Nat.Commun. in its current form.

Reviewer #2

(Remarks to the Author)
The author team have carefully addressed all my comments and improved the manuscript accordingly. Overall I believe this is a well-polished manuscript for the journal.

Reviewer #3

(Remarks to the Author)
The authors have adequately addressed my concerns through sufficient revision and the addition of important new experimental and simulation results.

Regarding the remaining concerns raised by referee #1:

Reviewer #1 raises an important concern regarding whether the observed mode should be described as a true “channeling” mode. I agree that this is important, especially since the wrinkle is narrow and cryogenic near-field imaging is experimentally challenging, which likely limits the SNR compared with room-temperature s-SNOM measurements.

That said, I do think the manuscript already provides meaningful evidence for a wrinkle-induced polariton mode. In particular, the FFT analysis and FEM simulations support the existence of a mode with a distinct wavevector from the normal substrate-supported mode, and the simulated field profiles indicate localization around the wrinkle region. In addition, the waves observed outside the wrinkle should not simply be interpreted as leakage from the wrinkle-guided mode, since the nano-hole acts as a dipole polariton launcher and will inevitably excite the conventional mode around the hole as well. If one takes a look at their Supplementary Fig. 9. The guided mode at wrinkle strictures is actually distinguishable from the conventional mode, and its dispersion deviates accordingly.

I would however encourage the authors to provide more quantitative analysis of the mode, such as decay length, and a clearer comparison between propagation inside and outside the wrinkle. If available, additional data showing how the mode evolves with wrinkle geometry, including wrinkle width and how it compares to the suspended hBN mode, would further support the waveguide interpretation. Otherwise, the claim of a tightly confined “channeling” mode should be stated somewhat more cautiously.

In my view, this issue does not undermine the overall significance of the work. A major strength of the manuscript is the thermo-mechanical strategy itself, where one can create and reversibly control nano-wrinkle structures through thermal tuning, which is interesting and potentially generalizable to other vdW materials, and possibly to other thin-film material systems.

Version 2:

Reviewer comments:

Reviewer #1

(Remarks to the Author)
The authors have adequately addressed my concerns through their revision and the addition of important new experimental and simulation results. I strongly recommend that these new results should be added to the paper.

Reviewer #3

(Remarks to the Author)
The authors have satisfactorily addressed my concerns through the revisions. I appreciate their efforts and recommend the manuscript for publication.

Open Access This Peer Review File is licensed under a Creative Commons Attribution 4.0 International License, which permits use, sharing, adaptation, distribution and reproduction in any medium or format, as long as you give appropriate credit to the original author(s) and the source, provide a link to the Creative Commons license, and indicate if changes were made.
In cases where reviewers are anonymous, credit should be given to 'Anonymous Referee' and the source.
The images or other third party material in this Peer Review File are included in the article's Creative Commons license, unless indicated otherwise in a credit line to the material. If material is not included in the article's Creative Commons license and your intended use is not permitted by statutory regulation or exceeds the permitted use, you will need to obtain permission directly from the copyright holder.
To view a copy of this license, visit https://creativecommons.org/licenses/by/4.0/

Point-by-point responses to reviewers

We thank all reviewers for their insightful and constructive evaluation of our manuscript. Reviewer #2 described the approach as "innovative" and noted that it "successfully demonstrates decoupled polariton propagation," while Reviewer #3 considered the work "of interest to the field" and suitable for publication in Nature Communications after revisions. We also acknowledge the critical concerns raised by Reviewer #1 regarding the novelty and the strength of the evidence, which have prompted us to substantially expand our experimental and analytical support.

Please see our point-by-point responses (in blue font) to reviewers below. Their comments have greatly helped us to improve the manuscript.

Reviewer #1

Comments: The manuscript addresses a topic of potential interest, that is, the programmable guiding or channeling of phonon polaritons with van der Waals crystals. While this research topic is relevant and potentially meaningful, the study does not demonstrate a clear advancement beyond existing work in the field. The proposed approach and findings appear incremental rather than innovative. Moreover, the amount and quality of experimental data presented are inadequate to substantiate the authors' conclusions. Additional experiments, broader datasets, or more rigorous analyses are needed to convincingly support the claims and strengthen the scientific validity of the work. The main concerns are as follows:

Responses: We thank the Reviewer for the thorough evaluation of our manuscript. We appreciate the feedback regarding the perceived novelty and the sufficiency of experimental evidence. In response, we have expanded both the experimental data and theoretical analysis in the revised manuscript. In the following, we address the concerns of the reviewer point-by-point. The relevant changes have been listed at the end of each point (also summarized at the end of this response letter) and highlighted in the revised manuscript.

1. Lack of novelty: The work does not provide a sufficiently attractive idea. The direction of thermal expansion mismatch looks strongly dependent on the crystal structure of the calcite substrate, that means there are only one fixed "channeling" direction in this structure. Moreover, the position of the wrinkle looks appear randomly and cannot be controlled on demand. Therefore, the method proposed here shows poor controllability.

We thank the reviewer for raising these critical points. We respectfully disagree that the directional dependence represents a limitation, and we provide additional experimental evidence demonstrating the repeatability and controllability of wrinkle formation (including spatial positioning, geometric tunability, and cyclic stability). Our

response is structured below into two parts: (1) viewpoint on novelty, and (2) experimental evidence for controllability and corresponding manuscript revisions.

(1) Viewpoint: The directional dependence is a design feature, not a limitation

The reviewer rightly points out the directional dependence on the calcite crystal structure. We respectfully submit that this directional dependence is not a limitation but the core design principle, which has not been previously explored for polariton waveguiding. Existing strategies for vdW polariton manipulation (e.g., gating, doping, twisting) operate exclusively within the plane. However, the inherently planar and homogeneous nature of vdW materials precludes lateral confinement, thus preventing the formation of true channel waveguide modes. Our method introduces a geometric degree of freedom by transforming a planar 2D sheet into a suspended quasi-1D waveguide with nanoscale curvature. Rather than passively accepting a fixed direction, we actively engineer a uniaxial compressive stress by leveraging the hyperbolic thermal expansion of calcite, forcing deterministic buckling along a single, well-defined axis. This strategy overcomes the intrinsic planarity limitation of vdW materials and establishes a waveguide channel for polariton routing.

(2) Experimental evidence for controllability

We thank the reviewer for this concern. We can demonstrate controllability of wrinkle formation in three aspects: location, geometry, and repeatability.

- **Spatial controllability via pre-patterned nucleation sites**: Wrinkle position is not random but can be programmed. We fabricate nano-holes in the h^{10}BN nanosheets. These holes act as stress concentrators that locally intensify thermal stress and weaken interfacial adhesion, making them preferred nucleation sites. Upon cooling, wrinkles form at these predefined locations (Supplementary Fig. 2), demonstrating that spatial positioning is programmable by design.
- **Geometric controllability via temperature:** Variable-temperature measurements reveal that the average wrinkle height decreases linearly with increasing temperature (Supplementary Fig. 3). This linear dependence of wrinkle height enables programmable control over wrinkle geometry through thermal tuning.
- **Cyclic controllability and repeatability:** To systematically show the controllability and repeatability of the wrinkle formation process, we performed new multiple cooling-heating cycles on a graphite/calcite heterostructure (Fig. R1). AFM imaging across four cycles reveals that wrinkles vanish upon warming and deterministically re-form at nearly identical locations upon re-cooling, demonstrating positional repeatability. Furthermore, the wrinkle geometry (height and width) is both tunable and reproducible under controlled thermal conditions. A larger temperature drop (e.g., cooling from 473 K in Cycle-2, Fig. R1) produces a larger wrinkle, while re-applying the same thermal history (Cycle-4) yields dimensions that closely match those of a prior cycle (Cycle-2), with variations within ~5%. These findings establish that the thermal-buckling process is

reversible, spatially deterministic, and geometrically controllable, highlighting its potential for creating reconfigurable nanophotonic elements.

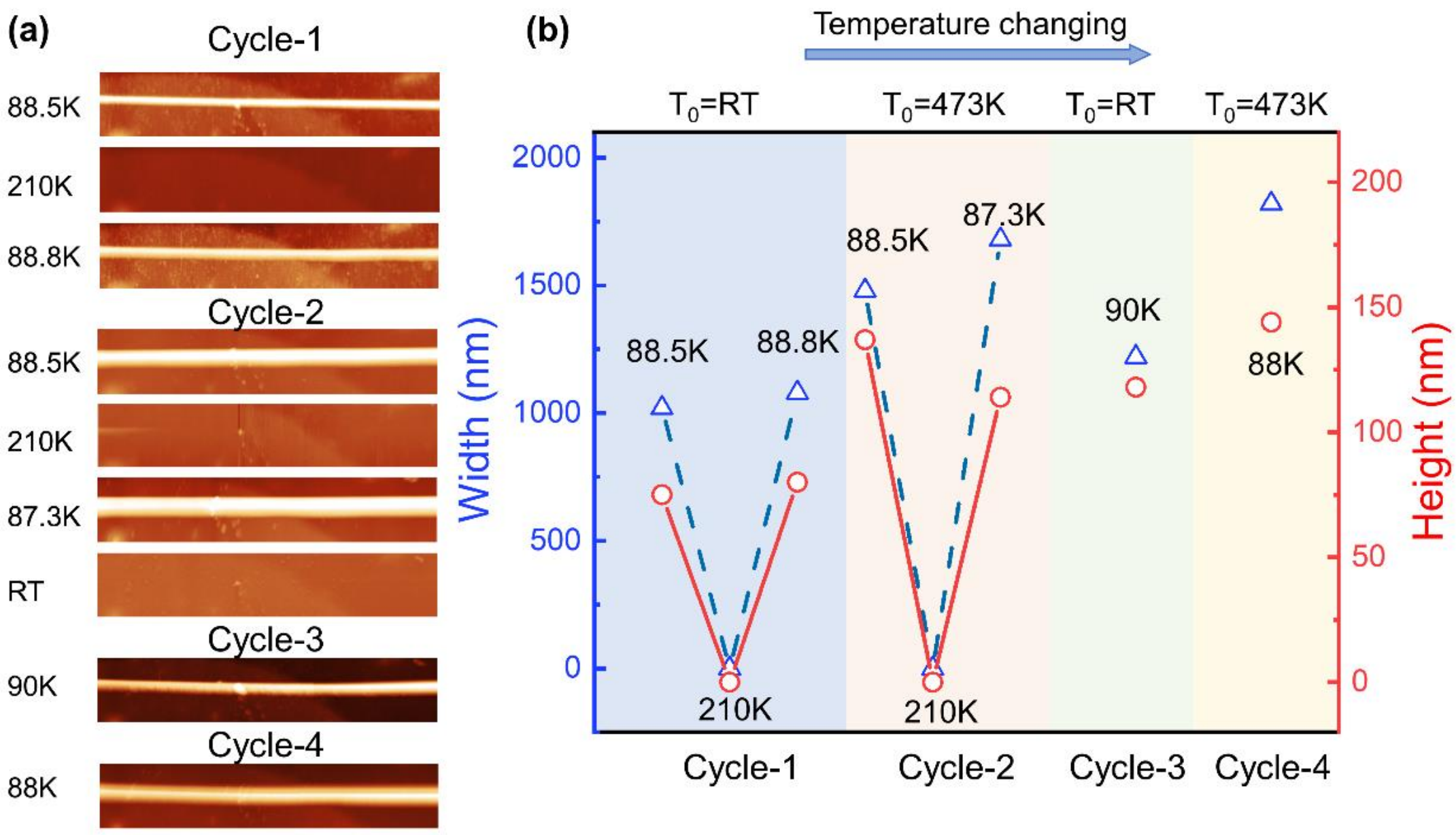

Fig. R1. | Reversibility and tunability of nano-wrinkles under cyclic thermal loading. (a) AFM topography images of a wrinkled graphite/calcite heterostructure across four thermal cycles (Cycle-1 to Cycle-4), demonstrating deterministic wrinkle re-formation at nearly identical locations after repeated cooling below and warming above the critical temperature. (b) Statistical analysis of wrinkle dimensions (height and width) extracted from the cycles in a. The height and width are tunable and repeatable under controlled thermal conditions: a larger temperature drop (Cycle-2, pre-heated to 473 K before cooling) produces a significantly larger wrinkle compared to a moderate drop (Cycle-1, RT cooling to 88.5K). Following a large-strain cycle, partial delamination can lead to increased dimensions (Cycle-3, RT cooling to 90K). When the same thermal swing is reapplied (Cycle-4), the wrinkle dimensions closely match those of Cycle-2 (within ~5% variation), confirming that reproducible morphology is achieved with a consistent thermal history.

To resolve the concerns of the reviewer, we have made the following changes in the revised manuscript:

(1) added the following explanation "To systematically show the reversibility and repeatability of the wrinkle formation process, multiple cooling-heating cycles are performed on a graphite/calcite heterostructure. Variable-temperature AFM imaging (Supplementary Fig. 4a) demonstrates the reversibility: wrinkles vanish as the film returns to a flat state upon warming above the critical temperature and re-form at nearly identical locations upon subsequent re-cooling. The wrinkles deterministically repeat their spatial positions across four thermal cycles (Cycle-1 to Cycle-4), confirming that nucleation is governed by interfacial properties. Furthermore, the wrinkle geometry (height and width) is both tunable and reproducible under controlled thermal conditions.

A larger temperature drop (e.g., cooling from 473 K in Cycle-2, Supplementary Fig. 4b) produces a larger wrinkle, while re-applying the same thermal history (Cycle-4) yields dimensions that closely match those of a prior cycle (Cycle-2), with variations within ~5%. These findings establish that the thermal-buckling process is reversible, spatially deterministic, and geometrically controllable, highlighting its potential for creating reconfigurable nanophotonic elements." to the main text (**page 6, paragraph 2**);

(2) added **Fig. R1** to the Supplementary Information as **Supplementary Fig. 4**.

2. Insufficient data and analysis: The experimental data are limited and do not adequately support the stated hypotheses or conclusions. More comprehensive experiments and analyses are needed. a) Whether the channeling of polaritons actually happens was not confirmed by the current results. According to the data (both from the simulation and experiment) the polaritons are not well confined by the channel. The observation in Fig 3e is likely due to the scattering of PhPs by the sidewall of the wrinkle while the waves spreading to the outside of the wrinkle. b) Another possible origin is the reduction of propagating loss induced by the suppression of near-field interaction between hBN PhPs and the calcite substrate suspending structure. c) Most importantly, the propagating loss the PhPs is substantially high in the channel, which means the claims on guiding the heat energy is not very convincing.

We thank the reviewer for raising these critical points regarding the evidence for polariton channeling, confinement, and the implication for heat transport. We agree that a more direct and comprehensive analysis is essential to substantiate our claims. In response, we have performed new data analysis and provide further evidence from both simulations and experiments to address these concerns directly.

a) & b) Direct evidence for the guided M_g mode and its confinement

We respectfully disagree with the reviewer's suggestion that the channeling of polaritons does not occur. Simulation results in Fig. 2 demonstrate the existence of a wrinkle-confined guided mode (M_g), which is absent in the unwrinkled region (Fig. 2a). Fig. 2c reveals M_g mode propagating along the wrinkle in the $\pm x$ direction with wavevector k_{Mgx}=24.58k_0. Mode analysis reveals tight confinement of the M_g mode within the wrinkle (Fig. 2e), with an effective refractive index (Re(N_{eff})=24.65) matching its momentum-space peak in Fig. 2d—directly proving that this mode is geometrically confined by the wrinkle rather than arising from scattering.

The raw near-field images (Fig. 2c and 3e) contain a superposition of signals from planar modes (M_1, M_2) and the guided M_g mode, which may give the visual impression of poor confinement. To isolate M_g mode, we applied spatial frequency filtering to both our simulations and experimental data (Fig. R2):

- **Simulation evidence:** By applying spatial frequency filtering to Fig. 2d, the resulting filtered real-space image (Fig. R2a) clearly shows wavefronts confined to and propagating exclusively along the wrinkle axis, while the corresponding filtered momentum-space map (Fig. R2b) exhibits a sharp IFC centered at $k_{Mg,x}$=24.58k_0.

- **Experimental confirmation:** Applying the same filter to cryo-SNOM data (Fig. 3e) reveals guided wavefronts (Fig. R2c) with IFC peak at $k_{Mg,x}$=23.82k_0 (Fig. R2d).

Furthermore, we extracted a line profile (Fig. R2e) from the filtered image (Fig. R2c) across the wrinkle and compared it with a profile from the flat region in Fig. 3b. the wrinkle exhibits a single periodic fringe (287 nm period) corresponding to M_g, while the flat region shows two distinct periods from planar modes ($M_{1,h}$, $M_{1,t}$). These results establish that the wrinkle supports a new channel mode—originating from geometric confinement, not from scattering or substrate-decoupling effects.

c) Guiding heat energy with polaritons

We respectfully disagree with the reviewer's implication that a finite polariton propagation loss undermines our claim of heat routing. We clarify that our heat-routing concept refers to **geometry-confined, polariton-mediated heat generation and subsequent directional heat flow**, not lossless power delivery to a distant terminal.

Evidence: In our thermal simulation, we treat **polaritonic dissipation as the sole heat source** (in Fig. 4 and throughout this section). Hence, the "loss" of propagating PhPs does not represent missing energy; it represents **distributed conversion of polaritonic energy into lattice heat**. The results show that both current density and power-loss density peak inside the wrinkle (Fig. 4b), confirming that the confined M_g mode localizes heat generation. This localized heating is further evidenced by the transient temperature maps (Fig. 4c), which evolve anisotropically along the wrinkle axis—consistent with guided polariton-mediated energy deposition followed by directional heat flow—rather than showing isotropic diffusion from a point source. Above the critical wrinkle height (~50 nm), suspended wrinkle segments become effectively substrate-decoupled, strongly suppressing interfacial thermal leakage and enabling near-adiabatic behavior: the wrinkled *h*BN reaches ~140 K ($\Delta T \approx 50$ K) while the calcite substrate exhibits only a much smaller temperature rise (~25 K, primarily under supported regions; Fig. 4d and Supplementary Fig. 10).

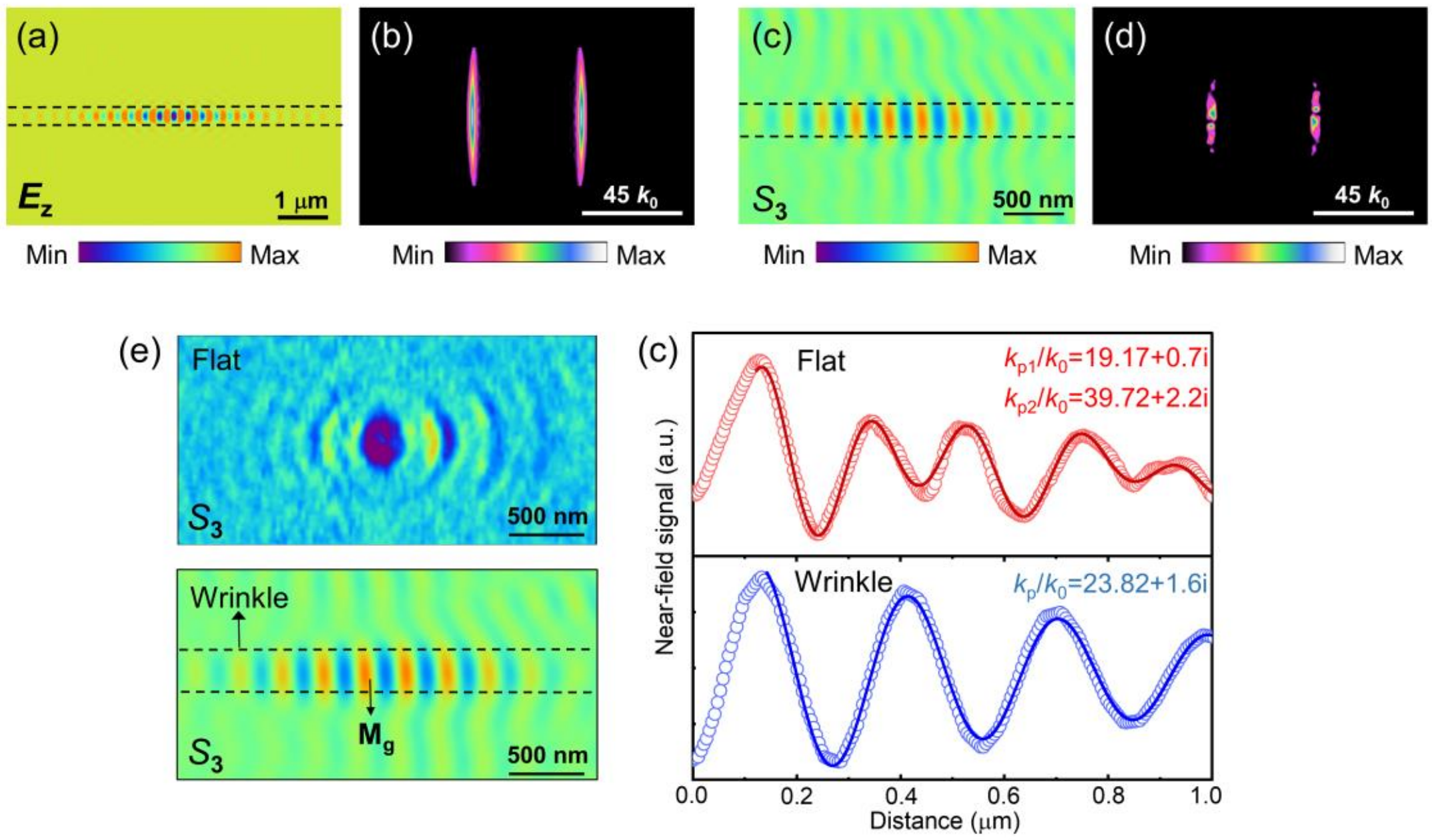

Fig. R2 | Isolation of the wrinkle-guided polariton mode via spatial frequency filtering. (a) Filtered real-space image of the simulated electric field after applying a spatial frequency filter to the Fourier transform in Fig. 2d. (b) Corresponding filtered momentum-space map of the simulation. (c) Experimentally filtered near-field image revealing guided wavefronts along the wrinkle. (d) Filtered real-space near-field amplitude obtained from experimental cryo-SNOM data (Fig. 3f) by removing the substrate-coupled $M_{1,h}$ and $M_{1,t}$ components. (e) Line profiles across the wrinkle (blue) and a flat region (red).

To resolve the concerns of the reviewer, we have made the following changes in the revised manuscript:

(1) added the following explanation "(clearly revealed in the filtered real-space image where M_1 and M_2 components are removed, Supplementary Fig. 6)" to the main text (**page 6, paragraph 3**);

(2) added **Fig. R2a, b** to the Supplementary Information as **Supplementary Fig. 6**;

(3) replaced **Supplementary Fig. 7c, d** with **Fig. R2c, d;**

(4) added **Fig. R2e** to the Supplementary Information as **Supplementary Fig. 8**;

(5) added the following explanation "By applying 2D FFT filtering to Fig. 3e, the resulting real-space near-field image (Supplementary Fig. 8b) clearly identifies the M_g mode. The line profile across the wrinkle shows a single periodic fringe corresponding to this mode, with a fitted quality factor of Q=Re(k_p)/Im(k_p)=14.9, while the profile from the flat region exhibits two distinct periods associated with the $M_{1,h}$ and $M_{1,t}$ modes (Supplementary Fig. 8c)." to the main text (**page 8, paragraph 1**).

(6) modified the following explanation "**the polariton-mediated heat generation and the subsequent directional heat flow.** This synergy—between **wrinkle**-induced

polariton confinement and suppressed thermal leakage **to the substrate**—establishes a robust, directional, and low-loss **low-thermal-leakage** mechanism for nanoscale heat routing." to the main text (**page 12, paragraph 2**).

3. Weak scientific justification: The results presented fail to convincingly answer the research questions posed in the introduction. The logical connection between the data and the conclusions is not well established. a) For example, the heat energy is generally distributed across the surface with random polarization. While the result demonstrates the guiding of a point-dipole excited phonon polaritons with a h-BN wrinkle. What is the connection between these two phenomena.

We thank the reviewer for this thoughtful comment regarding the nature of the heat source. We clarify the connection between the dipole-excited simulation and the physical heat transport mechanism as follows:

(1) **Nature of the "Heat":** Our study investigates **near-field, polariton-mediated energy dissipation**, distinct from far-field thermal radiation. The heat generation here arises from the non-radiative damping of guided polaritons within the lossy hBN, governed by electromagnetic absorption: $Q_h(\mathbf{r}) \propto \omega\, \mathrm{Im}[\varepsilon(\omega)] \mid E(\mathbf{r}) \mid^2$.

(2) **The Point Dipole as a Probe:** We employ the point dipole not to mimic a macroscopic thermal source, but to selectively excite the **channel eigenmode** (M_g) defined by the wrinkle geometry. This establishes the fundamental physical mechanism: **Geometry → Guided Mode → Confined Dissipation → Directional Heat Flow**.

(3) **Robustness against Random Polarization:** Crucially, the wrinkle waveguide acts as a **mode filter**. While a realistic heat source possesses random polarization, the wrinkle structure selectively supports and confines the $\mathbf{M_g}$ mode while suppressing others. Therefore, "random polarization" affects only the coupling efficiency, not the existence of the directional channeling pathway.

(4) **Evidence:** This mechanism is validated by the transient thermal maps (Fig. 4c), where the temperature field evolves anisotropically along the wrinkle axis, directly tracking the guided mode's dissipation profile rather than isotropic diffusion.

Reviewer #2

In this study, the authors propose an innovative approach for creating reconfigurable waveguide channels by exploiting the hyperbolic thermal expansion mismatch between hexagonal boron nitride (hBN) thin films and calcite substrates. This method successfully demonstrates decoupled polariton propagation, exhibiting promising potential for directional energy transport applications. The claims are substantiated through comprehensive experimental and theoretical analyses, and the manuscript is well-structured. I do, however, have some minor comments that the authors should address to improve the manuscript prior to acceptance as follows:

Responses: We thank the reviewer for the positive comments on our manuscript. In the following, we address the concerns of the reviewer point-by-point. The relevant changes have been listed at the end of each point (also summarized at the end of this response letter) and highlighted in the revised manuscript.

(1) It is easy to understand that mismatch in the thermal coefficient can lead to wrinkles, which is usually random. What amazed me is that a single well-confined wrinkle of tens of microns such as the one shown in Figure 1c can be achieved. What are the conditions and general principles for such wrinkles? What is the upper limit of wrinkles in size? Any special requirements for the substrate and the top tin vdW layers? Is it possible to expand then method and design more complex wrinkles?

We thank the reviewer for the insightful question regarding the deterministic formation and scalability of our nano-wrinkles. We address each point below.

1. **Conditions and general principles for a single, long-range wrinkle:**

A single, well-confined wrinkle forms due to the combination of a highly uniaxial stress field and pre-patterned nucleation sites. The in-plane thermal expansion coefficient of h^{10}BN ($\alpha_{h^{10}BN}$=-2.40 ppm/K) closely matches that of (100)-oriented calcite along its x-axis ($\alpha_{calcite,x}$=-2.42 ppm/K), while a large mismatch exists along the perpendicular y-axis ($\alpha_{calcite,y}$=15.63 ppm/K). This anisotropic mismatch creates a highly directional uniaxial compressive stress upon cooling, forcing the h^{10}BN to buckle solely perpendicular to the stress field.

2. **The upper limit of wrinkle size:**

It is challenging to provide a precise theoretical value, as the final dimensions arise from a complex interplay of film thickness, interfacial adhesion, and cooling kinetics. Experimentally, we find that the wrinkle length is primarily constrained by the lateral size of the h^{10}BN flake, often extending across its entire length and reaching hundreds of micrometers. The wrinkle height is both temperature- and cooling-rate dependent: the average height decreases linearly with increasing temperature (Supplementary Fig. 3), while the maximum achievable height is strongly influenced by the cooling rate. The largest height observed in our experiments, ~291 nm, was obtained by rapidly cooling the sample from 473 K to 90 K (Fig. R3).

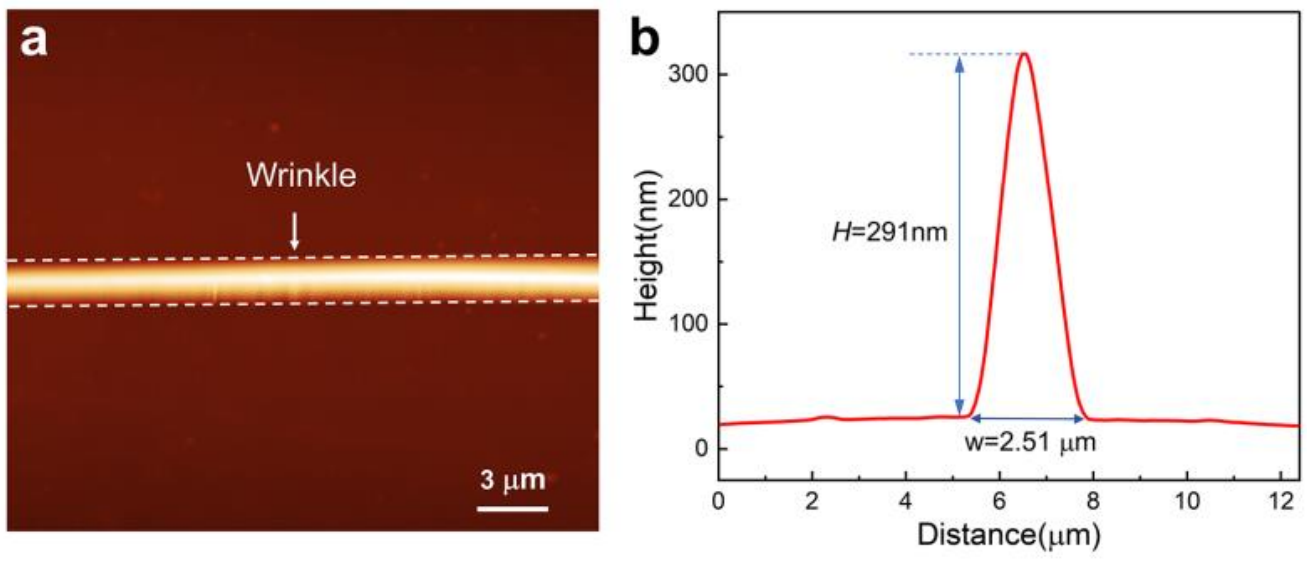

Fig. R3 | Morphology of the highest experimentally observed wrinkle. (a) AFM topography image of the maximum-height wrinkle formed under rapid cooling conditions. (b) Corresponding cross-sectional line profile extracted from (a), showing a wrinkle height of ~291 nm and a width of ~2.51 μm.

3. Special requirements:

The key requirement is a controlled uniaxial thermal-expansion mismatch between substrate and vdW layer. Simultaneously, the vdW material must possess sufficient in-plane stiffness and fracture toughness to accumulate elastic strain energy, along with moderate flexural rigidity to enable stable out-of-plane buckling with nanoscale curvature. Materials that are too soft or too thin—such as graphene or other monolayer vdW flakes—lack the necessary mechanical robustness to form straight, uniform wrinkles. As a result, such films tend to form irregular folds rather than evolving into the straight, channel-like ridges required for well-defined polariton waveguides (Supplementary Fig. 11d).

4. Expandability to more complex wrinkles:

Beyond straight *h*BN wrinkles, the method can be extended to generate more complex geometries. For instance, **using an *h*BN/calcite stack as a composite substrate** enables a monolayer transferred onto it to inherit a deterministic waveguide geometry, transforming an otherwise random wrinkle-prone film into a structured waveguide. Finite-element simulations confirm that these engineered structures effectively guide graphene plasmon polaritons (Supplementary Fig. 12). Besides, this thermal expansion mismatch approach can likewise be applied to **substrates with different symmetries**. By altering the symmetry of the stress field, the wrinkle morphology can be actively controlled. For example, on an isotropic substrate like SiO_2, isotropic compression leads to the formation of a two-dimensional triple-symmetry wrinkle network, as observed in our experiments (Fig. R4). This transition from 1D channels to 2D networks may enable interconnected polariton circuits or tunable photonic metasurfaces.

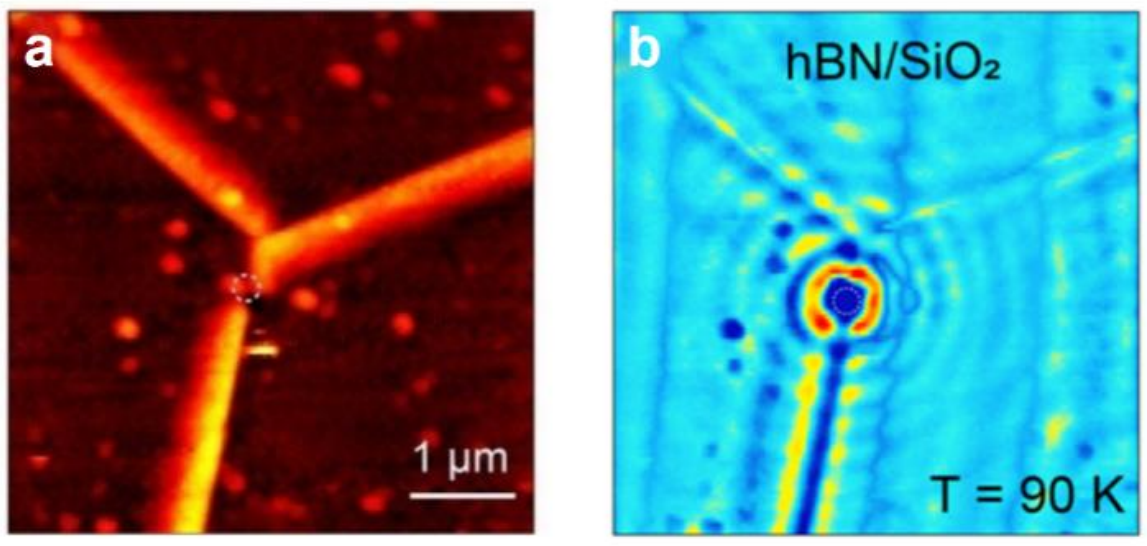

Fig. R4 | Triple-symmetry wrinkle network formed on an isotropic SiO_2 substrate. (a) Near-field optical image (ω=1550 cm^{-1}) and (b) corresponding AFM topography of an *h*BN flake on SiO_2 substrate at T=90 K. The images reveal a two-dimensional wrinkle network with threefold symmetry emanating from a central hole.

To resolve the concerns of the reviewer, we have made the following changes in the revised manuscript:

(1) modified the explanation "The net result is a **highly directional uniaxial compressive stress** acting on h^{10}BN indicated schematically in Fig. 1a, which induces a compression strain Σ within the nanosheet along the y axis**, forcing the h^{10}BN to buckle solely perpendicular to the stress field**." in the main text (**Page 3, paragraph 2**);

(2) added the following explanation "Besides, this thermal expansion mismatch approach can likewise be applied to substrates with different symmetries. By altering the symmetry of the stress field, the wrinkle morphology can be actively controlled. For example, on an isotropic substrate like SiO_2, isotropic compression leads to the formation of a two-dimensional triple-symmetry wrinkle network, as observed in our experiments (Supplementary Fig. 13). This transition from 1D channels to 2D networks may enable interconnected polariton circuits or tunable photonic metasurfaces." to the main text (**page 13, paragraph 2**);

(3) added **Fig. R4** to the Supplementary Information as **Supplementary Fig. 13**.

(2) Some of the wrinkles beside the hole were not fully aligned. How would this affect the dispersion and propagations

We thank the reviewer for this relevant observation. The slight misalignment arises from local variations in interfacial adhesion after transfer, which cause inhomogeneous stress distribution around the holes during cooling and shift the nucleation point. Further refinement of the transfer process to ensure more uniform interfacial contact will minimize these offsets. The influence of such misalignment on mode dispersion and propagation can be explained as follows:

- **Effect on dispersion**: The dispersion of the guided M_g mode is governed by the wrinkle's cross-sectional geometry (height, width, and curvature, Fig. 2f). Whenthis geometry is preserved, the effective index and dispersion relation remain unchanged, even with slight lateral offsets.

- **Effect on propagation**: Polaritons are guided along the wrinkle axis itself. In the observed cases, the wrinkles remain straight and aligned with the crystal-defined direction. Therefore, minor positional offsets do not alter the propagation path but may introduce minorpropagation loss.

(3) In the thermal analysis, the author team reported a temperature rise of a few tens of K, this is a rather large temperature span, how would this affect the properties of these reported modes?

We thank the reviewer for this important question. A temperature rise of several tens of Kelvin can indeed shift optical phonon frequencies and thus affect polariton dispersion. Based on our experimental data and analysis, we confirm that this effect is quantifiable anddoes not alter the core conclusions of our work.

- **Quantifying the temperature effect:** Our variable-temperature Raman data (Fig. 1b) provide the temperature coefficient for the E_{2g} phonon: k_{post}=−0.01892 cm^{-1}/K. Using this measured coefficient, we corrected the dielectric function of h^{10}BN and recalculated the guided M_g mode wavevector at the excitation frequency of 1460 cm^{-1}. The resulting values are 24.58k_0 at 90 K (Fig. 2) and 24.79k_0 at 140 K—a relative change of only ~0.9%.
- **Negligible laser-induced heating in near-field experiments**: In s-SNOM experiments, the focused infrared laser itself induces local heating on the order of tens of K within the probed region. This effect is routinely disregarded in standard data analysis, as the resulting variation in extracted wavevectors falls within experimental uncertainty. Therefore, the temperature increase included in our thermal simulations represents a small perturbation that does not affect our conclusions regarding geometry-dominated polariton guiding.

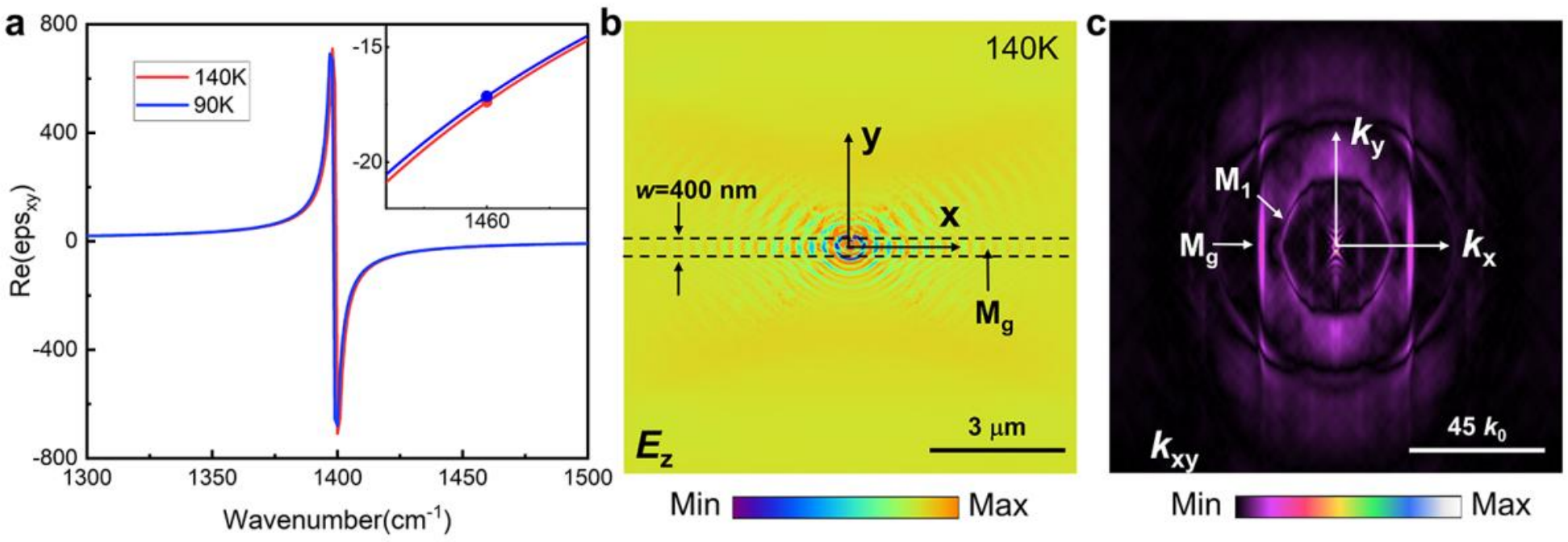

Fig. R5 | Temperature-dependent dielectric response and polariton simulation at 140 K. (a) Real part of the in-plane dielectric function of h^{10}BN at 90 K (blue) and 140 K (red), adjusted using the measured E_{2g} phonon temperature coefficient (k_{post} = −0.01892 cm^{-1}/K) from Fig. 1b. (b, c) Simulated near-field distribution (b) and its Fourier transform (c) of the Mg mode in a wrinkled h^{10}BN/calcite heterostructure at 140 K and 1460 cm^{-1}.

(4) In Page 10, author claimed that" Crucially, in this regime, the wrinkle segments suspended above the substrate (with heights exceeding the critical decoupling threshold

50 nm) exhibit near-adiabatic behavior due to suppressed interfacial thermal losses to the substrate." However, even though the wrinkle are not in contact with substrate, they were still connected to the substrate with the neighboring hBN layer, which usually have high thermal conductivities. Would the near adiabatic still holds? How would this affect the thermal analysis?

We thank the reviewer for raising this important point. We agree that a residual pathway remains via lateral in-plane conduction from the suspended segment to neighboring supported hBN and then into the substrate. We clarify our terminology and thermal model below.

- **Clarification of "near-adiabatic":** Our statement of "near-adiabatic" refers to the suppression of the dominant vertical heat-sinking pathway—direct interfacial conduction to the substrate—once the wrinkle height exceeds the critical decoupling threshold (~50 nm). It does not imply perfect thermal isolation, as lateral conduction pathways remain.
- **Model implementation and validation**: In our heat-transfer model, substrate coupling is implemented through the interfacial term $G(T_{\mathrm{hBN}}\text{-}T_s)$ (with G=70 MW m^{-2}K^{-1}), applied to the supported contact area, whereas the suspended region has no such interfacial pathway. The **lateral** pathway is **included** through the $\nabla\cdot(\mathbf{k}\nabla T)$term and is strongly limited by a series of thermal resistances (lateral spreading resistance, finite interfacial conductance $1/G$, and substrate diffusion). Using the anisotropic thermal conductivities in our simulations (suspended: $k_{\parallel}/k_{\perp}=800/4\ \mathrm{Wm^{-1}K^{-1}}$; supported: $400/2\ \mathrm{Wm^{-1}K^{-1}}$), the effective heat sinking from the suspended segment to the substrate is greatly reduced compared to the fully supported case. This is consistent with our simulated temperature fields (Supplementary Fig. 10), which show negligible substrate heating beneath suspended wrinkles while heating is localized primarily under supported regions.

To resolve the concerns of the reviewer, we have made the following changes in the revised manuscript:

(1) revise the wording to "Crucially, when the wrinkle height exceeds the critical decoupling threshold (~50 nm), the dominant vertical heat-sinking pathway—direct interfacial conduction to the substrate—is effectively removed. The remaining heat dissipation occurs primarily via lateral conduction through the h^{10}BN film toward supported regions and then into the substrate, a pathway that is inherently limited by the series of thermal resistances (lateral spreading, finite interfacial conductance, and substrate diffusion). This results in strongly suppressed substrate heat sinking for the suspended segments, as" in the main text (**page 11, paragraph 1**).

Reviewer #3

The manuscript presents a thermo-mechanical method to induce straight, high-curvature nano-wrinkles (channel-like ridges) in isotopically enriched $h^{10}BN$ on calcite via anisotropic thermal-expansion mismatch. Those hBN nano-wrinkles act as channel waveguides that confine a guided phonon-polariton mode with propagation predominantly along the wrinkle axis. The authors use cryogenic near-field optical microscopy (cryo-SNOM) and simulations to show that increasing wrinkle height weakens substrate influence and pushes the guided mode toward a freestanding-like regime, while nanoholes can program wrinkle placement. Electromagnetic and thermo-optic simulations further indicate these channels could facilitate polariton-mediated directional heat transport at cryogenic temperatures. The authors also show the approach is generalizable to other vdW polaritons (such as plasmon polaritons), positioning nano-wrinkle waveguides as a platform for on-chip polariton circuits and cryogenic thermal management. In my opinion, this manuscript is of interest to the field and can be published in Nature Communications after addressing the following points:

Responses: We thank the reviewer for the positive comments on our manuscript. In the following, we address the concerns of the reviewer point-by-point. The relevant changes have been listed at the end of each point (also summarized at the end of this response letter) and highlighted in the revised manuscript.

1. The authors should mention why they chose $h^{10}BN$ at the beginning.

We thank the reviewer for this suggestion. The reason for selecting isotopically enriched h^{10}BN is already stated at the beginning of the "**Deterministic thermal buckling of *h*BN to form nano-wrinkles**" section. We chose h^{10}BN because its in-plane thermal expansion coefficient ($\alpha_{h^{10}BN}$=−2.40 ppm/K) nearly perfectly matches that of (100)-cut calcite along the x-axis ($\alpha_{Calcite,x}$=−2.42 ppm/K), while creating a large mismatch along the y-axis ($\alpha_{Calcite,y}$=15.63 ppm/K). This precise thermal match along one crystal axis and mismatch along the other is the essential condition for generating the uniaxial compressive stress that drives deterministic, straight wrinkle formation upon cooling.

To resolve the concerns of the reviewer, we have made the following changes in the revised manuscript:

(1) highlighted the explanation "$\alpha_{\perp}$ and $\alpha_{//}$ of calcite are found to be -2.42 ppm/K and 15.63 ppm/K respectively within the temperature range from 100 K to 400 K. Noting that the average in-plane TEC of *h*BN in the same temperature interval is nearly identical to $\alpha_{\perp}$ of calcite (for natural *h*BN α_{hBN}=-2.48 ppm/K and for isotopically purified h^{10}BN $\alpha_{h^{10}BN}$=-2.40 ppm/K)" in the main text (**Page 3, paragraph 2**).

2. What is the advantage of this proposed method over others for creating those nano-wrinkles, such as pre-strained elastomer, patterning?

We thank the reviewer for this important question. Methods such as pre-strained elastomers or direct patterning have been widely used to create wrinkles in various contexts, particularly in flexible electronics and surface engineering (*Nature, 1998, 393, 146; Science, 2008, 320, 912*; *Advanced Materials, 2012, 24, 3010*). These approaches involve a stiff thin film on a compliant substrate (e.g., PDMS), where wrinkles form upon strain release. While effective for large-area periodic patterns, they present several limitations for cryogenic nanophotonics. Our thermal-buckling method offers distinct advantages that address these limitations:

(1) **Cryogenic compatibility and stability**: Conventional polymer substrates (e.g., PDMS) undergo glass transition near 153 K, rendering them mechanically unstable at cryogenic temperatures where polaritonic effects are typically investigated. Our hard single-crystal calcite substrate remains stable across the entire temperature range.

(2) **Suspended waveguide geometry:** In pre-strained elastomer systems, wrinkles remain attached to the substrate (*Science, 2008, 320, 912*), preventing the formation of suspended segments essential for strong optical confinement. Our simulations show that a suspended wrinkle achieves a wavevector of ~24.6k_0 (Fig. 2e), while an attached wrinkle of identical geometry supports a mode with only ~18.9k_0 (Fig. R6)—a ~30% increase demonstrating the advantage of suspension.

(3) **No time-dependent creep**: Polymer-based wrinkles can exhibit viscoelastic creep over time, potentially causing gradual morphology drift under sustained stress. In our system, once the wrinkle is formed and the thermal stress is released, the wrinkle geometry remains stable under constant temperature conditions, offering long-term dimensional stability.

(4) **Anisotropic optical response:** Organic substrates exhibit strong molecular vibrational bands in the mid-infrared (1000–2000 cm^{-1}), which may introduce absorption losses. In contrast, calcite's strong anisotropy actively modulates isotropic *h*BN polaritons (Fig. 2a), suppressing propagation perpendicular to the wrinkle axis and ensuring efficient channeling along the waveguide direction (Fig. 2c).

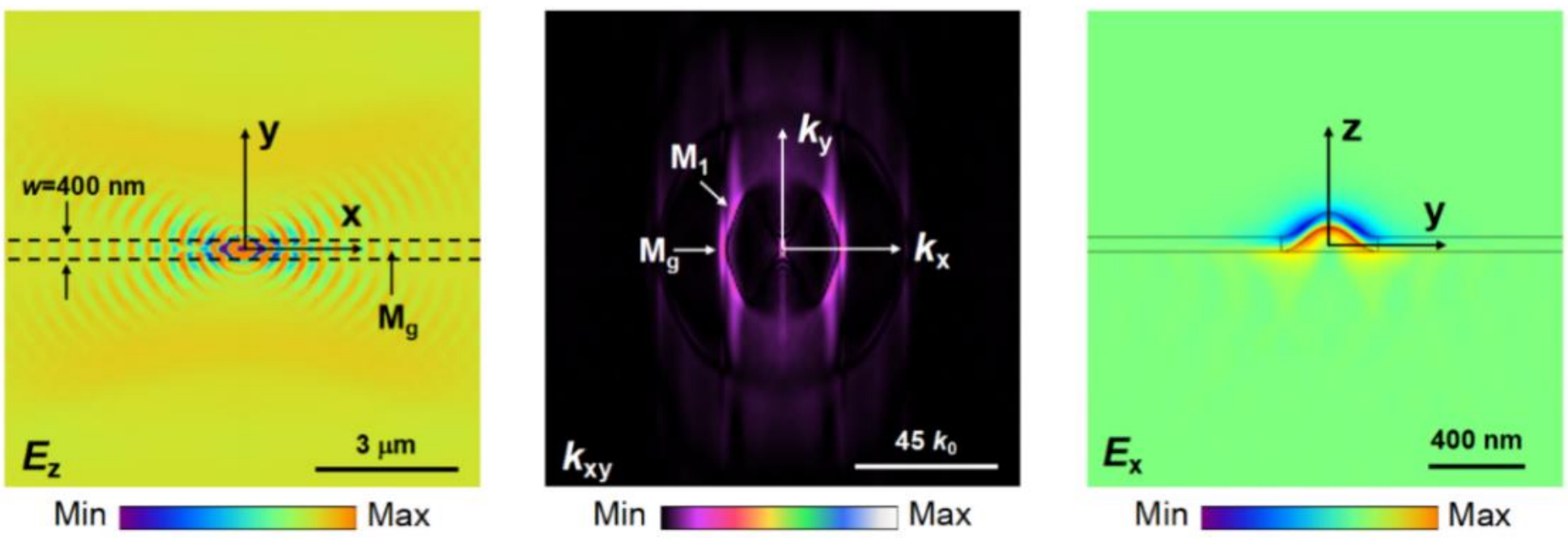

Fig. R6 | Guided mode in a substrate-attached wrinkle. Simulation of a substrate-attached wrinkle. The structure supports an M_g mode with a wavevector of ~$18.9k_0$, a value comparable to that of the planar M_1 mode.

3. What is the blue solid line in Figure 1a?

We thank the reviewer for pointing this out. The blue solid line in the original Fig.1a (Fig. R7a) was intended to visually separate the two stages of the wrinkle formation process—pre-buckling and post-buckling. In the revised manuscript, we have removed this line to simplify the figure and improve clarity (Fig. R7b).

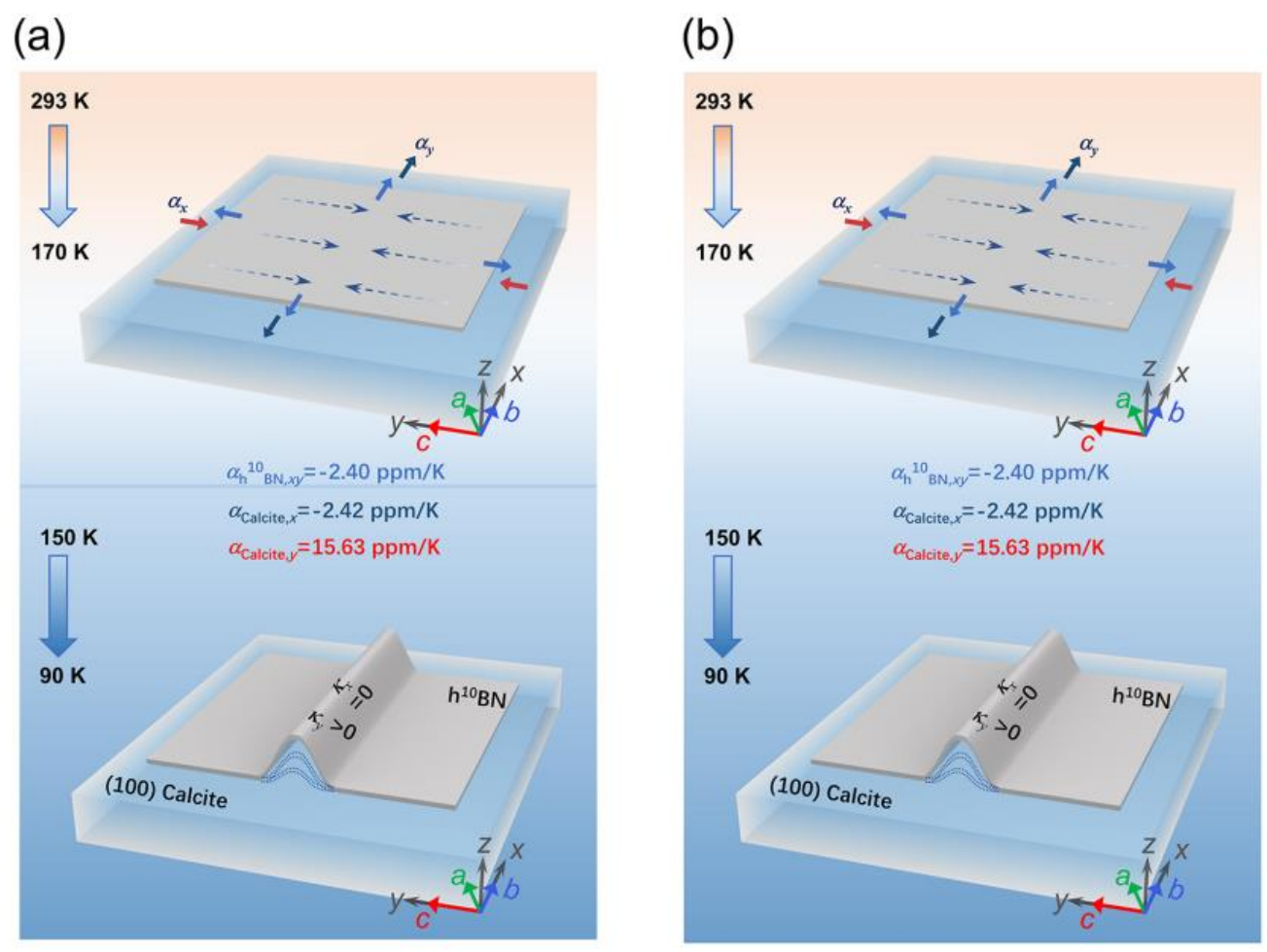

Fig. R7 | Diagram of the proposed scheme for preparing buckling vdW surfaces using the uniaxial thermal stress at the interface between a h^{10}BN nanosheet and a (100)-cut calcite substrate upon cooling. (a) Original version with a blue solid line; (b) revised version without the demarcation line.

To resolve the concerns of the reviewer, we have made the following changes in the revised manuscript:

(1) Replaced **Fig. 1a** with **Fig. R7b**.

4. How do authors justify the permittivity value of h^{10}BN? Strain and temperature may shift phonon resonances in the Reststrahlen bands.

We thank the reviewer for raising this important point. In our initial submission, the dielectric function of h^{10}BN was adopted from room-temperature data (*Nature Materials, 2018, 17, 152*) without accounting for strain and temperature effects. Our Raman data (Fig. 1b) confirm that strain is negligible, as it is fully released after wrinkle formation. However, temperature effects do require careful consideration, and we have now incorporated them into our simulations as follows.

Temperature-dependent dielectric correction: We adjust the dielectric function by shifting the frequency of the dominant E_{2g} phonon using the experimentally measured temperature coefficient k_{post}=-0.01892 cm^{-1}/K (Fig. 1b). The E_{2g} mode is chosen because it is the dominant Raman-active phonon in h^{10}BN, and its frequency

shift directly reflects the intrinsic lattice response in the strain-released regime. At our operating frequency of 1460 cm⁻¹, the dielectric response is governed primarily by the E_{2g} phonon; contributions from other modes (e.g., the infrared-active A_{2u} mode near 785 cm⁻¹) are negligible due to the large spectral separation.

Updated simulations: Using this temperature-corrected dielectric function, we systematically updated all electromagnetic simulations in the revised manuscript. The recalculated wavevector for the guided M_g mode is 24.58k_0 at 90 K (Fig. R8d), compared to 25.65k_0 obtained with the room-temperature parameters (Fig. R8c)—a relative difference of only ~4.3%.

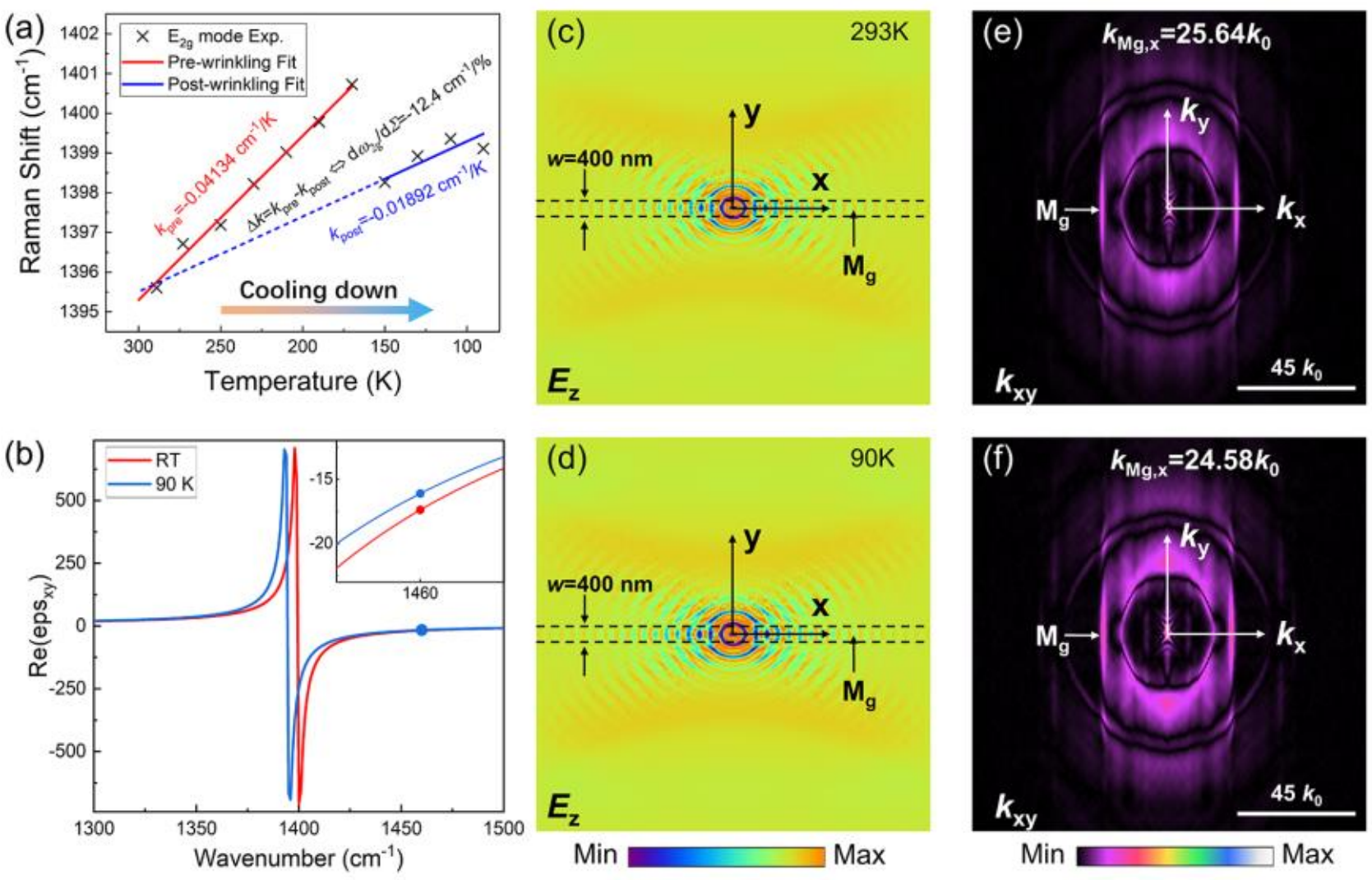

Fig. R8 | Temperature correction of the dielectric function and its effect on simulated polariton guiding. (a) Monitoring the wrinkling process of a h^{10}BN nanosheet using variable-temperature Raman spectroscopy. (b) Real part of the in-plane dielectric function of h^{10}BN at 90 K (blue line) after temperature correction, compared with the original RT curve (red line). (c, d) Original simulation results using RT dielectric parameters: (c) real-space image and (d) corresponding Fourier-transform map of the guided M_g mode at 1460 cm⁻¹. (e, f) Updated simulation results using the temperature-corrected dielectric function at 90 K: (e) real-space image and (f) Fourier-transform map.

To resolve the concerns of the reviewer, we have made the following changes in the revised manuscript:

(1) added the following explanation "Based on the variable-temperature Raman data (Fig. 1b), the accumulated strain is fully released after wrinkle formation. Therefore, for the simulations and cryo-SNOM experiments conducted at 90 K (post-buckling regime), only the temperature dependence of the dielectric function is considered. This dependence is incorporated by correcting the frequency of the dominant E_{2g} phonon using the experimentally measured coefficient k_{post}=−0.01892 cm⁻¹/K, which shifts the E_{2g} frequency to 1399.4 cm⁻¹ at 90 K. The E_{2g} mode is chosen because it is the dominant Raman-active phonon in h^{10}BN and its frequency shift directly reflects the intrinsic thermal response in the strain-released state. At the operational frequency of 1460 cm⁻¹,

the dielectric response is governed primarily by the E_{2g} phonon, while contributions from other modes (e.g., the A_{2u} phonon near 785 cm^{-1}) are negligible due to the large spectral separation." to the **1.1 The dielectric function of calcite and *h*^{10}BN** of the **Supplementary information** (**page 3**);

(2) recalculated all electromagnetic simulations based on the temperature-corrected dielectric function for *h*^{10}BN at 90 K. The following updates have been made to the main text:

Revised figures: **Fig. 2**, **Supplementary Fig. 5**, and **Supplementary Fig. 7** have been updated accordingly.

Updated wavevectors: The extracted wavevectors of the polariton modes have been adjusted as follows: k_{M1x}=17.73k_0 and k_{M2x}=38.97k_0 **(page 6, paragraph 3);** k_{Mg}=24.58k_0, k_{M1x}=17.73k_0, and the freestanding reference value 24.65k_0 (**page 7, paragraph 1)**; and as k_{M1x}=17.73 k_0 and k_{Mgx}=23.49k_0 **(page 8, paragraph 1).**

5. Since a new guided mode is claimed, it will be useful to plot representative s-SNOM line profiles in Fig. 3 and compare them with profiles from an unwrinkled region.

We thank the reviewer for this constructive suggestion. We agree that direct line-profile comparison provides valuable evidence for the new guided mode.

Challenge: The raw near-field image (Fig. 3e) contains superimposed signals from substrate-coupled planar modes ($M_{1,h}$ and $M_{1,t}$) and the wrinkle-guided mode (M_g), making direct profile comparison difficult.

Solution: To isolate M_g, we applied spatial frequency filtering to the experimental data—a method we validated on simulations (Fig. R9a, b) before applying to experiments. After filtering out the planar mode components from Fig. 3e, we extracted line profiles across the wrinkle (Fig. R9c) and a flat region (Fig. 3b). The wrinkle profile exhibits a single periodic fringe with a spatial period of 287 nm, corresponding to the M_g mode wavevector ($k_{Mg,x}$=23.82k_0). In contrast, the flat-region profile shows two distinct periods, corresponding to the substrate-coupled $M_{1,h}$ and $M_{1,t}$ modes. This direct comparison provides clear visual and quantitative evidence that the wrinkle supports a new, confined guided mode distinct from planar polaritons.

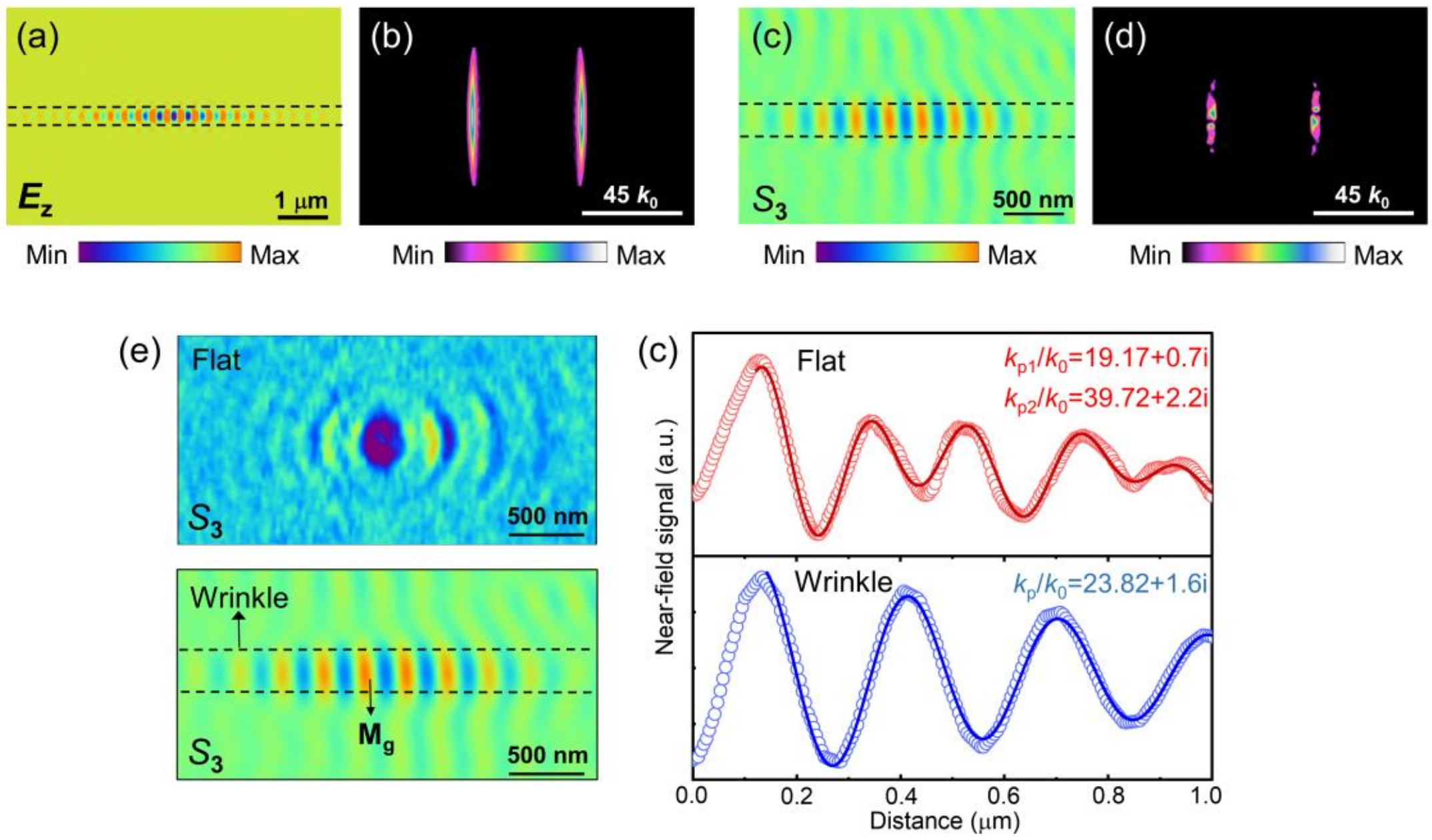

Fig. R9 | Isolation of the wrinkle-guided polariton mode via spatial frequency filtering. (a) Filtered real-space image of the simulated electric field after applying a spatial frequency filter to the Fourier transform in Fig. 2d. (b) Corresponding filtered momentum-space map of the simulation. (c) Experimentally filtered near-field image revealing guided wavefronts along the wrinkle. (d) Filtered real-space near-field amplitude obtained from experimental cryo-SNOM data (Fig. 3f) by removing the substrate-coupled $M_{1,h}$ and $M_{1,t}$ components. (e) Line profiles across the wrinkle (blue) and a flat region (red).

To resolve the concerns of the reviewer, we have made the following changes in the revised manuscript:

(1) added the following explanation "(clearly revealed in the filtered real-space image where M_1 and M_2 components are removed, Supplementary Fig. 6)" to the main text (**page 6, paragraph 5**);

(2) added **Fig. R9a, b** to the Supplementary Information as **Supplementary Fig. 6**;

(3) replaced **Supplementary Fig. 7c, d** with **Fig. R9c, d;**

(4) added **Fig. R9e** to the Supplementary Information as **Supplementary Fig. 8**;

(5) added the following explanation "By applying 2D FFT filtering to Fig. 3e, the resulting real-space near-field image (Supplementary Fig. 8b) clearly identifies the M_g mode. The line profile across the wrinkle shows a single periodic fringe corresponding to this mode, with a fitted quality factor of Q=Re(k_p)/Im(k_p)=14.9, while the profile from the flat region exhibits two distinct periods associated with the $M_{1,h}$ and $M_{1,t}$ modes (Supplementary Fig. 8c)." to the main text (**page 8, paragraph 1**).

6. What happens when the sample returns to room temperature? Upon re-cooling below the critical temperature, do the same wrinkles re-form? How about the yield and repeatability?

We thank the reviewer for these valuable questions. Our study shows that the wrinkling process is reversible and repeatable across multiple cooling-heating cycles.

(1) **Reversibility:** When the sample in Fig. 3d returns to room temperature, the wrinkles vanish and the h^{10}BN film reverts to a flat state, as shown in Fig. R10. Correspondingly, the guided polariton mode disappears, and the optical response returns to that of planar modes.

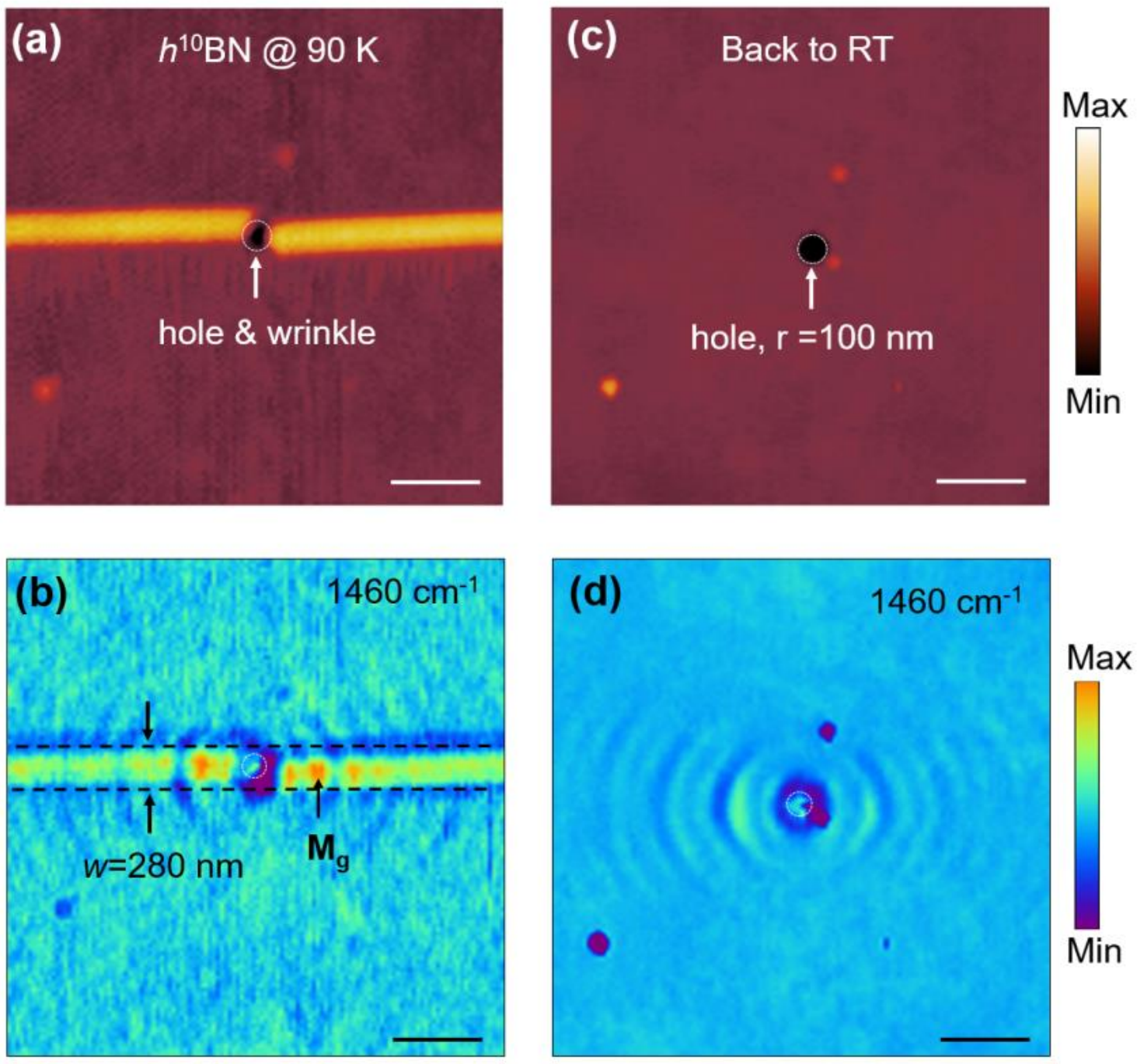

Fig. R10 | Real-space imaging of polaritons in h^{10}BN/calcite heterostructures: from wrinkled to recovered planar states. (a) AFM topography of a 60-nm-thick h^{10}BN/calcite heterostructure at 90 K, exhibiting a pronounced nano-wrinkle. (b) Near-field image of polaritons on the wrinkled h^{10}BN nanosheet in (a), showing guided propagation along the wrinkle axis. (c) AFM topography of the same heterostructure shown in (d) after returning to room temperature (~300 K), showing complete disappearance of the wrinkle and recovery of a flat surface. (d) Near-field image of polaritons in the recovered planar region (c), with an excitation frequency of 1460 cm^{-1}. (a-d) share the same scale bar: 500 nm.

(2) **Deterministic re-formation and repeatability**: To systematically demonstrate the reversibility and repeatability of the wrinkle formation process, we performed multiple cooling-heating cycles on a graphite/calcite heterostructure (Fig. R11). AFM images from four thermal cycles (Cycle-1 to Cycle-4, Fig. R11a) show that wrinkles re-form at nearly identical locations, demonstrating spatial repeatability governed by interfacial properties. Statistical analysis (Fig. R11b) further reveals that

wrinkle dimensions (height and width) are tunable and repeatable under controlled cycling conditions: a larger temperature drop (Cycle-2, pre-heated to 473 K before cooling) produces a larger wrinkle compared to a moderate drop (Cycle-1, RT cooling to 88.5 K). Following a large-strain cycle, partial delamination can lead to increased dimensions (Cycle-3, RT cooling to 90 K). When the same thermal swing is reapplied (Cycle-4), the wrinkle dimensions closely match those of Cycle-2 (within ~5% variation), confirming that reproducible morphology is achieved with a consistent thermal history.

Therefore, the wrinkle formation process is reversible, deterministic in location, and repeatable in geometry when the thermal cycle is controlled, underscoring the potential of the thermal-buckling approach for creating reconfigurable nanophotonic elements.

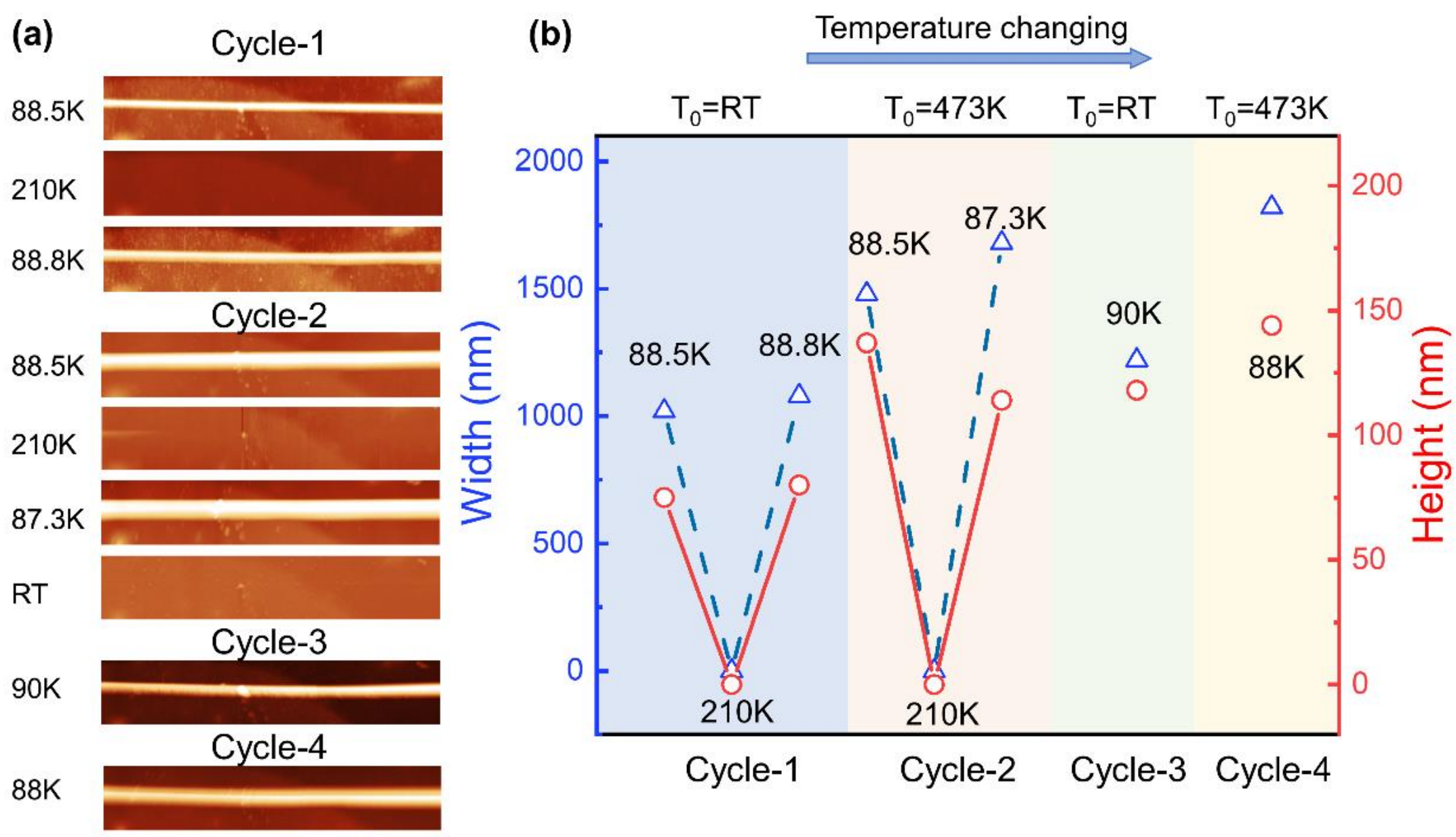

Figure R11. Reversibility and tunability of nano-wrinkles under cyclic thermal loading. (a) Atomic force microscopy (AFM) topography images of a wrinkled graphite/calcite heterostructure across four thermal cycles (Cycle-1 to Cycle-4), demonstrating deterministic wrinkle re-formation at nearly identical locations after repeated cooling below and warming above the critical temperature. (b) Statistical analysis of wrinkle dimensions (height and width) extracted from the cycles in a. The height and width are tunable and repeatable under controlled thermal conditions: a larger temperature drop (Cycle-2, pre-heated to 473 K before cooling) produces a significantly larger wrinkle compared to a moderate drop (Cycle-1, RT cooling to 88.5K). Following a large-strain cycle, partial delamination can lead to increased dimensions (Cycle-3, RT cooling to 90K). When the same thermal swing is reapplied (Cycle-4), the wrinkle dimensions closely match those of Cycle-2 (within ~5% variation), confirming that reproducible morphology is achieved with a consistent thermal history.

To resolve the concerns of the reviewer, we have made the following changes in the revised manuscript:

(1) added the following explanation "To systematically show the reversibility and repeatability of the wrinkle formation process, multiple cooling-heating cycles are performed on a graphite/calcite heterostructure. Variable-temperature AFM imaging (Supplementary Fig. 4a) demonstrates the reversibility: wrinkles vanish as the film returns to a flat state upon warming above the critical temperature and re-form at nearly identical locations upon subsequent re-cooling. The wrinkles deterministically repeat their spatial positions across four thermal cycles (Cycle-1 to Cycle-4), confirming that nucleation is governed by interfacial properties. Furthermore, the wrinkle geometry (height and width) is both tunable and reproducible under controlled thermal conditions. A larger temperature drop (e.g., cooling from 473 K in Cycle-2, Supplementary Fig. 4b) produces a larger wrinkle, while re-applying the same thermal history (Cycle-4) yields dimensions that closely match those of a prior cycle (Cycle-2), with variations within ~5%. These findings establish that the thermal-buckling process is reversible, spatially deterministic, and geometrically controllable, highlighting its potential for creating reconfigurable nanophotonic elements." to the main text (**page 6, paragraph 2**);

(2) added the following explanation "**Controllable and Repeatable Geometry**: The wrinkle dimensions (height and width) are tunable and repeatable under controlled cycling conditions. Multiple cooling-heating cycles are conducted on a graphite/calcite heterostructure, and the morphology of the wrinkled region is characterized, as summarized in Supplementary Fig. 4a (AFM topographies) and Fig. S4b (statistical analysis of wrinkle dimensions).

Cycle-1: Cooling from room temperature to 88.5 K, warming to 210 K, and re-cooling to 88.8 K.

Cycle-2: Involved a larger thermal swing. The sample was first pre-heated to 473 K, then cooled to 88.5 K, warmed to 210 K, and finally cooled to 87.3 K. The nearly twofold greater temperature drop compared to Cycle-1 resulted in a significantly larger wrinkle, confirming that dimensions can be amplified by increasing the thermal stress.

Cycle-3: Following Cycle-2, the sample was cooled again to 90 K. Due to the partial delamination and reduced interfacial adhesion from the previous large-strain cycle, the newly formed wrinkle was substantially larger than the one in Cycle-1.

Cycle-4: The sample was again heated to 473 K and cooled to 90 K. The resulting wrinkle dimensions were closely matched to those in Cycle-2 (~5% variation), demonstrating that consistent thermal variation leads to reproducible wrinkle morphology." to the **Supplementary Information** (**Page 7**);

(3) added **Fig. R11** to the Supplementary Information as **Supplementary Fig. 4**.

7. Authors should also mention more details on how they fabricate those nano-holes on the samples. Since the title of the manuscript is programmable, the authors should also highlight this.

We thank the reviewer for these suggestions. We have now added detailed fabrication steps in **Methods** and further highlighted the **programmability** of our approach throughout the manuscript.

(1) **Nano-hole fabrication details**: The complete process for fabricating nano-hole arrays is as follows. First, h^{10}BN nanosheets were mechanically exfoliated from natural crystals onto SiO_2 (300 nm)/Si substrates. Subsequently, to define programmable nucleation sites for wrinkles, periodic arrays of nano-holes were patterned into the h^{10}BN flakes using 100 kV electron-beam lithography (EBL; Vistec 5000+ES). Prior to exposure, the flakes were spin-coated with a ~350 nm thick layer of polymethyl methacrylate (PMMA) 950K resist. After development (60 s) and an isopropanol (IPA) rinse, patterned holes were transferred into the h^{10}BN layer via reactive ion etching (RIE; Etchlab 200). Residual PMMA was removed by soaking in hot acetone at 80 °C for 30 min, followed by a gentle IPA rinse and N_2 drying.

Following nanopatterning, the h^{10}BN flakes were transferred onto (100)-cut calcite substrates using a PMMA-assisted wet transfer method. A PMMA 950K layer (~350 nm) was spin-coated onto the SiO_2/Si substrate and baked. The PMMA/h^{10}BN stack was released by etching the underlying SiO_2 in 1 M KOH and then scooped onto the calcite substrate. After transfer, the PMMA support layer was removed by soaking in acetone at 80 °C for >1 h. Residual contaminants were further cleaned in PG remover (60 min) and warm acetone (30 min), followed by a final IPA rinse and N_2 drying.

(2) **Highlighting programmability**: We clarify that the programmability of our approach is demonstrated through three aspects:

- **Spatial programmability:** Pre-patterned nano-holes act as spatially programmable nucleation sites, translating a designed 2D hole pattern into a 1D wrinkle waveguide (Supplementary Fig. 2).
- **Geometric programmability:** Wrinkle height decreases linearly with temperature (Supplementary Fig. 3), enabling programmable control over wrinkle geometry via thermal tuning.
- **Dispersion programmability:** The guided mode wavevector depends strongly on wrinkle geometry (Supplementary Fig. 9g), and because height is thermally tunable, the polariton dispersion—and thus the waveguide optical function—becomes programmable in situ.

To resolve the concerns of the reviewer, we have made the following changes in the revised manuscript:

(1) added the following explanation in **Methods** "Fabricating nano-hole arrays: First, h^{10}BN nanosheets were mechanically exfoliated from natural crystals onto SiO_2 (300 nm)/Si substrates. Subsequently, to define programmable nucleation sites for wrinkles, periodic arrays of nano-holes were patterned into the h^{10}BN flakes using 100 kV electron-beam lithography (EBL; Vistec 5000+ES). Prior to exposure, the flakes were spin-coated with a ~350 nm thick layer of polymethyl methacrylate (PMMA) 950K

resist. After development (60 s) and an isopropanol (IPA) rinse, patterned holes were transferred into the $h^{10}BN$ layer via reactive ion etching (RIE; Etchlab 200). Residual PMMA was removed by soaking in hot acetone at 80 °C for 30 min, followed by a gentle IPA rinse and N_2 drying.

Following nanopatterning, the $h^{10}BN$ flakes were transferred onto (100)-cut calcite substrates using a PMMA-assisted wet transfer method. A PMMA 950K layer (~350 nm) was spin-coated onto the SiO_2/Si substrate and baked. The PMMA/$h^{10}BN$ stack was released by etching the underlying SiO_2 in 1 M KOH and then scooped onto the calcite substrate. After transfer, the PMMA support layer was removed by soaking in acetone at 80 °C for >1 h. Residual contaminants were further cleaned in PG remover (60 min) and warm acetone (30 min), followed by a final IPA rinse and N_2 drying." (**Page 17, Paragraph 2-3**);

(2) added the following explanation "This linear dependence of wrinkles *h* enables programmable control over wrinkle geometry via thermal tuning." to the main text (**Page 5, Paragraph 3**);

(3) added the following explanation "indicating the programmable dispersion of the guided polariton mode—and hence the waveguide's optical function—through wrinkle geometry" to the main text (**Page 9, Paragraph 1**).

List of changes

(in the order of appearance in the revised manuscript)

(1) revised the wording in the **Abstract**: “The intrinsic planarity of two-dimensional (2D) van der Waals (vdW) polaritonic media inherently limits the lateral confinement of vdW polaritons. Here, we transcend this geometric limit by introducing a deterministic strategy to engineer non-planar nano-wrinkle waveguides in hexagonal boron nitride (*h*BN) via controlled thermal buckling.” (**Page 1**);
(2) revised the last sentence of the **Abstract** “More broadly, our approach establishes a universal route for the geometric control of light and heat in two-dimensional materials.” (**Page 1**);
(3) modified the explanation “The emergence of van der Waals (vdW) materials enables low-loss, tunable polaritons that surpass conventional systems based on metals or polar crystals in both propagation length and spectral flexibility.” in the main text (**Page 2, paragraph 1**);
(4) highlighted the explanation “$\alpha_{\perp}$ and $\alpha_{//}$ of calcite are found to be -2.42 ppm/K and 15.63 ppm/K respectively within the temperature range from 100 K to 400 K. Noting that the average in-plane TEC of *h*BN in the same temperature interval is nearly identical to $\alpha_{\perp}$ of calcite (for natural *h*BN α_{hBN}=-2.48 ppm/K and for isotopically purified h^{10}BN $\alpha_{h^{10}BN}$=-2.40 ppm/K)” in the main text (**Page 3, paragraph 2**);

(5) modified the explanation “The net result is a **highly directional uniaxial compressive stress** acting on h^{10}BN indicated schematically in Fig. 1a, which induces a compression strain Σ within the nanosheet along the y axis, **forcing the h^{10}BN to buckle solely perpendicular to the stress field**.” in the main text (**Page 4, paragraph 1**);

(6) highlighted the explanation “To spatially program wrinkle nucleation, we perforate h^{10}BN nanosheets and use the resultant nano-holes (see Methods for fabrication details) to intensify local thermal stress and weaken at the same time interfacial cohesion in the vicinity of these holes” and “the neighboring regions of nano-holes preferred locations for h^{10}BN wrinkling.” in the main text (**Page 5, paragraph 2**);

(7) added the explanation “This linear dependence of wrinkles h enables programmable control over wrinkle geometry via thermal tuning.” to the main text (**Page 5, paragraph 3**);

(8) added the following explanation “To systematically show the reversibility and repeatability of the wrinkle formation process, multiple cooling-heating cycles are performed on a graphite/calcite heterostructure. Variable-temperature AFM imaging (Supplementary Fig. 4a) demonstrates the reversibility: wrinkles vanish as the film returns to a flat state upon warming above the critical temperature and re-form at nearly identical locations upon subsequent re-cooling. The wrinkles deterministically repeat their spatial positions across four thermal cycles (Cycle-1 to Cycle-4), confirming that nucleation is governed by interfacial properties. Furthermore, the wrinkle geometry (height and width) is both tunable and reproducible under controlled thermal conditions.

A larger temperature drop (e.g., cooling from 473 K in Cycle-2, Supplementary Fig. 4b) produces a larger wrinkle, while re-applying the same thermal history (Cycle-4) yields dimensions that closely match those of a prior cycle (Cycle-2), with variations within ~5%. These findings establish that the thermal-buckling process is reversible, spatially deterministic, and geometrically controllable, highlighting its potential for creating reconfigurable nanophotonic elements." to the main text (**page 6, paragraph 2**);

(9) changed the Supplementary Figure number from 4 to 5 in the main text (**page 6, paragraph 3**);

(10) corrected the wavevectors in the *x* direction (real parts): k_{M1x} changed from 18.17k_0 to 17.73k_0; k_{M2x} changed from 39.85k_0 to 38.97k_0 in the main text (**page 6, paragraph 3**);

(11) added the following explanation "(clearly revealed in the filtered real-space image where M_1 and M_2 components are removed, Supplementary Fig. 6)" to the main text (**page 7, paragraph 1**);

(12) corrected the wavevectors k_{Mgx} and k_{M1x} to 24.58k_0 and 17.73k_0 respectively, and that of the wrinkle-induced mode in the freestanding case to 24.65k_0 in the main text (**page 7, paragraph 1**);

(13) corrected the wavevectors k_{Mgx} and k_{M1x} to 23.49k_0 and 17.73k_0 respectively in the main text (**page 8, paragraph 1**);

(14) changed the Supplementary Figure number from 5 to 7 in the main text (**page 8, paragraph 1**);

(15) added the following explanation "By applying 2D FFT filtering to Fig. 3e, the resulting real-space near-field image (Supplementary Fig. 8b) clearly identifies the M_g mode. The line profile across the wrinkle shows a single periodic fringe corresponding to this mode, with a fitted quality factor of Q=Re(k_p)/Im(k_p)=14.9, while the profile from the flat region exhibits two distinct periods associated with the $M_{1,h}$ and $M_{1,t}$ modes (Supplementary Fig. 8c)." to the main text (**page 8, paragraph 1**);

(16) changed the Supplementary Figure number from 6 to 9 in the main text (**page 9, paragraph 1**);

(17) added the following explanation "indicating the programmable dispersion of the guided polariton mode—and hence the waveguide's optical function—through wrinkle geometry." to the main text (**page 9, paragraph 1**);

(18) revised the wording to "Crucially, when the wrinkle height exceeds the critical decoupling threshold (~50 nm), the dominant vertical heat-sinking pathway—direct interfacial conduction to the substrate—is effectively removed. The remaining heat dissipation occurs primarily via lateral conduction through the h^{10}BN film toward supported regions and then into the substrate, a pathway that is inherently limited by the series of thermal resistances (lateral spreading, finite interfacial conductance, and

substrate diffusion). This results in strongly suppressed substrate heat sinking for the suspended segments, as" in the main text (**page 11, paragraph 1**);

(19) changed the Supplementary Figure number from 7 to 10 in the main text (**page 11, paragraph 1**);

(20) modified the following explanation "**the polariton-mediated heat generation and the subsequent directional heat flow.** This synergy—between **wrinkle**-induced polariton confinement and suppressed thermal leakage **to the substrate**—establishes a robust, directional, and low-loss **low-thermal-leakage** mechanism for nanoscale heat routing." to the main text (**page 12, paragraph 2**);

(21) changed the Supplementary Figure number from 8 to 11 in the main text (**page 12, paragraph 3**);

(22) changed the Supplementary Figure number from 9 to 12 in the main text (**page 12, paragraph 4**);

(23) added the following explanation "Besides, this thermal expansion mismatch approach can likewise be applied to substrates with different symmetries. By altering the symmetry of the stress field, the wrinkle morphology can be actively controlled. For example, on an isotropic substrate like SiO_2, isotropic compression leads to the formation of a two-dimensional triple-symmetry wrinkle network, as observed in our experiments (Supplementary Fig. 13). This transition from 1D channels to 2D networks may enable interconnected polariton circuits or tunable photonic metasurfaces." to the main text (**page 13, paragraph 2**);

(24) added the following explanation in **Methods** "Fabricating nano-hole arrays: First, h^{10}BN nanosheets were mechanically exfoliated from natural crystals onto SiO_2 (300 nm)/Si substrates. Subsequently, to define programmable nucleation sites for wrinkles, periodic arrays of nano-holes were patterned into the h^{10}BN flakes using 100 kV electron-beam lithography (EBL; Vistec 5000+ES). Prior to exposure, the flakes were spin-coated with a ~350 nm thick layer of polymethyl methacrylate (PMMA) 950K resist. After development (60 s) and an isopropanol (IPA) rinse, patterned holes were transferred into the h^{10}BN layer via reactive ion etching (RIE; Etchlab 200). Residual PMMA was removed by soaking in hot acetone at 80 °C for 30 min, followed by a gentle IPA rinse and N_2 drying.

Following nanopatterning, the h^{10}BN flakes were transferred onto (100)-cut calcite substrates using a PMMA-assisted wet transfer method. A PMMA 950K layer (~350 nm) was spin-coated onto the SiO_2/Si substrate and baked. The PMMA/h^{10}BN stack was released by etching the underlying SiO_2 in 1 M KOH and then scooped onto the calcite substrate. After transfer, the PMMA support layer was removed by soaking in acetone at 80 °C for >1 h. Residual contaminants were further cleaned in PG remover (60 min) and warm acetone (30 min), followed by a final IPA rinse and N_2 drying." (**Page 17, Paragraph 2-3**);

(25) updated the **Data Availability Statement**: "The source data underlying the figures are provided as a Source Data file. Source data are provided with this paper." **(Page 18);**

(26) added the following explanation "Based on the variable-temperature Raman data (Fig. 1b), the accumulated strain is fully released after wrinkle formation. Therefore, for the simulations and cryo-SNOM experiments conducted at 90 K (post-buckling regime), only the temperature dependence of the dielectric function is considered. This dependence is incorporated by correcting the frequency of the dominant E_{2g} phonon using the experimentally measured coefficient k_{post}=−0.01892 cm^{-1}/K, which shifts the E_{2g} frequency to 1399.4 cm^{-1} at 90 K. The E_{2g} mode is chosen because it is the dominant Raman-active phonon in h^{10}BN and its frequency shift directly reflects the intrinsic thermal response in the strain-released state. At the operational frequency of 1460 cm^{-1}, the dielectric response is governed primarily by the E_{2g} phonon, while contributions from other modes (e.g., the A_{2u} phonon near 785 cm^{-1}) are negligible due to the large spectral separation." to the **1.1 The dielectric function of calcite and *h*^{10}BN** of the **Supplementary information** (**page 3**);

(27) changed the Supplementary Figure number from 4,5 to 5,7 in the **Supplementary information** (**page 3**);

(28) corrected the uniaxial permittivity of h^{10}BN used for the simulation in the **Supplementary Information** (**page 3**) to: $\varepsilon_{\parallel}$ =2.3387+1.5545e^{-4}i and $\varepsilon_{\perp}$ =-17.386+0.34103i;

(29) corrected the wavevectors for the structures with and without a calcite substrate to 24.58k_0 and 24.65k_0 in the **Supplementary information** (**page 4**).

(30) added the following explanation "**Controllable and Repeatable Geometry**: The wrinkle dimensions (height and width) are tunable and repeatable under controlled cycling conditions. Multiple cooling-heating cycles are conducted on a graphite/calcite heterostructure, and the morphology of the wrinkled region is characterized, as summarized in Supplementary Fig. 4a (AFM topographies) and Fig. S4b (statistical analysis of wrinkle dimensions).

Cycle-1: Cooling from room temperature to 88.5 K, warming to 210 K, and re-cooling to 88.8 K.

Cycle-2: Involved a larger thermal swing. The sample was first pre-heated to 473 K, then cooled to 88.5 K, warmed to 210 K, and finally cooled to 87.3 K. The nearly twofold greater temperature drop compared to Cycle-1 resulted in a significantly larger wrinkle, confirming that dimensions can be amplified by increasing the thermal stress.

Cycle-3: Following Cycle-2, the sample was cooled again to 90 K. Due to the partial delamination and reduced interfacial adhesion from the previous large-strain cycle, the newly formed wrinkle was substantially larger than the one in Cycle-1.

Cycle-4: The sample was again heated to 473 K and cooled to 90 K. The resulting wrinkle dimensions were closely matched to those in Cycle-2 (~5% variation), demonstrating that consistent thermal variation leads to reproducible wrinkle morphology." to the **Supplementary Information** (**Page 7**);

(31) added **Fig. R11** to the Supplementary Information as **Supplementary Fig. 4**.

(32) corrected the in-plane wavevectors to k_{M1}=2.13k_0, k_{M2}=23.27k_0, k_{M3}=44.42k_0, k_{Mg}=24.65k_0 in the Supplementary Information (**Page 8-9**);

(33) added **Fig. R2a, b** to the Supplementary Information as **Supplementary Fig. 6**;

(34) replaced **Supplementary Fig. 7c, d** with **Fig. R2c, d;**

(35) added **Fig. R2e** to the Supplementary Information as **Supplementary Fig. 8**;

(36) added **Fig. R4** to the Supplementary Information as **Supplementary Fig. 13**.

Point-by-Point Response to Reviewers (Revision 2)

We sincerely thank all three reviewers for their continued engagement with our manuscript. We are particularly grateful to Reviewer #3 for providing an independent assessment of the remaining concerns raised by Reviewer #1. We have carefully addressed all comments, and the manuscript has been revised accordingly. Our responses are provided below in blue, and all changes are highlighted in the revised manuscript.

Reviewer #1

General comment: After carefully reviewing the revised version and the response letter, I regret to say that my fundamental concerns regarding the novelty and the robustness of the results remain unresolved.

We thank the Reviewer for the continued careful evaluation. We maintain that the primary novelty of this work resides in the thermomechanical strategy itself — specifically, the deterministic creation, spatial programming, and reversible reconfiguration of nanoscale wrinkles via thermal tuning, which we harness as reconfigurable polariton waveguides in van der Waals materials. Crucially, this approach establishes geometric curvature as a new degree of freedom for polariton control, a capability hitherto unattained in this material family. We are encouraged that Reviewer #3, in an independent assessment, affirmed this view, stating that "a major strength... is the thermo-mechanical strategy itself... which is interesting and potentially generalizable," and explicitly noted that residual questions regarding mode confinement "do not undermine the overall significance of the work." We trust the following detailed responses address the remaining concerns satisfactorily.

(1) *The main concern is whether one can call the propagating waves within the wrinkle a "channelling" mode. Indeed, a guided wave with distinct wavevector is observed in the nano wrinkle, according to the FFT image. However, there is a large part of polariton energy leaks out to the substrate-supported region, which means the observed wave is actually a hybrid polariton mode with rather weak optical confinement.*

We thank the Reviewer for this pertinent observation. We concur that these points warrant rigorous clarification and address them systematically below. Specifically, we examine (i) whether the observed mode fulfills the physical criteria for a channeling mode, and (ii) the appropriate interpretation of its hybridization with substrate modes.

(i) Validation of Channeling Mode Characteristics

The defining criteria for a channeling mode are that it carries a distinct wavevector and dispersion relation relative to the surrounding slab modes, and that it propagates preferentially

along a defined geometric pathway. The wrinkle-induced M_g mode exhibits both characteristics. Its experimentally measured wavevector (k_{Mgx} = 23.82 k_0, closely matching the simulated value of 23.49 k_0) is clearly distinct from both the hole-edge-excited mode $M_{1,h}$ ($k_{M1,hx}$ = 19.17 k_0) and the tip-launched mode $M_{1,t}$ ($k_{M1,tx}$ = 39.72 k_0), as unambiguously resolved in the Fourier analysis (Fig. 3f). This mode propagates directionally along the wrinkle axis (Fig. 3e), with a quality factor $Q = \mathrm{Re}(k_p)/\mathrm{Im}(k_p) = 14.9$ extracted from the experimental line profile. Furthermore, the dispersion of M_g evolves continuously with wrinkle geometry — both height and width — as shown in **Fig. R1**. Above a critical wrinkle height of ~50 nm, the M_g mode exhibits nearly identical dispersion trends in both suspended and substrate-supported configurations, indicating that the guided mode is primarily governed by wrinkle geometry. We therefore maintain that the term "channeling mode" is physically appropriate for M_g.

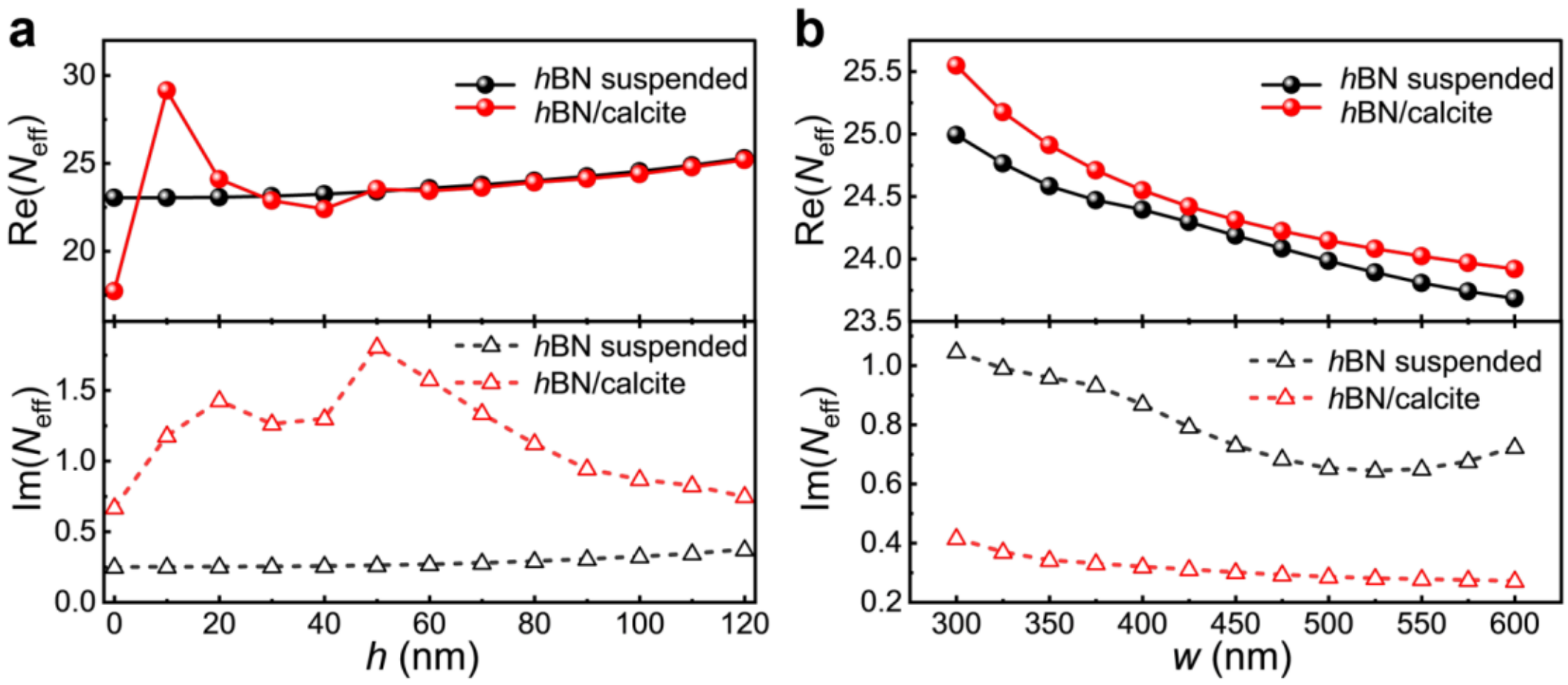

Figure R1 | Evolution of the wrinkle-guided M_g mode with wrinkle geometry. (a) Real (solid circles) and imaginary (open triangles) parts of the effective refractive index N_{eff} of the M_g mode as a function of wrinkle height h for suspended *h*BN (black) and *h*BN/calcite (red) structures. (b) Real (solid circles) and imaginary (open triangles) parts of N_{eff} of the M_g mode as a function of wrinkle width w for suspended *h*BN (black) and *h*BN/calcite (red) structures.

(ii) Hybrid Mode Characteristics and Tunability

The M_g mode in the present geometry indeed exhibits mode hybridization (**Fig. R2**b**)**. The partial energy leakage into the substrate-supported region (**Fig. R2c, d)** originates from momentum-space hybridization between the wrinkle-guided M_g branch and the second-order substrate-supported M_2 mode, as highlighted by the circled region in **Fig. R2**b.

We would like to suggest, however, that this hybridization should be viewed not as a deficiency but as a defining and exploitable feature of the wrinkle-on-substrate platform. It is precisely because of the coupling between the wrinkle-guided mode and the substrate-supported modes that the confinement of M_g becomes tunable (**Fig. R2**e). By controlling the

momentum-space overlap between the two branches, the degree of hybridization, and hence the modal confinement, can be systematically engineered. Decreasing the h^{10}BN thickness from 60 nm to 30 nm shifts M_2 spectrally away from M_g, substantially suppressing the hybridization and increasing the confinement ratio η_{Mg} ($\eta = \frac{P_{\mathrm{wrinkle}}}{P_{\mathrm{total}}} \times 100\% = \frac{\iint_{\mathrm{wrinkle}} S_x(y,z)dydz}{\iint_{\mathrm{A}} S_x(y,z)dydz} \times 100\%$) to 93.1% (**Fig. R2**f).

Taken together, these results reframe the hybrid character of M_g as a tunable property: the substrate introduces an additional mode branch that hybridizes with the wrinkle-guided channel, and the strength of this hybridization is a designable parameter controlled by geometry and material thickness.

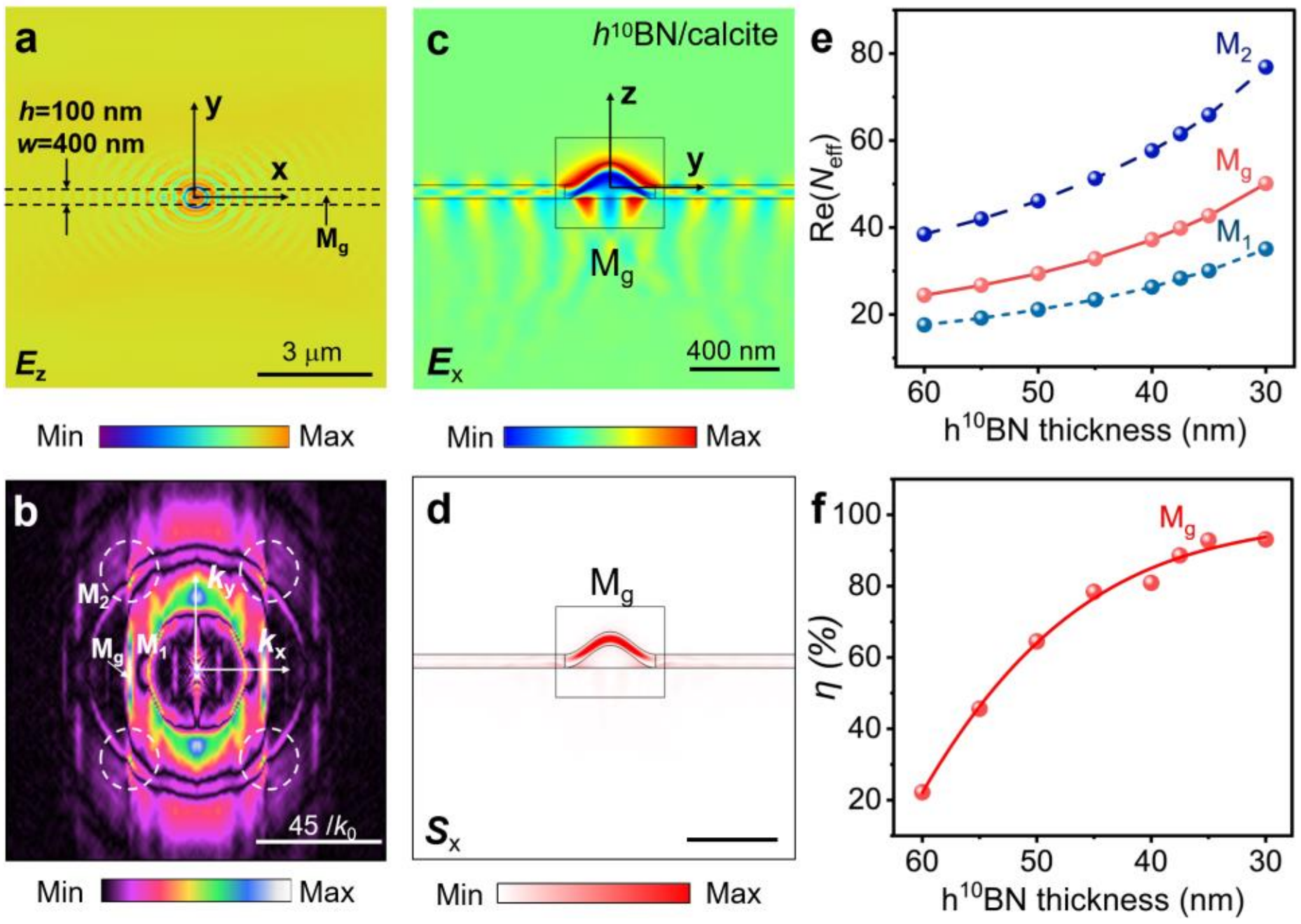

Figure R2 | Power-flow confinement and thickness-dependent tunability of wrinkle-guided polariton modes in *h*^{10}BN. (a) Simulated wavefronts at the surface of a wrinkled h^{10}BN nanosheet, the width of the wrinkle w is 400 nm and the height h is 100 nm (b) Fourier transform of (a), M_g is a wrinkle-guided mode. The circled region highlights the overlap between the wrinkle-guided M_g mode and the substrate-supported M_2 mode. (c) Cross-sectional mode profile of M_g, the component of the optical field E_x is shown. (d) Cross-sectional $\boldsymbol{S}_x$ distribution of the M_g mode. (e) Real part of the effective refractive index Re(N_{eff}) of the M_g wrinkle-guided mode as a function of h^{10}BN thickness. (f) Confinement ratio η_{Mg} as a function of h^{10}BN thickness.

To resolve the concerns of the reviewer, we have made the following changes in the revised manuscript:

(1) revised the wording in the **Abstract**: "Cryogenic near-field imaging and simulations reveal a wrinkle-guided polariton mode with a wavevector and dispersion governed by the wrinkle

geometry and distinct from substrate-supported branches. Their hybridization allows precise tuning of modal confinement via *h*BN thickness variation."(**Page 1**);

(2) revised the wording: "Wrinkling of the h^{10}BN nanosheet (400 nm width and 100 nm height) breaks the in-plane symmetry of the heterostructure, leading to a redistribution of polariton wavefronts in real space (Fig. 2c) and a reconfiguration of the isofrequency contours (Fig. 2d). This geometric perturbation gives rise to a distinct M_g mode (clearly resolved in filtered real-space images with M_1 and M_2 components filtered out, Supplementary Fig. 6), which propagates predominantly along the $\pm x$ direction with an in-plane wavevector of $24.58k_0$, clearly separated from the substrate-supported M_1 and M_2 modes. Cross-sectional field profiling confirms that the M_g mode is localized within the suspended wrinkle region (Fig. 2e), consistent with a geometry-confined propagation channel. The guided nature of the M_g mode is reflected in its systematic dependence on wrinkle geometry, with both the real and imaginary parts of the effective index N_{eff} evolving continuously as functions of wrinkle height h and width w (Fig. 2f and Supplementary Fig. 7). Notably, for $h > 50$ nm, the M_g mode exhibits nearly identical dispersion trends in both suspended and substrate-supported configurations. Collectively, these observations—its distinct wavevector, characteristic isofrequency contour, and geometry-dependent dispersion—support the interpretation of M_g as a wrinkle-guided channeling mode." in the main text (**page 6, paragraph 3**);

(3) added **Fig. R1** to the Supplementary Information as **Supplementary Fig. 7**.

(4) added the following explanation "While the existence of the M_g mode is determined by the wrinkle geometry, its confinement is influenced by coupling to substrate-supported modes. As shown in **Fig. 2**d, the isofrequency contours of M_g partially overlap with those of the second-order substrate-supported mode M_2, inducing hybridization between the two branches. This hybridization redistributes polariton energy and thereby provides a route for engineering modal confinement through h^{10}BN thickness tuning. As shown in Supplementary Fig. 8, reducing the h^{10}BN thickness from 60 nm to 30 nm shifts the M_g and M_2 modes to larger wavevectors and decreases the overlap of their isofrequency contours, thereby weakening mode hybridization and increasing the M_g mode confinement ratio η to 93.1%." to the main text (**page 7, paragraph 2**);

(5) added the following explanation "The confinement ratio is defined as:

$$\eta = \frac{P_{\text{wrinkle}}}{P_{\text{total}}} \times 100\% = \frac{\iint_{\text{wrinkle}} S_x(y,z)dydz}{\iint_{\text{A}} S_x(y,z)dydz} \times 100\%$$

where S_x is the time-averaged Poynting vector component along the propagation direction, P_{wrinkle} is the power flowing within the wrinkle region (as indicated by the rectangular region in Supplementary Fig. 8b), and P_{total} is the total power crossing the entire transverse section." to the **Supplementary Information (page 11);**

(6) added **Figure R2c-f** to the Supplementary Information as **Supplementary Fig. 8**.

(2) & (3) *I understand that scanning near-field optical imaging in cryogenic environment is tricky, however, the quality (i.e. the low signal-to-noise) of the experimental s-SNOM images can lead to the loss of key information, especially the wave propagates outside the wrinkle.*

Along with point 2, the anisotropy maybe enhanced by the detection scheme of s-SNOM. For example, Fig.3b shows an anisotropic propagation of polaritons within a flat h-BN slab, which in fact should exhibit an isotropic wavefronts. This detection artifact indicated that the anisotropy of the claimed channelling polaritons (Fig.3e, the key experimental evidence) may also be strongly overestimated.

We thank the Reviewer for raising this important point regarding the reliability of cryogenic s-SNOM measurements. We agree that near-field imaging under cryogenic conditions generally exhibits lower signal-to-noise ratios than room-temperature measurements, primarily due to exacerbated thermomechanical drift and degraded optical coupling.

However, the anisotropic polariton wavefront in Fig. 3b does not stem from information loss outside the wrinkle caused by low SNR; rather, it arises from the intrinsic optical anisotropy of the (100)-cut calcite substrate. The calcite substrate exhibits an in-plane hyperbolic optical response at 1460 cm^{-1} ($\varepsilon_x = -5.02 + 0.79i$ and $\varepsilon_y = 2.34$), which modifies the in-plane polariton dispersion of h^{10}BN and forbids propagation within specific angular ranges relative to the y-axis. Consequently, anisotropic wavefronts are expected even in a nominally flat h^{10}BN slab. The experimentally observed anisotropy is consistent with the full-wave simulations in Fig. 2a and the corresponding Fourier analysis in Fig. 2b.

Reviewer #3

The authors have adequately addressed my concerns through sufficient revision and the addition of important new experimental and simulation results.

Regarding the remaining concerns raised by referee #1:

Reviewer #1 raises an important concern regarding whether the observed mode should be described as a true "channeling" mode. I agree that this is important, especially since the wrinkle is narrow and cryogenic near-field imaging is experimentally challenging, which likely limits the SNR compared with room-temperature s-SNOM measurements.

That said, I do think the manuscript already provides meaningful evidence for a wrinkle-induced polariton mode. In particular, the FFT analysis and FEM simulations support the existence of a mode with a distinct wavevector from the normal substrate-supported mode, and the simulated field profiles indicate localization around the wrinkle region. In addition, the waves observed outside the wrinkle should not simply be interpreted as leakage from the wrinkle-guided mode, since the nano-hole acts as a dipole polariton launcher and will inevitably excite the conventional mode around the hole as well. If one takes a look at their Supplementary Fig. 9. The guided mode at wrinkle strictures is actually distinguishable from the conventional mode, and its dispersion deviates accordingly.

I would however encourage the authors to provide more quantitative analysis of the mode, such as decay length, and a clearer comparison between propagation inside and outside the wrinkle. If available, additional data showing how the mode evolves with wrinkle geometry, including wrinkle width and how it compares to the suspended $h^{10}BN$ mode, would further support the waveguide interpretation. Otherwise, the claim of a tightly confined "channeling" mode should be stated somewhat more cautiously.

In my view, this issue does not undermine the overall significance of the work. A major strength of the manuscript is the thermo-mechanical strategy itself, where one can create and reversibly control nano-wrinkle structures through thermal tuning, which is interesting and potentially generalizable to other vdW materials, and possibly to other thin-film material systems.

We thank the Reviewer for the careful and balanced assessment, and for the constructive suggestions that have helped us strengthen the manuscript. We agree that the central contribution of this work is the thermomechanical strategy for creating and reconfiguring nanoscale wrinkle structures, and that the existing FFT analysis and simulations already provide meaningful evidence for a wrinkle-induced polariton mode with a distinct wavevector and dispersion. Following the Reviewer's suggestions, we have added quantitative characterization of the propagation characteristics and a systematic investigation of mode evolution with wrinkle geometry, providing additional support for the waveguide interpretation.

1. Decay length and quantitative inside-vs-outside comparison

Based on the experimental line profiles shown in Supplementary Fig. 10c, we extract the

propagation characteristics of both the wrinkle-guided and substrate-supported modes. For the wrinkle region, the fitted quality factor is Q = Re(k_p)/Im(k_p) = 14.9, corresponding to a 1/e amplitude decay length of $L=\lambda_{Mg}\times Q/(2\pi)$=0.68 μm; for the flat region, two distinct fringe periods are resolved, corresponding to $M_{1,h}$ mode (Q=27.4, L=1.56 μm) and $M_{1,t}$ mode (Q=18.1, L=0.50 μm). These results confirm that the M_g mode inside the wrinkle carries a distinct wavevector and propagation characteristics from the substrate-supported modes.

2. Mode evolution with wrinkle geometry

To assess how the M_g mode evolves with wrinkle geometry, we calculate its effective index as a function of wrinkle width and height for both suspended h^{10}BN and h^{10}BN/calcite structures (**Fig. R3**). We find that both the real and imaginary parts of the M_g effective index evolve continuously with wrinkle geometry. Above a critical wrinkle height of ~50 nm, the M_g mode exhibits nearly identical dispersion trends in both suspended and substrate-supported configurations, indicating that the guided mode is primarily governed by the wrinkle geometry and thus supporting the waveguide interpretation.

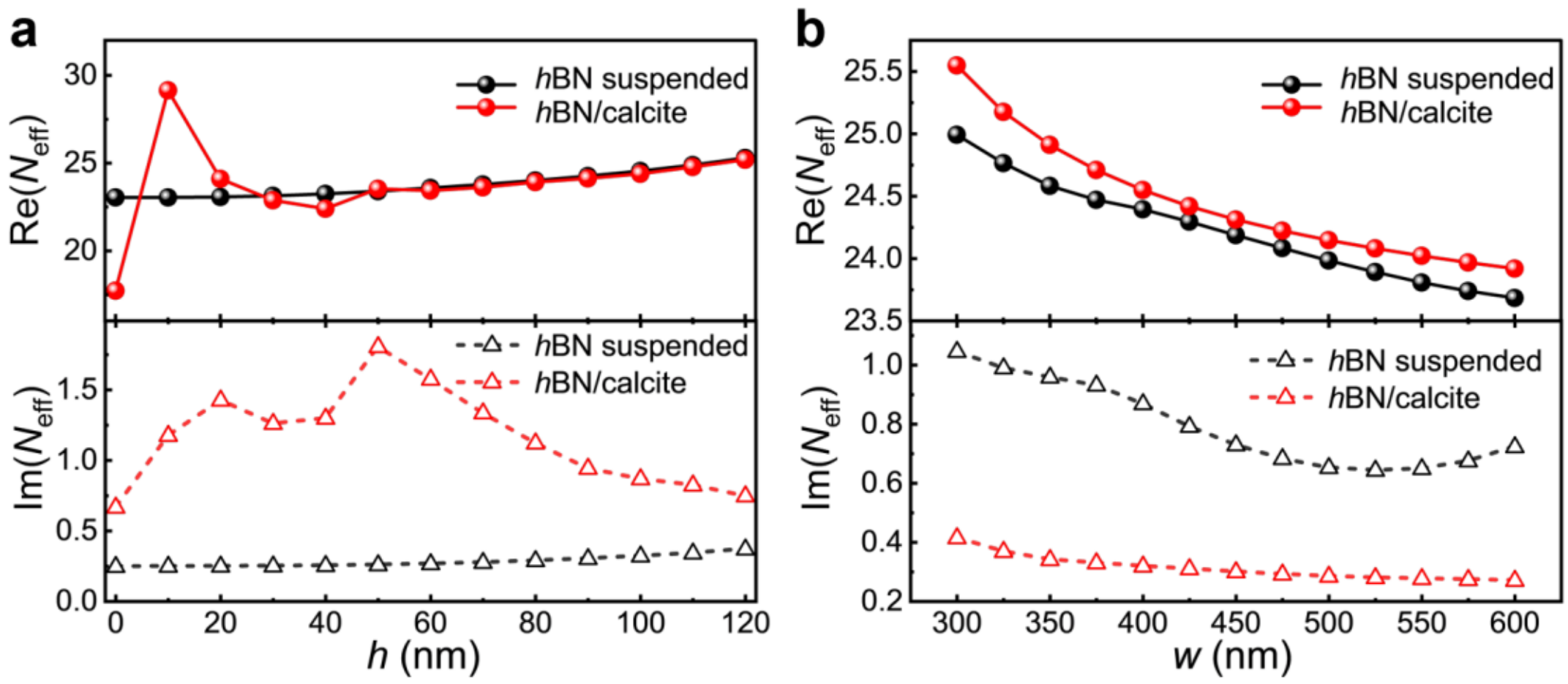

Figure R3 | Evolution of the wrinkle-guided M_g mode with wrinkle geometry. (a) Real (solid circles) and imaginary (open triangles) parts of the effective refractive index N_{eff} of the M_g mode as a function of wrinkle height h for suspended hBN (black) and hBN/calcite (red) structures. (b) Real (solid circles) and imaginary (open triangles) parts of N_{eff} of the M_g mode as a function of wrinkle width w for suspended hBN (black) and hBN/calcite (red) structures.

We further note that the confinement of M_g is influenced by momentum-space hybridization with the substrate-supported M_2 branch, but can be systematically engineered by tuning the h^{10}BN thickness — reducing the thickness from 60 nm to 30 nm suppresses the hybridization and increases the confinement ratio η_{Mg} to 93.1% (**Fig. R4**).

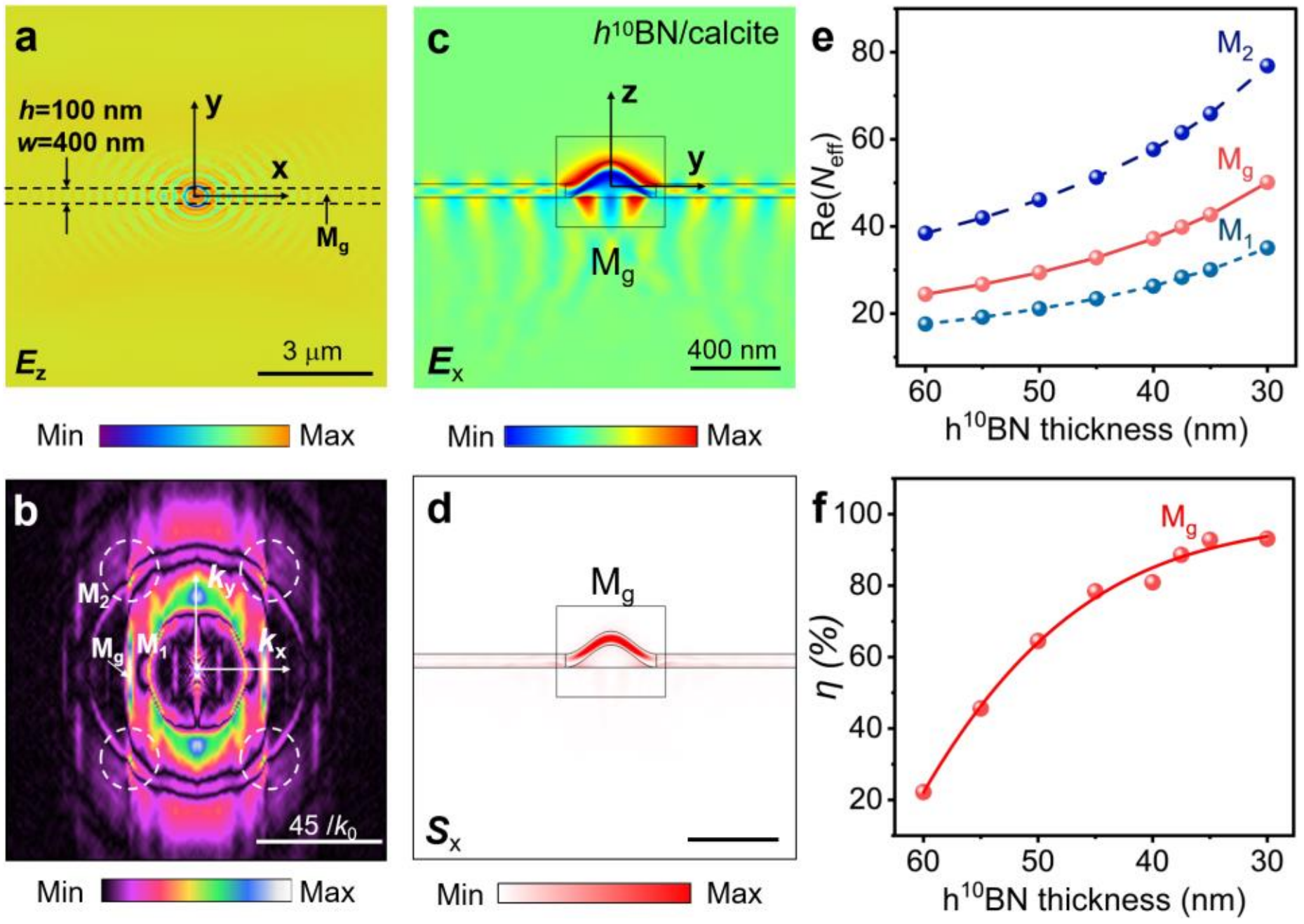

Figure R4 | Power-flow confinement and thickness-dependent tunability of wrinkle-guided polariton modes in *h*^{10}BN. (a) Simulated wavefronts at the surface of a wrinkled *h*^{10}BN nanosheet, the width of the wrinkle *w* is 400 nm and the height *h* is 100 nm (b) Fourier transform of (a), M_g is a wrinkle-guided mode. The circled region highlights the overlap between the wrinkle-guided M_g mode and the substrate-supported M_2 mode. (c) Cross-sectional mode profile of M_g, the component of the optical field E_x is shown. (d) Cross-sectional $\boldsymbol{S}_x$ distribution of the M_g mode. (e) Real part of the effective refractive index $Re(N_{eff})$ of the M_g wrinkle-guided mode as a function of *h*^{10}BN thickness. (f) Confinement ratio η_{Mg} as a function of *h*^{10}BN thickness.

To further illustrate this tunability, we extended the analysis of the wrinkled graphene system previously presented in the manuscript (Supplementary Fig. 14) by performing FFT filtering (**Fig. R5**e, f) and cross-sectional power-flow analysis (**Fig. R5**h). Since graphene supports only a single plasmonic branch, hybridization with substrate modes is suppressed, and the wrinkle-guided plasmon mode propagates tightly confined along the wrinkle axis with η_{Mg}=90.0%, supporting the generalizability of our thermomechanical strategy to other thin-film material systems.

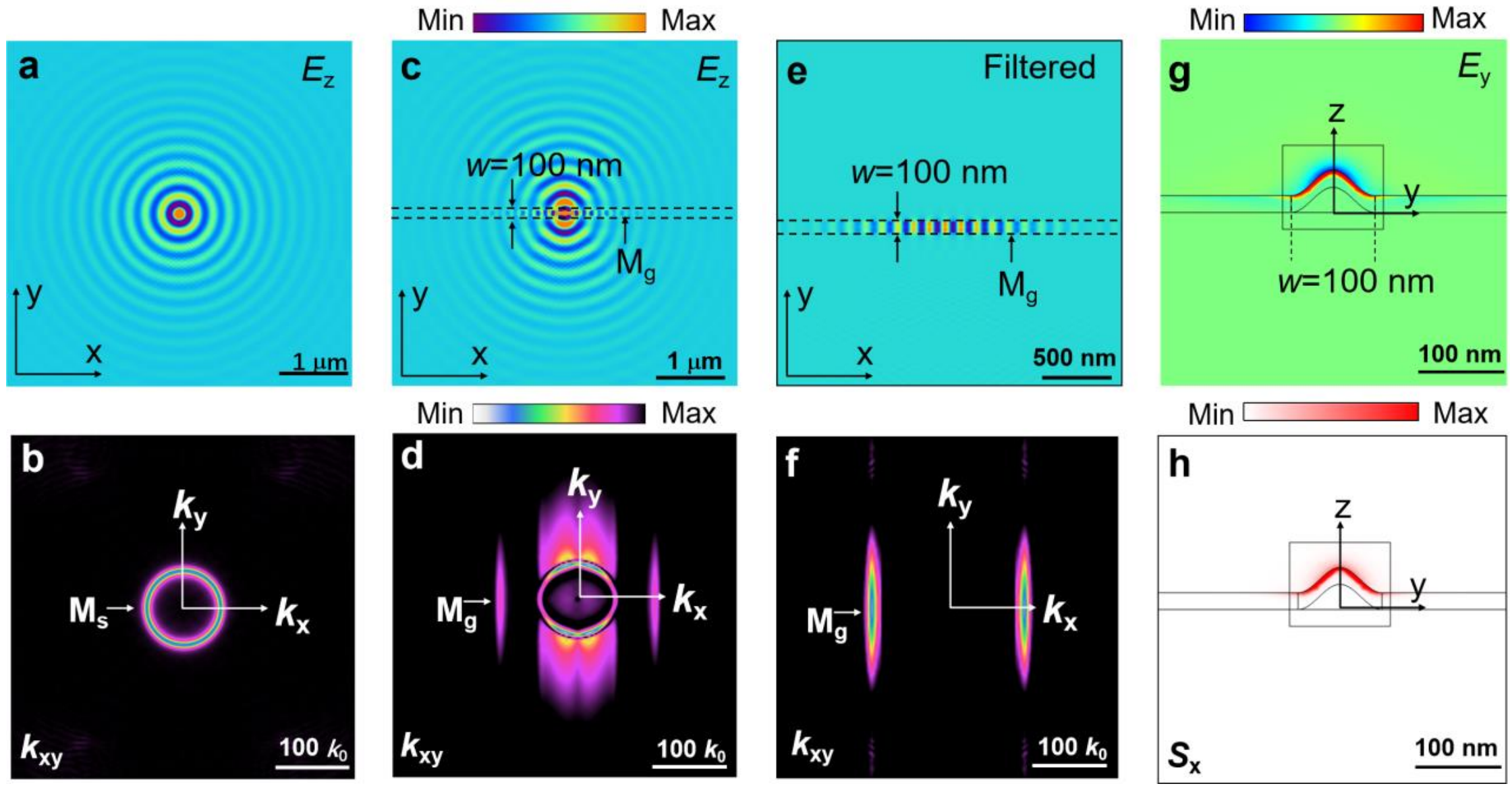

Figure R5 | Channeling of a plasmon polariton mode (M_g) by a wrinkled graphene nanosheet on *h*BN/calcite composite substrates. **a**, A simulated snapshot of the plasmon polariton wavefronts launched by a *z*-oriented dipole located 100-nm above the surface of a monolayer graphene, the excitation frequency is 860 cm^{-1}. **b**, Fourier transform of (**a**), indicating the plasmon polariton mode (M_s, $k_{Ms,x}$=48.75k_0) has been excited by the dipole. **c**, Simulated wavefronts at the surface of a wrinkled graphene, which is formed by the underlying *h*BN wrinkle. The width of the wrinkle *w* is 100 nm, and the height *h* is 30 nm. The dipole is located 100 nm above the crest of the wrinkle. A new plasmon polariton mode (M_g) can be identified. This mode propagates in the ±*x* direction and has a significantly shorter wavelength compared to M_s. **d**, Fourier transform of (**c**). It clearly shows that the M_g mode ($k_{Mg,x}$=104.58k_0) in the *x*-direction has a much larger wavevector than the M_s mode. **e**, Filtered real-space image of the simulated electric field after applying a spatial frequency filter to the Fourier transform in (**c**). **f**, Corresponding filtered momentum-space map of (**e**). **g**, Cross-sectional mode profile of M_g in (**e**), with the in-plane component of the optical field E_y shown. **h**, Corresponding cross-sectional real part of $\boldsymbol{S}_x$ distributions of the M_g mode.

To resolve the concerns of the reviewer, we have made the following changes in the revised manuscript:

(1) revised the wording: "corresponding to a 1/e amplitude decay length of L = 0.68 μm. For comparison, line profiles from the flat region outside the wrinkle resolve two distinct fringe periods corresponding to $M_{1,h}$ (Q = 27.4, L = 1.56 μm) and $M_{1,t}$ (Q = 18.1, L = 0.50 μm), confirming that the M_g mode carries propagation characteristics distinct from the conventional substrate-supported modes" in the main text (**page 9, paragraph 1**);

(2) revised the wording: "Wrinkling of the h^{10}BN nanosheet (400 nm width and 100 nm height) breaks the in-plane symmetry of the heterostructure, leading to a redistribution of polariton wavefronts in real space (Fig. 2c) and a reconfiguration of the isofrequency contours (Fig. 2d). This geometric perturbation gives rise to a distinct M_g mode (clearly resolved in filtered real-space images with M_1 and M_2 components filtered out, Supplementary Fig. 6), which

propagates predominantly along the $\pm x$ direction with an in-plane wavevector of $24.58k_0$, clearly separated from the substrate-supported M_1 and M_2 modes. Cross-sectional field profiling confirms that the M_g mode is localized within the suspended wrinkle region (Fig. 2e), consistent with a geometry-confined propagation channel. The guided nature of the M_g mode is reflected in its systematic dependence on wrinkle geometry, with both the real and imaginary parts of the effective index N_{eff} evolving continuously as functions of wrinkle height h and width w (Fig. 2f and Supplementary Fig. 7). Notably, for $h > 50$ nm, the M_g mode exhibits nearly identical dispersion trends in both suspended and substrate-supported configurations. Collectively, these observations—its distinct wavevector, characteristic isofrequency contour, and geometry-dependent dispersion—support the interpretation of M_g as a wrinkle-guided channeling mode." in the main text (**page 6, paragraph 3**);

(3) added **Fig. R3** to the Supplementary Information as **Supplementary Fig. 7**.

(4) added the following explanation "While the existence of the M_g mode is determined by the wrinkle geometry, its confinement is influenced by coupling to substrate-supported modes. As shown in **Fig. 2**d, the isofrequency contours of M_g partially overlap with those of the second-order substrate-supported mode M_2, inducing hybridization between the two branches. This hybridization redistributes polariton energy and thereby provides a route for engineering modal confinement through h^{10}BN thickness tuning. As shown in Supplementary Fig. 8, reducing the h^{10}BN thickness from 60 nm to 30 nm shifts the M_g and M_2 modes to larger wavevectors and decreases the overlap of their isofrequency contours, thereby weakening mode hybridization and increasing the M_g mode confinement ratio η to 93.1% ." to the main text (**page 7, paragraph 2**);

(5) added **Figure R2c-f** to the Supplementary Information as **Supplementary Fig. 8**.

(6) replaced **Supplementary Fig. 14** with **Fig. R5**.

List of changes

(in the order of appearance in the revised manuscript)

(1) added "School of Materials Science and Engineering, Shanghai Jiao Tong University, Shanghai, China" as the second affiliation for *Peining Li* (**Page 1**);

(2) revised the wording in the **Abstract**: "Cryogenic near-field imaging and simulations reveal a wrinkle-guided polariton mode with a wavevector and dispersion governed by the wrinkle geometry and distinct from substrate-supported branches. Their hybridization allows precise tuning of modal confinement via *h*BN thickness variation." (**Page 1**);

(3) added the following explanation "The wrinkle-on-substrate platform introduces hybridization between wrinkle-guided and substrate-supported modes as an additional degree of freedom, offering new opportunities for tuning modal confinement." to the main text (**page 2, paragraph 2**);

(4) revised the wording "Wrinkling of the h^{10}BN nanosheet (400 nm width and 100 nm height) breaks the in-plane symmetry of the heterostructure, leading to a redistribution of polariton wavefronts in real space (Fig. 2c) and a reconfiguration of the isofrequency contours (Fig. 2d). This geometric perturbation gives rise to a distinct M_g mode (clearly resolved in filtered real-space images with M_1 and M_2 components filtered out, Supplementary Fig. 6), which propagates predominantly along the $\pm x$ direction with an in-plane wavevector of $24.58k_0$, clearly separated from the substrate-supported M_1 and M_2 modes. Cross-sectional field profiling confirms that the M_g mode is localized within the suspended wrinkle region (Fig. 2e), consistent with a geometry-confined propagation channel. The guided nature of the M_g mode is reflected in its systematic dependence on wrinkle geometry, with both the real and imaginary parts of the effective index N_{eff} evolving continuously as functions of wrinkle height h and width w (Fig. 2f and Supplementary Fig. 7). Notably, for $h > 50$ nm, the M_g mode exhibits nearly identical dispersion trends in both suspended and substrate-supported configurations. Collectively, these observations—its distinct wavevector, characteristic isofrequency contour, and geometry-dependent dispersion—support the interpretation of M_g as a wrinkle-guided channeling mode." in the main text (**page 6, paragraph 3**);

(5) added the following explanation "While the existence of the M_g mode is determined by the wrinkle geometry, its confinement is influenced by coupling to substrate-supported modes. As shown in **Fig. 2**d, the isofrequency contours of M_g partially overlap with those of the second-

order substrate-supported mode M_2, inducing hybridization between the two branches. This hybridization redistributes polariton energy and thereby provides a route for engineering modal confinement through h^{10}BN thickness tuning. As shown in Supplementary Fig. 8, reducing the h^{10}BN thickness from 60 nm to 30 nm shifts the M_g and M_2 modes to larger wavevectors and decreases the overlap of their isofrequency contours, thereby weakening mode hybridization and increasing the M_g mode confinement ratio η to 93.1%." to the main text (**page 7, paragraph 2**);

(6) replaced **Fig. 2**d with **Fig. R2**b;

(7) added the following explanation "The circled region highlights the overlap between the wrinkle-guided M_g mode and the substrate-supported M_2 mode." to the **Fig. 2** caption (**page 8**);

(8) changed the Supplementary Figure number from 7 to 9 in the main text (**page 9, paragraph 1**);

(9) revised the wording "corresponding to a 1/e amplitude decay length of L = 0.68 μm. For comparison, line profiles from the flat region outside the wrinkle resolve two distinct fringe periods corresponding to $M_{1,h}$ (Q = 27.4, L = 1.56 μm) and $M_{1,t}$ (Q = 18.1, L = 0.50 μm), confirming that the M_g mode carries propagation characteristics distinct from the conventional substrate-supported modes" in the main text (**page 9, paragraph 1**);

(10) changed the Supplementary Figure number from 8 to 10 in the main text (**page 9, paragraph 1**);

(11) changed the Supplementary Figure number from 9 to 11 in the main text (**page 10, paragraph 1**);

(12) revised "tightly localized" to "concentrated" for wording precision in the main text (**page 11, paragraph 1**);

(13) changed the Supplementary Figure number from 10 to 12 in the main text (**page 12, paragraph 1**);

(14) changed the Supplementary Figure number from 11 to 13 in the main text (**page 12, paragraph 3**);

(15) changed the Supplementary Figure number from 12 to 14 in the main text (**page 13, paragraph 2**);

(16) changed the Supplementary Figure number from 13 to 15 in the main text (**page 13, paragraph 4**);

(17) revised the wording in the **Conclusions**: “Using cryo-SNOM imaging and full electromagnetic simulations, we demonstrate that these buckled surfaces operate as channel waveguides sustaining wrinkle-guided polariton modes that propagate along predefined directional pathways. The wrinkle-guided mode exhibits dispersion distinct from substrate-supported branches, and hybridization between these two modes delivers a versatile route to engineer modal confinement via h^{10}BN thickness tuning.”(**Page 13-14**);

(18) added Prof. G. W. Hu (Nanyang Technological University) and Prof. C. W. Qiu (National University of Singapore) to the **Acknowledgements** section for helpful discussions (**Page 18**);

(19) changed the Supplementary Figure number from 5,7 to 5-9 in the **Supplementary information** (**page 3**);

(20) changed the Supplementary Figure number from 12 to 14 in the **Supplementary information** (**page 3**);

(21) added **Fig. R1** to the Supplementary Information as **Supplementary Fig. 7**;

(22) added the following explanation “The confinement ratio is defined as:

$$\eta = \frac{P_{\text{wrinkle}}}{P_{\text{total}}} \times 100\% = \frac{\iint_{\text{wrinkle}} S_x(y,z)dydz}{\iint_{\text{A}} S_x(y,z)dydz} \times 100\%$$

where S_x is the time-averaged Poynting vector component along the propagation direction, P_{wrinkle} is the power flowing within the wrinkle region (as indicated by the rectangular region in Supplementary Fig. 8b), and P_{total} is the total power crossing the entire transverse section.”to the **Supplementary Information (page 11);**

(23) added **Fig. R2c-f** to the Supplementary Information as **Supplementary Fig. 8**;

(24) replaced **Supplementary Fig. 14** with **Fig. R5**.
